# Supplementary material for: Impact of COVID-19 on acute trauma and orthopaedic referrals and surgery in the UK during the first wave of the pandemic: a multicentre observational study from the COVid Emergency-Related Trauma and orthopaedics (COVERT) Collaborative
Source: BMJ Open. 2022 Jan 18;12(1):e054919. doi: 10.1136/bmjopen-2021-054919 (PMC8771810; doi:10.1136/bmjopen-2021-054919)
Supplement: Supplementary data [file bmjopen-2021-054919supp001.pdf]

Appendix 1: Post-operative complications for both years

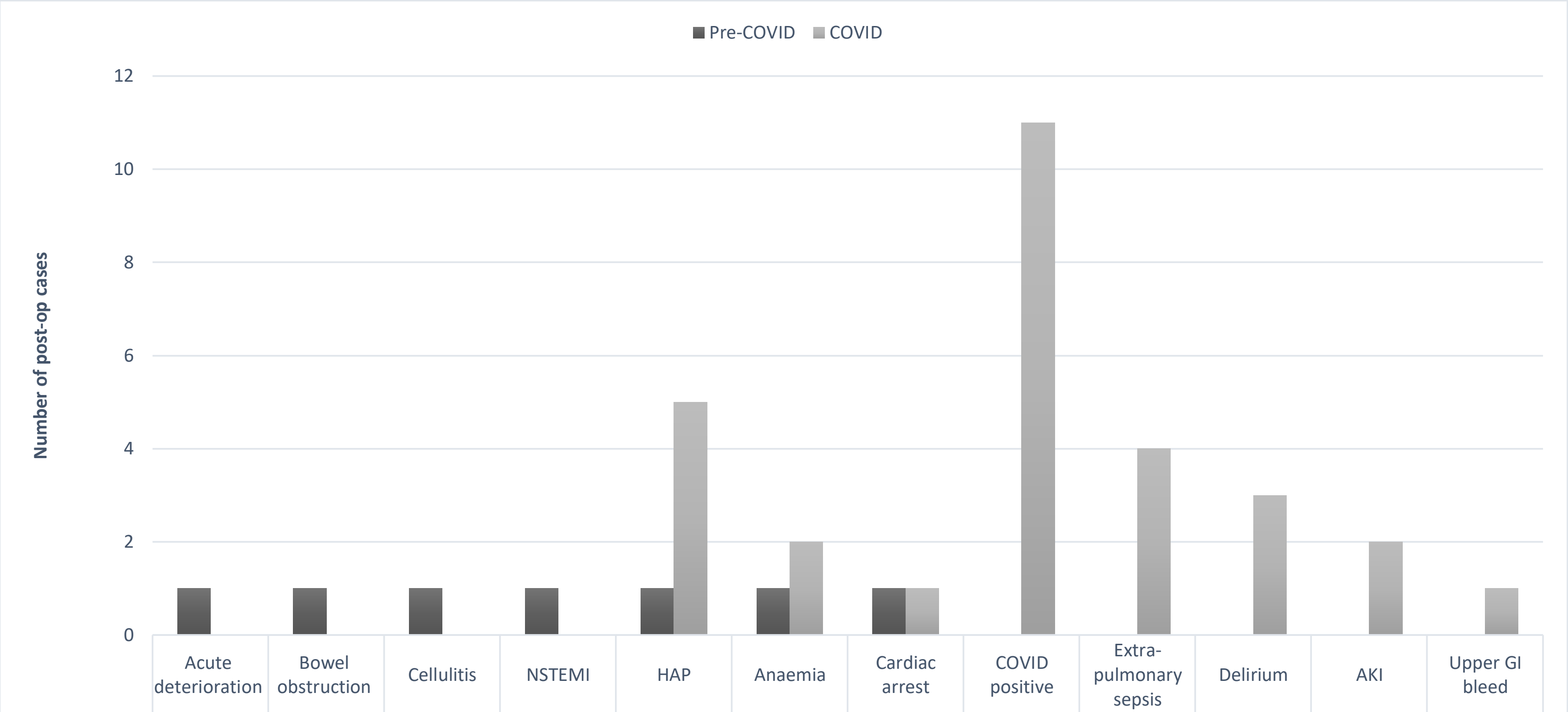

	Acute deterioration	Bowel obstruction	Cellulitis	NSTEMI	HAP	Anaemia	Cardiac arrest	COVID positive	Extra-pulmonary sepsis	Delirium	AKI	Upper GI bleed
Pre-COVID	1	1	1	1	1	1	1	0	0	0	0	0
COVID	0	0	0	0	5	2	1	11	4	3	2	1

***Key**: NSTEMI: non-ST elevated myocardial infarction, HAP: hospital-acquired pneumonia, AKI: acute kidney injury, GI: gastrointestinal*
