# Supplementary figures and images for: Impact of COVID-19 on acute trauma and orthopaedic referrals and surgery in the UK during the first wave of the pandemic: a multicentre observational study from the COVid Emergency-Related Trauma and orthopaedics (COVERT) Collaborative
Source: BMJ Open. 2022 Jan 18;12(1):e054919. doi: 10.1136/bmjopen-2021-054919 (PMC8771810; doi:10.1136/bmjopen-2021-054919)

Appendix 2: Type of comorbidities for all mortalities relative to both years

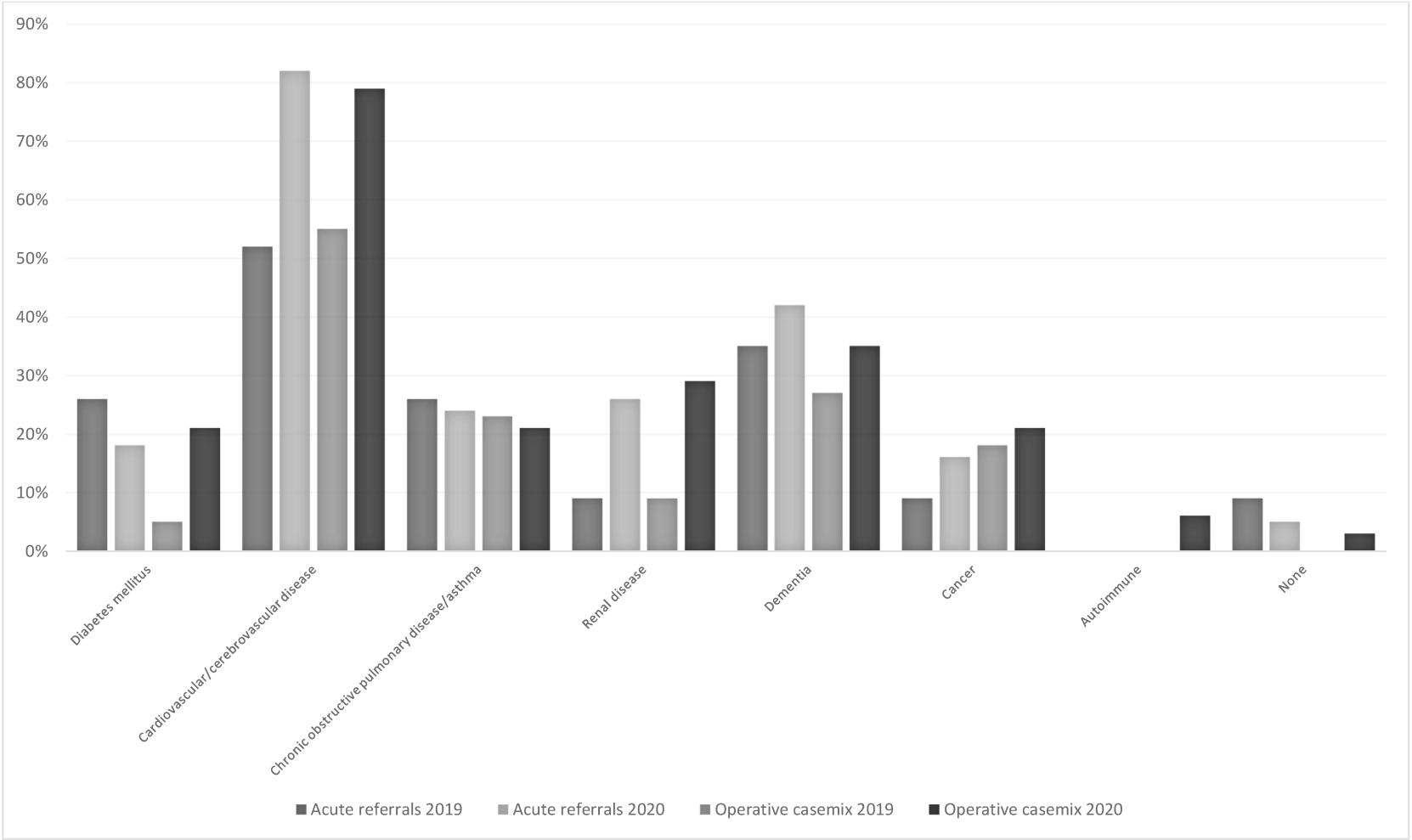

Supplement: Supplementary data [file bmjopen-2021-054919supp002.pdf]

Appendix 3: number of comorbidities for all mortalities relative to both years

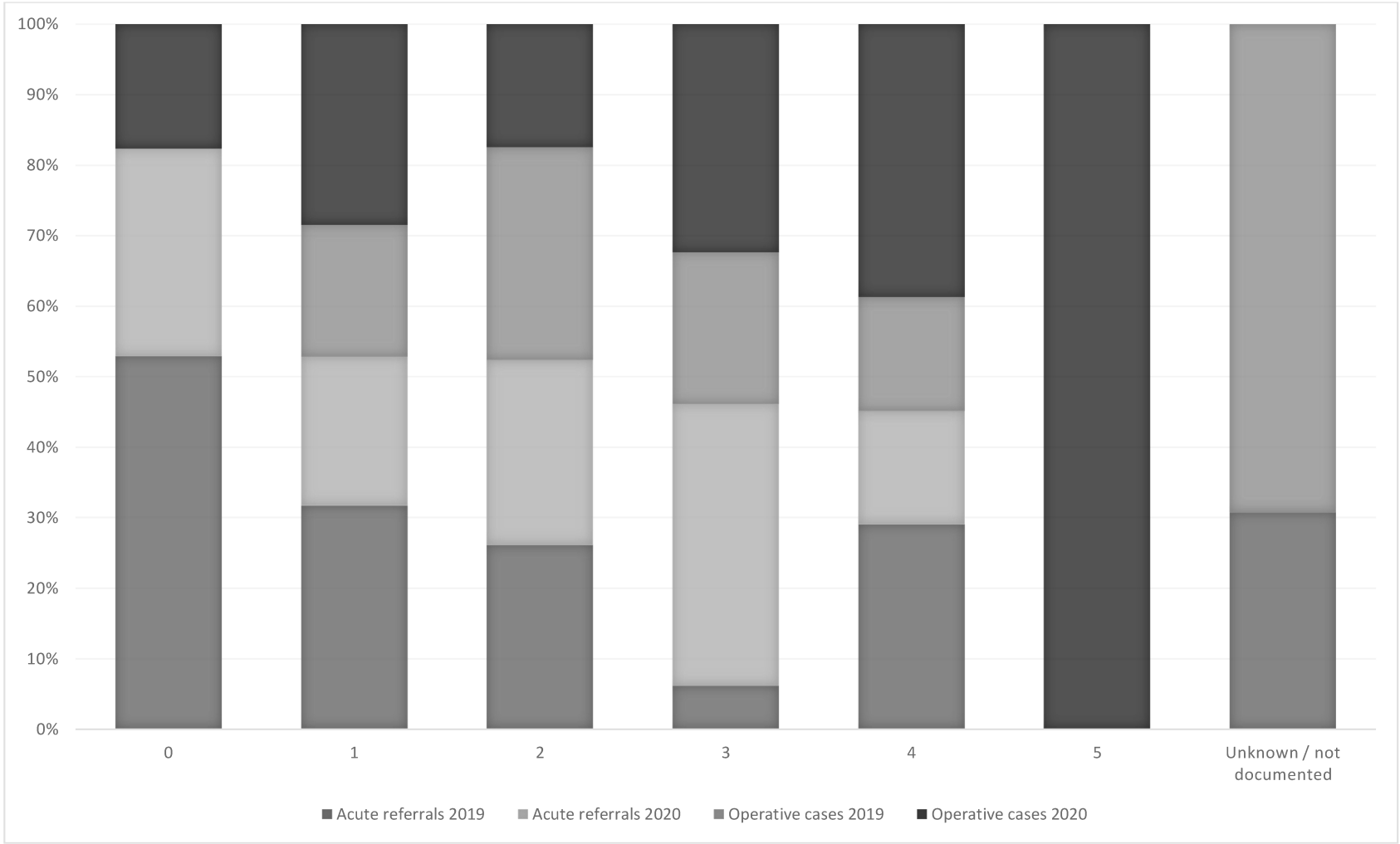

Supplement: Supplementary data [file bmjopen-2021-054919supp003.pdf]
