# Supplementary material for: Impact of COVID-19 on acute trauma and orthopaedic referrals and surgery in the UK during the first wave of the pandemic: a multicentre observational study from the COVid Emergency-Related Trauma and orthopaedics (COVERT) Collaborative
Source: BMJ Open. 2022 Jan 18;12(1):e054919. doi: 10.1136/bmjopen-2021-054919 (PMC8771810; doi:10.1136/bmjopen-2021-054919)
Supplement: Author's manuscript [file bmjopen-2021-054919.draft_revisions.pdf]

BMJ Open

BMJ Open is committed to open peer review. As part of this commitment we make the peer review history of every article we publish publicly available.

When an article is published we post the peer reviewers' comments and the authors' responses online. We also post the versions of the paper that were used during peer review. These are the versions that the peer review comments apply to.

The versions of the paper that follow are the versions that were submitted during the peer review process. They are not the versions of record or the final published versions. They should not be cited or distributed as the published version of this manuscript.

BMJ Open is an open access journal and the full, final, typeset and author-corrected version of record of the manuscript is available on our site with no access controls, subscription charges or pay-per-view fees (http://bmjopen.bmj.com).

If you have any questions on BMJ Open's open peer review process please email info.bmjopen@bmj.com

BMJ Open

The impact of COVID-19 on acute Trauma and Orthopaedic referrals and surgery in the UK during the 'peak weeks': lessons learnt from the largest and longest national multi-centre observational study.

Journal:	*BMJ Open*
Manuscript ID	bmjopen-2021-054919
Article Type:	Original research
Date Submitted by the Author:	03-Jul-2021

Complete List of Authors:	Sugand, Kapil; Imperial College London, MSk Lab<br>Aframian, Arash; Imperial College London, MSk Lab<br>Park, Chang; Imperial College London, Trauma and orthopaedics<br>Collaborative, COVERT; Imperial College London<br>Sarraf , Khaled; Imperial College London, Trauma and Orthopaedics

Keywords:	COVID-19, TRAUMA MANAGEMENT, ORTHOPAEDIC & TRAUMA SURGERY, EPIDEMIOLOGY

SCHOLARONE™ Manuscripts

I, the Submitting Author has the right to grant and does grant on behalf of all authors of the Work (as defined in the below author licence), an exclusive licence and/or a non-exclusive licence for contributions from authors who are: i) UK Crown employees; ii) where BMJ has agreed a CC-BY licence shall apply, and/or iii) in accordance with the terms applicable for US Federal Government officers or employees acting as part of their official duties; on a worldwide, perpetual, irrevocable, royalty-free basis to BMJ Publishing Group Ltd ("BMJ") its licensees and where the relevant Journal is co-owned by BMJ to the co-owners of the Journal, to publish the Work in this journal and any other BMJ products and to exploit all rights, as set out in our licence.

The Submitting Author accepts and understands that any supply made under these terms is made by BMJ to the Submitting Author unless you are acting as an employee on behalf of your employer or a postgraduate student of an affiliated institution which is paying any applicable article publishing charge ("APC") for Open Access articles. Where the Submitting Author wishes to make the Work available on an Open Access basis (and intends to pay the relevant APC), the terms of reuse of such Open Access shall be governed by a Creative Commons licence – details of these licences and which Creative Commons licence will apply to this Work are set out in our licence referred to above.

Other than as permitted in any relevant BMJ Author's Self Archiving Policies, I confirm this Work has not been accepted for publication elsewhere, is not being considered for publication elsewhere and does not duplicate material already published. I confirm all authors consent to publication of this Work and authorise the granting of this licence.

For peer review only

The impact of COVID-19 on acute Trauma and Orthopaedic referrals and surgery in the UK during the 'peak weeks': lessons learnt from the largest and longest national multi-centre observational study.

The COVid-Emergency Related Trauma and orthopaedics (COVERT) Collaborative

Core authorship committee

Name	Role	Qualification	Contribution	E-mail
**Kapil Sugand** Primary & corresponding author *58 Thames Avenue, Greenford, Middlesex, UB6 8JN* *E: ks704@ic.ac.uk* *T: 00447773642813*	T&O Specialist Trainee	MRCS	• Conceptualisation<br>• Data curation<br>• Formal analysis<br>• Investigation<br>• Methodology<br>• Project administration<br>• Resources<br>• Supervise<br>• Validation<br>• Visualisation<br>• Writing original draft<br>• Writing – reviewing and editing	Ks704@ic.ac.uk<br><br>ORCID: 0000-0003-3185-954X
Arash Aframian	T&O Specialist Trainee	MRCS	• Conceptualisation<br>• Formal analysis<br>• Investigation<br>• Methodology<br>• Supervise	arash.aframian@gmail.com

For peer review only

			• Writing – reviewing and editing	
Chang Park	T&O Specialist Trainee	MRCS	• Conceptualisation<br>• Methodology<br>• Supervise<br>• Writing – reviewing and editing	changpark3@gmail.com
Khaled M Sarraf (senior author)	T&O Consultant	FRCS	• Conceptualisation<br>• Investigation<br>• Methodology<br>• Project administration<br>• Resources<br>• Supervise<br>• Writing – reviewing and editing	khaled.sarraf@nhs.net

COVERT Collaborators

1. **Department of Trauma and Orthopaedics, Imperial College Healthcare NHS Trust, London, W2 1NY, England**

Name	Role	Qualification	Contribution	E-mail
Thomas Ashdown	Core Surgical Trainee	MRCS	• Data curation<br>• Formal analysis<br>• Resources	thomas.ashdown@nhs.net
Ramla Ali	Foundation Doctor	MBBS	• Data curation<br>• Formal analysis<br>• Resources	ramla.ali@nhs.net

For peer review only - http://bmjopen.bmj.com/site/about/guidelines.xhtml

William Harland	Foundation Doctor	MBBS	• Data curation<br>• Formal analysis<br>• Resources	william.harland@nhs.net
Charles Hallett	Foundation Doctor	MBBS	• Data curation<br>• Formal analysis<br>• Resources	Ortho@pansurg.org
Chinmay Gupte	T&O Consultant	FRCS	• Project administration<br>• Resources<br>• Supervise	c.gupte00@ic.ac.uk
Dominic Spicer	T&O Consultant	FRCS	• Project administration<br>• Resources<br>• Supervise	dominic.spicer@nhs.net
Robin Strachan	T&O Consultant	FRCS	• Project administration<br>• Resources<br>• Supervise	robin.strachan@nhs.net
Dinesh Nathwani	T&O Consultant	FRCS	• Project administration<br>• Resources<br>• Supervise	d.nathwani@imperial.ac.uk
Rajarshi Bhattacharya	T&O Consultant	FRCS	• Project administration<br>• Resources<br>• Supervise	rajarshi.bhattacharya@nhs.net

For peer review only - http://bmjopen.bmj.com/site/about/guidelines.xhtml

2. **Department of Trauma and Orthopaedics, Chelsea and Westminster Hospital NHS Foundation Trust, London, SW10 9NH, England**

**Name**	**Role**	**Qualification**	**Contribution**	**E-mail**
Catrin Morgan	T&O Specialist Trainee	MRCS	• Data curation<br>• Formal analysis<br>• Project administration<br>• Supervise	catrin.morgan@nhs.net
Katharine Bennett-Brown	Core Surgical Trainee	MRCS	• Data curation<br>• Formal analysis<br>• Resources	katebennettbrown@gmail.com
Henry Simon	T&O Specialist Trainee	MRCS	• Data curation<br>• Formal analysis<br>• Resources	henrysimon@hotmail.co.uk
Rupen Dattani	T&O Consultant	FRCS	• Project administration<br>• Resources<br>• Supervise	rupen.dattani@nhs.net

3. **Department of Trauma and Orthopaedics, Queen Elizabeth Hospital, Gateshead Health NHS Foundation Trust, Gateshead, NE9 6SX, England**

**Name**	**Role**	**Qualification**	**Contribution**	**E-mail**
Adham Mousa	T&O Specialist Trainee	MRCS	• Data curation<br>• Formal analysis<br>• Project administration<br>• Supervise	adham.mousa@nhs.net
Karen Vejsbjerg	Core Surgical Trainee	MRCS	• Data curation<br>• Formal analysis<br>• Resources	karen.vejsbjerg1@nhs.net
Michael Zhang	Foundation Doctor	MBBS	• Data curation<br>• Formal analysis	michael.zhang1@nhs.net

For peer review only

For peer review only - http://bmjopen.bmj.com/site/about/guidelines.xhtml

			• Resources	
Vikas Kumar	Foundation Doctor	MBBS	• Data curation<br>• Formal analysis<br>• Resources	vikas.kumar7@nhs.net
Hannah Emerson	Foundation Doctor	MBBS	• Data curation<br>• Formal analysis<br>• Resources	hannah.emerson2@nhs.net
Andrew Oliver	Foundation Doctor	MBBS	• Data curation<br>• Formal analysis<br>• Resources	andrew.oliver2@nhs.net
Callum Smith	Foundation Doctor	MBBS	• Data curation<br>• Formal analysis<br>• Resources	callum.smith8@nhs.net
Andreas Hinsche	T&O Consultant	FRCS	• Project administration<br>• Resources<br>• Supervise	a.hinsche@nhs.net

4. Department of Trauma and Orthopaedics, Darent Valley Hospital, Dartford and Gravesham NHS Trust, Dartford, DA2 8DA, England

**Name**	**Role**	**Qualification**	**Contribution**	**E-mail**
Markus L Sagmeister	T&O Specialist Trainee	MRCS	• Data curation<br>• Formal analysis<br>• Project administration<br>• Supervise	markus.sagmeister@nhs.net
Gihan Jayasinghe	T&O Specialist Trainee	MRCS	• Data curation<br>• Formal analysis<br>• Resources	gihan.jayasinghe@nhs.net
Marissa Lewis	Foundation Doctor	MBBS	• Data curation	marissa.lewis2@nhs.net

For peer review only - http://bmjopen.bmj.com/site/about/guidelines.xhtml

			• Formal analysis<br>• Resources	
Michael Avery	Foundation Doctor	MBBS	• Data curation<br>• Formal analysis<br>• Resources	michael.avery@nhs.net

5. **Department of Trauma and Orthopaedics, Royal Berkshire NHS Foundation Trust, Reading, RG1 5AN, England**

**Name**	**Role**	**Qualification**	**Contribution**	**E-mail**
Tariq Aboelmagd	T&O Specialist Trainee	MRCS	• Data curation<br>• Formal analysis<br>• Project administration<br>• Supervise	taboelmagd@doctors.org.uk
Shireen Ibish	T&O Specialist Trainee	MRCS	• Data curation<br>• Formal analysis<br>• Resources	shireen.ibish@nhs.net
Ruben Thumbadoo	Core Surgical Trainee	MRCS	• Data curation<br>• Formal analysis<br>• Resources	ruben.thumbadoo@nhs.net
Asanka Wijendra	T&O Specialist Trainee	MRCS	• Data curation<br>• Formal analysis<br>• Resources	awijendra@gmail.com
Joseph Dixon	T&O Specialist Trainee	MRCS	• Data curation<br>• Formal analysis<br>• Resources	joedixon@doctors.org.uk

For peer review only - http://bmjopen.bmj.com/site/about/guidelines.xhtml

Vatsal Gupta	T&O Specialist Trainee	MRCS	• Data curation<br>• Formal analysis<br>• Resources	vatsalg001@gmail.com

30

31 6. **Department of Trauma and Orthopaedics, James Cook University Hospital, South**
32 **Tees NHS Foundation Trust, Middlesbrough, TS4 3BW, England**

**Name**	**Role**	**Qualifi-cation**	**Contribution**	**E-mail**
Laura Clifton	Core Surgical Trainee	MRCS	• Data curation<br>• Formal analysis<br>• Project administration<br>• Supervise	laura.clifton8@nhs.net
Suhib Taher	Core Surgical Trainee	MRCS	• Data curation<br>• Formal analysis<br>• Resources	Suhib.taher@nhs.net
Nicholas Jia Jie Wei	Core Surgical Trainee	MRCS	• Data curation<br>• Formal analysis<br>• Resources	nicholas.wei1@nhs.net
Emma Reay	T&O Consultant	FRCS	• Data curation<br>• Formal analysis<br>• Resources	emma.reay1@nhs.net

33

For peer review only - http://bmjopen.bmj.com/site/about/guidelines.xhtml

7. Department of Trauma and Orthopaedics, Aneurin Bevan University Health Board, Royal Gwent Hospital, Newport, NP20 2UB, Wales

Name	Role	Qualification	Contribution	E-mail
Ashish Khurana	T&O Consultant	FRCS	• Data curation<br>• Formal analysis<br>• Project administration<br>• Supervise	ashish.khurana@wales.nhs.uk
Kathryn Rooney	T&O Specialist Trainee	MRCS	• Data curation<br>• Formal analysis<br>• Resources	Kathryn.Rooney@wales.nhs.uk
Nakanda Kakaire	Core Surgical Trainee	MRCS	• Data curation<br>• Formal analysis<br>• Resources	nakandamiriam.kakaire@wales.nhs.uk
Oluwawyemisi Adesina	Core Surgical Trainee	MRCS	• Data curation<br>• Formal analysis<br>• Resources	quentinpz@yahoo.com

Informed consent: not applicable – addressed in Ethics section and observational study

Conflicting interest: Nil

Financial disclosure: Nil

Acknowledgements: Nil

Word count: 4000 words

For peer review only - http://bmjopen.bmj.com/site/about/guidelines.xhtml

Keywords: *COVID-19; orthopaedic trauma; UK multi-centre; pandemic wave; mortality*

Contributor & guarantor information

The corresponding author attests that all listed authors meet authorship criteria and that no others meeting the criteria have been omitted. All listed authors from all centres have contributed by mining and analysing data. KS, AA, CP and KMS conceptualised the study and agreed on the methodology. KS wrote the first draft and analysed the entire dataset. The dataset was then reviewed for accuracy by all authors within Imperial College Healthcare NHS Trust as well as contributing to subsequent drafts of the manuscripts. The final version was reviewed and signed off by each Consultant representative from each contributing centre. KS finalised the submission, and he is both the primary and corresponding author.

For peer review only

For peer review only - http://bmjopen.bmj.com/site/about/guidelines.xhtml

Abstract

Objective: This is the first national study observing the impact of the COVID-19 pandemic on orthopaedic trauma with respect to referrals, operative caseload and mortality during its peak.

Design: A longitudinal, national, multi-centre, retrospective, observational, cohort study was conducted for 6 weeks (namely the 'peak weeks') from March 17, 2020 compared to the same period in 2019.

Setting: Hospitals from seven major urban cities were recruited around the UK, including London.

Participants: A total of 4840 clinical encounters were initially recorded. 4668 clinical encounters were analysed post-exclusion.

Primary and secondary outcome measures: Primary outcomes included the number of acute trauma referrals and those undergoing operative intervention, mortality rates, and the proportion of patients contracting COVID-19. Secondary outcomes consisted of the mechanism of injury, type of operative intervention and proportion of aerosolising-generating anaesthesia utilised.

Results: During the COVID-19 period there was a 34% reduction in acute orthopaedic trauma referrals compared to 2019 (1792 down to 1183 referrals), and 29.5% less surgical interventions (993 down to 700 operations). The mortality rate significantly (both statistically and clinically) more than doubled for both risk and odds ratios during the COVID period in all referrals (1.3% vs 3.8%, p=0.0005) and in those undergoing operative intervention (2.2% vs 4.9%, p=0.004). Moreover, mortality due to COVID-related complications (versus non-COVID causes) had greater odds by a factor of at least 20 times. For the operative cohort during COVID, there was a greater odds of aerosolising-generating anaesthesia (including

For peer review only

those with superimposed regional blocks) by three-quarters as well as doubled odds of a Consultant acting as the primary surgeon.

Conclusion: Although there was a reduction of acute trauma referrals and those undergoing operative intervention, the mortality rate still more than doubled in odds during the peak of the pandemic compared to the same time interval one year ago.

Keywords: COVID-19; orthopaedic trauma; UK multi-centre; pandemic wave; mortality

Article summary: Strengths and limitations of this study

- This was the first representative observational study of the UK looking into the impact of COVID-19 pandemic on general Trauma and Orthopaedic surgical specialty.
- There is a valid comparison between two timeframes, exactly one year apart to represent pre-COVID and during COVID.
- Other studies thus far have only shed light on local scales or cross-speciality within a shorter timeframe than this study and not necessarily commenting on mortality rates like this study.
- Weaknesses included loss of data points which have been accounted for in the tables (i.e. labelled as unknown) which did not affect the final analysis of data points.

For peer review only

107 • Operations conducted outside the specific study periods will not account for all those
108 operations required such as for hip fractures.

For peer review only

For peer review only - http://bmjopen.bmj.com/site/about/guidelines.xhtml

Introduction

The Global Impact of COVID-19

The COVID-19 pandemic will be remembered as one of the most unprecedented global health crises in modern history. With over 3 million deaths and over 141 million recorded cases globally, the pandemic has had a permanent impact on healthcare at the time of article submission.[1] The viral outbreak was first reported in December 2019 with the first patient hospitalized in the city of Wuhan, China.[2] By mid-March the outbreak affected over 190 countries with over 450,000 cases and over 20,000 deaths, thus being declared a pandemic and a global public health emergency by the World Health Organization.[3] On January 24th 2020 Europe reported its first case followed by a case in the United Kingdom (UK) 5 days later.[4]

The British Response to the pandemic

The English government responded by implementing social distancing measures on the 17th March 2020 in an attempt to reduce the rate of transmission and therefore the demands on the National Health Service (NHS).[5] This was followed a week later by more stringent measures, commonly referred to as a societal 'lockdown'.[6] As of the 23rd March 2020, all members of the public were required to stay at home. The NHS has also been deeply affected by the strain imposed by the virus as the healthcare infrastructure has had to evolve to cope with the overwhelming and unexpected pressures on staff, resources and finances. There has been a complete renovation of Emergency Medicine and Orthopaedic services to manage musculoskeletal disease and trauma. In response to the NHS emergency declaration,[7] the Royal Colleges of Surgeons[8] and the British Orthopaedic Association[9] both issued statements and guidelines for delivering emergency Trauma and Orthopaedic care during the COVID-19 outbreak. The lockdown to limit the spread of the virus has had an unforeseen effect in

For peer review only - http://bmjopen.bmj.com/site/about/guidelines.xhtml

significantly reducing the acute trauma workload described in several single centre studies.[10-13] There has however not been a national reflection of the impact of the COVID-19 pandemic on the orthopaedic workload and its potential impact on the mortality.

Aim

To observe the impact of COVID-19 on Trauma and Orthopaedic acute referrals, operative casemix and mortality rates during the 'peak weeks' of the pandemic compared to the same time interval in 2019.

Outcomes/objectives

Primary outcomes included the number of acute trauma referrals and those undergoing operative intervention, post-operative complications, mortality rates, and the proportion of patients contracting COVID-19. Secondary outcomes consisted of the mechanism of injury, type of operative intervention and proportion of aerosolising-generating anaesthesia utilised.

Alternative hypothesis

The alternative hypothesis was that when comparing both years, there would be a difference in the prevalence of acute orthopaedic referrals, orthopaedic trauma casemix and aerosol-generating anaesthetic procedures due to social distancing/lockdown. Mortality rates and survival probabilities were also hypothesised to differ due to the first COVID-19 outbreak.

For peer review only - http://bmjopen.bmj.com/site/about/guidelines.xhtml

Methods

Study design: This is the first national, multi-centre longitudinal observational study observing patients who were acutely referred to the Trauma and Orthopaedic departments as well as those operated on within the same six-week interval comparing 2019 to 2020.

Patient sampling: All acute referrals, operative notes, inpatient medical records and discharge summaries were accessed using electronic medical system at each contributing hospital trust.

Study period: The six-week study period was from the start of social distancing on Tuesday 17th March 2020 to Tuesday 31st April 2020 which encompassed the national 'lockdown' measures instigated on the 23rd March 2020. This period was considered the 'golden peak' of the epidemic in the UK. This was compared to the same six-week interval from Tuesday 19th March to Tuesday 30th April 2019 (i.e. prior to any COVID-19 related measures) to compare the impact of the pandemic one year apart.

Inclusion criteria: All acute orthopaedic trauma referrals presenting to the Emergency Department during the intervals one year apart were included. All orthopaedic trauma cases that required an operation, including those from acute orthopaedic trauma referrals, within the intervals one year apart. Those patients listed for an operation prior to time period of data collection were included in the final analysis. We adhered to STROBE guidelines for observational studies.

For peer review only

Exclusion criteria: Any cases being referred internally from other specialties for Trauma and Orthopaedic advice and input, as well as referrals from any external centre asking for tertiary advice were excluded from further analysis. Any patients with post-operative complications arising from the period prior to the data collection were excluded. For operative trauma cases, those undergoing spinal procedures were excluded as these are jointly treated by Neurosurgery in most hospitals. All non-urgent semi-elective procedures were excluded from analysis as well, as they would inaccurately assess the impact of any social distancing measures on the trauma workload. Routine elective orthopaedic cases were excluded.

Data points: Demographics including age, sex and ASA grades were recorded for all patients. Injury characteristics were recorded, including the anatomical location and if the injury was open or closed. The mechanism of injury was categorised and whether the patient was referred as a trauma call. The nature of the operative procedures and the anaesthetic techniques were recorded. Patients undergoing multiple procedures were recorded for every episode where they were taken to theatre. Six-week mortality rate was recorded as well as the COVID-19 status of any symptomatic patients or suspected cases. Data points were divided into acute referrals and operative casemix as seen in table 1.

Table 1: data points for acute referrals and operative casemix

Age (years)	Gender (Male / female by birth)	ASA (1-5)	Date of injury / presentation
Injury	Mechanism of injury	Open Vs Closed fracture	Trauma Call (Yes / No)
Operative procedure	Anaesthetic Technique (AGP vs non-AGP)	Seniority of Surgeon (Consultants vs trainees)	Comorbidities
Six-week mortality	Post-op complications	Surgery time since admission (hours)	COVID status (from PCR swabs)

For peer review only

For peer review only - http://bmjopen.bmj.com/site/about/guidelines.xhtml

- *Anaesthetic techniques:* This was divided into anaesthetic aerosolising-generating procedures (AGP) which consisted of any intubation (including laryngeal mask airway and endotracheal intubation) for a general anaesthetic. All other anaesthetic techniques including regional and local anaesthetics were deemed as non-AGPs.
- *COVID status*: groups of patients were divided into either not swabbed, swabbed due to presence of documented symptoms, negative swabs and positive results.

Statistical analysis: All the data were recorded, anonymised and verified by four members of the study group for their accuracy. The data were processed using Microsoft Excel (Microsoft, Washington, USA). Shapiro-Wilk test indicated a normal distribution for patient demographics. Hence, the mean (± standard deviation; 95% CI) were calculated for both age, ASA grade and days to discharge from hospital. Both prevalence or risk and odds ratios were calculated as well as a Fisher's exact test for statistical significance for categorical data, defined as $p \leq 0.05$. Percentages and confidence intervals were rounded off to one decimal place.

Ethics and permissions: All data points were utilised for routine auditing purposes to reflect departmental activity and service provision without altering clinical care pathways. Each centre contributing data to this study registered their interests with local authority and the auditing or clinical governance departments. No informed consent was required as there was no identifiable data. All data were anonymised at the time of collection and submission. Each patient was assigned a unique identification number which was cross-referenced with the patients' individual hospital identification or medical record numbers. This cross-referenced list remained internally within the hospital trust computer server handled by the contributing team from each trust. The data was transferred and stored using the NHS.net email server which has been approved for transfer of patient data. Data protection compliance was abided

For peer review only - http://bmjopen.bmj.com/site/about/guidelines.xhtml

by at all times. The lead centre was Imperial College Healthcare NHS Trust where this study was first approved as a clinical audit prior to expanding onto a national scale.

All centres gave permission for the use of their data. This study was assessed using the UKRI/MRC/NHS Health Research Authority Ethics Decision Tool and was considered an 'audit/not research'; and therefore it was not subject to further ethical review by the NHS Research Ethics Committee (NHS REC).

Competing interests: The authors have declared that no competing interests exist.

Data sharing statement: Underlying data, code and supporting documentation may be made available as a redacted version to interested parties, subject to the completion of a protocol and signing of a Data Transfer Agreement.

Funding statement: This research received no specific grant from any funding agency in the public, commercial or not-for-profit sectors.

The collaborative: The COVid Emergency Related Trauma and orthopaedics (COVERT) Collaborative was founded at Imperial College Healthcare NHS Trust. It is currently a member of the COVID Research Group and it has been endorsed by the Royal College of Surgeons of England and Imperial College Healthcare NHS Trust.

Patient and Public Involvement: This was a retrospective study observing clinical outcomes. Patients were not involved in the study design, recruitment or conduct. The anonymous data will be disseminated through publication.

For peer review only

Results

A total of 4840 clinical encounters took place between the study periods. A total of 172 spinal operations and presentations were excluded from the final analysis. Table 2 outlined demographic data. Figures 1 and 2 have categorized these clinical encounters into acute referrals and operative cases between both years respectively.

Table 2: Demographic data of pre- and post-COVID

		Pre-COVID (2019)		COVID (2020)	
	**Total**	**1792**		**1183**	
**Acute referrals**	*Male*	935	52.2%	560	47.3%
	Female	857	47.8%	623	52.7%
	Mean age ± SD (95% CI)	52.2 ± 27.9 (50.9 - 53.5)		55.8 ± 27.9 (54.3 - 57.4)	
	**Total**	**993**		**700**	
**Operative cases**	*Male*	499	50.3%	320	45.7%
	Female	494	49.7%	380	54.3%
	Mean Age ± SD (95% CI)	51.7 ± 28.1 (50 - 53.5)		57.7 ± 26.7 (55.7 - 59.6)	
	Mean ASA ± SD (95% CI)	2 ± 1 (2 - 2)		2 ± 1 (2 - 2)	

Figure 1a: Types and mechanisms of injuries for acute referrals

Figure 1b: Types and mechanisms of injuries for operative cases

Figure 2: operative and anaesthetic techniques compared between pre- and post-COVID

For peer review only - http://bmjopen.bmj.com/site/about/guidelines.xhtml

COVID status

At the time, COVID was being diagnosed with polymerase chain reaction (PCR) from nasal and oropharyngeal swabs with a duration of 1 to 4 days where the sample was tested both locally in the hospital lab as well as corroborated with national lab testing to reduce risk of unequivocacy. COVID status for overall patient groups in acute referrals and operative casemix were demonstrated in figure 3, whereas the COVID status of all mortalities were outlined in figure 4.

Figure 3: COVID status for acute referrals and operative cases as a measure of proportions

Figure 4: COVID status of all mortalities (with 5% error bars)

For peer review only

Risk (or prevalence) and odds ratios

Table 3: Risk (or prevalence) and odds ratios for acute referrals and operative caseloads

		Acute referrals			Operative caseload		
		RR	OR	p-value	RR	OR	p-value
Morbidity & Mortality	Mortality	2.50	2.55	0.0005	2.19	2.25	0.004
	Mortality due to COVID-related complications vs non-COVID causes	14.2	19.7	0.004	15.1	22.0	0.004
	Peri-/post-operative complications including COVID				5.88	6.09	0.00001
	Peri-/post-operative complications excluding COVID				3.65	3.72	0.003
	Peri-/post-operative COVID positive testing				32.6	23.4	0.0009
Anaesthetic technique	General anaesthetic only				1.22	1.61	0.00001
	General anaesthetic ± block				1.23	1.75	0.00001
Consultant involvement	Consultant-led operation				1.36	2.08	0.00001
Operation technique	Open reduction + internal fixation				0.81	0.74	0.007
	Dynamic hip screw				2.02	2.11	0.00001
	Removal of metal/foreign body				0.24	0.23	0.003
	Road traffic accident	0.58	0.56	0.001	0.45	0.43	0.00001

For peer review only - http://bmjopen.bmj.com/site/about/guidelines.xhtml

**Mechanism of injury**	Fall (<1.5m)	1.19	1.54	0.00001	1.17	1.49	0.0001
	Sporting injury	0.63	0.60	0.0005	0.64	0.61	0.003
	Infection	0.69	0.66	0.001	1.70	1.77	0.005
	Trauma call				0.55	0.52	0.0005
**Type of injury**	Neck of femur (NOF) fracture	1.44	1.57	0.00001	1.51	1.79	0.00001
	Lower limb (excl. NOF)	0.89	0.84	0.04	0.74	0.65	0.0001
**Gender**	Male	0.91	0.82	0.01			

Table 3 outlined the risk (or prevalence) and prevalence odds ratios alongside their 95% confidence intervals and statistical significance. The risk ratio is synonymous with the prevalence ratio. Only those factors that were statistically significant within the acute referrals and operative caseloads were included.

Although the expectation was to minimize the use of aerosolising-generating anaesthetic procedures, there was in fact an increased prevalence of using general anaesthesia ± block up to an odds of 75%, perhaps to create a ‘closed circuit’ for the airways. As the anaesthetic methods was not well documented in the pre-COVID era in a fifth (21.3%) of cases, this could have skewed the data as it may have been difficult to extract that data from last year. The odds of a Consultant-led operation doubled (OR=2.08) during the COVID period as a consequence of all elective operations being suspended and more Consultants being relocated to trauma theatre and increased pressure within the theatre environments leading to Consultant-delivered, rather than Consultant-led care. With respect to surgical procedures, there was a significant reduction in prevalence ratio of open reduction and internal fixation by a fifth (PR=0.81) and removal of metalwork and foreign bodies by three-quarters

For peer review only

For peer review only - http://bmjopen.bmj.com/site/about/guidelines.xhtml

(PR=0.24), while there was a doubling (PR=2.02) in dynamic hip screw fixation in the COVID era.

With respect to the acute referrals, patients had half (OR=0.52) the odds of presenting as a trauma call. This could be due to the odds ratios of road traffic accidents, sporting injuries, infection, and lower limb injuries were significantly less (by 34-44%; OR=0.56-0.66) during the COVID period. Yet, the odds of presenting with a neck of femur fracture and having falls less than 1.5m height increased by 54% (OR=1.54).

Morbidity and Mortality

Table 3 indicated that the mortality rate more than doubled significantly for both prevalence (or risk; RR=2.19-2.50) and odds (OR=2.25-2.55) ratios during the COVID period. This certainly has both statistical as well as clinical significance. COVID-related complications were still responsible for increasing the odds of mortality by 20 to 22 times within all mortalities from both acute referrals and operative cases (as compared to non-COVID causes for all mortality in the year 2019). Table 4 confirmed that the mean age of mortalities across the board were in the elderly patient population with a high mean ASA grade. Males were consistently in the minority, while neck of femur fracture was the modal diagnosis due to falls and persistently in the majority, followed by lower limb injuries (figures 5a and b). At least 82% of operations were related to neck of femur fractures in which half of all operations during the COVID period involved anaesthetic AGPs. Whereas the mortalities from pre-COVID operations did not have Consultant-led (as primary surgeon) surgery, that increased to three-fifths of all operations conducted during the COVID period (figure 5c). The mean date of presentation to hospital was one week ahead in year 2020 compared to a year ago but the time from admission to mortality differed only by a mean of less than a day in both

For peer review only

categories. Although, COVID positive swabs were confirmed in 29% of acute referrals and in a 32% of operative cases (figure 3), mortalities in each cohort were 6% and 8.1% respectively. However, only 0.9% and 1.6% were confirmed with COVID positive PCR swabs within one week of the date of mortality (figure 4).

For peer review only

Table 4: Patient demographics, date of injuries, and time to mortality

	Acute referrals		***Operative casemix***	
	**2019 (n=23)**	**2020 (n=38)**	**2019 (n=22)**	**2020 (n=34)**
Mortality	1.3%	3.2%	2.2%	4.9%
Mortality with COVID positive PCR result		0.9%		1.6%
Post-op morbidity			0.7%	4.1%
Age (years; mean±SD; 95% CI)	80.2 ± 16.4 (73.2 - 87.2)	77 ± 23 (67 - 88)	83.9±12.2 (78.7 - 89.1)	84.0±13.5 (79.4 - 88.5)
Male	9 39%	16 42%	8 36%	15 44%
ASA (mean±SD; 95% CI)			3 ± 0 (3 to 3)	3 ± 1 (3 to 3)
Date of injury (mean days±SD; 95%CI)	6/4 ± 11 (1/4 - 10/4)	31/3 ± 12 (26/3 - 5/4)	6/4 ±12 (1/4 - 11/4)	30/3 ±14.2 (25/3 - 4/4)
Time from admission to mortality (mean days±SD; 95%CI)	10.3 ± 7.5 (7.1 - 13.5)	11 ± 10 (7 - 15)	14.3 ± 10.4 (9.8 - 18.7)	13.8 ± 10.4 (10.2 - 17.3)

For peer review only

Figure 5a: types and mechanism of injury for mortalities in acute referral cohorts

Figure 5b: types and mechanism of injury for mortalities in operative casemix cohorts

Figure 5c: Surgical and anaesthetic techniques utilized in mortalities as a means of proportions

Taking into account that COVID was a peri-operative complication since patients may have been symptomatic with COVID manifestations pre-operatively but only had the swab results return with a positive finding either pre- or post-operatively; the commonest post-operative complication in the COVID period was a hospital-acquired pneumonia but with negative COVID swab results or the decision not to test at all. The second most common post-operative complication in the year 2020 was extra-pulmonary sepsis (figure 6). The proportion of post-operative complications had significantly increased when including or excluding COVID as a peri- or post-operative complication in 2020 (0.70% vs. 2.57-4.14%; p=0.003) with varying odds (3.72-23.4) and risk (3.65-32.6) ratios (table 3).

Figure 6: Post-operative complications for both years

Figures 7a and b focused on the total number and nature of comorbidities within the mortality groups. Multiple contingency chi-square test was insignificant for both number of comorbidities and individual comorbidities between both years, except for cardiovascular and cerebrovascular disease in acute referrals. This was corroborated by the COVIDSurg publication[14] which confirmed a significant association of mortality with myocardial infarction and congestive heart failure. However, hypertension and stroke/transient ischemic attacks were not significantly associated. In our study, all cardiovascular diseases (including peripheral vascular, arrhythmias, hypertension, heart failure, myocardial infarction and acute coronary syndromes) were combined with cerebrovascular diseases (consisting of strokes and transient ischemic attacks). Unlike their study, our study did not find a significant association with chronic kidney disease, chronic obstructive disease (which included asthma) and dementia in all mortalities during the 2020 timeframe regardless of the COVID status. The differences may stem from that their study looked at the comparison of mortality rates within

For peer review only - http://bmjopen.bmj.com/site/about/guidelines.xhtml

the same cohort during the COVID era, whereas this study is sub-analysing the entire mortality cohort on its own to observe for specific associations and risks.

Figure 7a: Type of comorbidities for all mortalities in both years

Figure 7b: number of comorbidities for all mortalities in both years

Survival probability

A six-week Kaplan-Meier survival probability analysis for mortalities between both years was plotted in figure 8a.

Figure 8a: Six-week Kaplan-Meier survival probability analysis for mortalities between pre- and post-COVID for acutely referred from the Emergency Department

There were similar patterns of survival probability between both cohorts (i.e. 2019 vs 2020 cumulative). However, the lowest survival probability and the shortest timeframe were observed in the confirmed COVID positive cohort as seen in figure 8a. This may be due to the most vulnerable patient profile. 8 (72.7%) patients had femoral trauma, most being neck of femur fractures, distal femur fracture and a dislocated hip hemiarthroplasty post-fracture. Other patients presented with septic arthritis, post-operative complication and knee swelling; yet every patient also suffered from multiple comorbidities including those leading to immunosuppression as seen in figures 7a and b. Although these patients were prioritised in the Emergency Department and recognised for their poor physiological reserve, due to the stresses of the acute and emergency services, these patients may have had to wait longer to be treated acutely and appropriately admitted.

For peer review only - http://bmjopen.bmj.com/site/about/guidelines.xhtml

Figure 8b: Six-week Kaplan-Meier survival probability analysis for mortalities between pre- and post-COVID for those undergoing surgery

Unexpectedly, there was a reversal of trends observed for the six-week Kaplan-Meier survival analysis once admitted and operated on in figure 8b. Mortalities within the pre-COVID period had the lowest survival probability compared to the post-COVID cohort. The COVID positive mortalities were observed to have the highest survival probability 11 days prior to converging with those mortalities without COVID symptoms. This was most likely to be due to multifactorial factors.

During the pandemic, wards were ring-fenced to host confirmed COVID positive patients with a heightened care of nursing, medical cover and personal protective equipment. Prior to the onset of a possible vaccination to counteract the virus, symptomatic management and shielding were the mainstay treatments for COVID positive patients. None of these patients were stepped up to the Intensive Treatment Unit due to being categorised as high-risk stratification for mortality based on age and extent of comorbidities.

If these 'at risk' patients were symptomatic with the virus, then aggressive pre-operative optimisation would occur. Since 91% (n=10) of COVID positive patients had sustained a neck of femur fracture, the National Hip Fracture Database best practice tariff of operating within an ideal 36-hour window set by the Royal College of Physicians was suspended until the patient was stabilised. All hip fracture patients in this cohort were operated on and had dedicated orthogeriatric input commencing from hospital admission. Hence the early peri-operative period and surgery encompassed within the 10-day period post-admission.

For peer review only - http://bmjopen.bmj.com/site/about/guidelines.xhtml

Moreover, neck of femur fractures are recognised as a pre-terminal illness and are known to carry a high risk of mortality in the first month which is trebled in the first year after the injury.[15]

For peer review only

For peer review only - http://bmjopen.bmj.com/site/about/guidelines.xhtml

Discussion

Statement of principal findings

There was a significant difference between pre- and post-COVID periods at its ‘peak weeks’. The alternative hypothesis was not rejected with respect to prevalence of (i) acute orthopaedic trauma referrals (reduced by 34%), (ii) surgical interventions (reduced by 29.5%), (iii) anaesthetic aerosolising-generating procedures, (iv) mortality rates (more than doubled in the COVID period), and (v) survival probability between pre- and post-COVID eras. The 34% reduction in acute trauma referrals is in keeping with previous single centre studies performed in the UK with results ranging between 26-59%.[10-13,16,17] As described in these previous studies we would attribute the overall reduction of trauma workload to be due to reduction in travel and outdoor activities during the national lockdown.

MacDonald *et al.*[18] described a similar effect in their multi-centre study with a reduction of operative workload by 26.5% compared to 29.5% in our study. Sites recruited for this study confirmed that they continued to operate at their own facilities during the data collection period whereas some later used alternative and external facilities including private hospitals through NHS England pathways (as mentioned by Dayananda et al.[19]), which may have impacted nosocomial rates of COVID, morbidity and mortality. However, this would be difficult to assess since it would also depend on the diversions of the ambulance services to ‘clean’ and ‘contaminated’ hospital sites.

There was a significant decline in the odds of trauma calls, road traffic accidents, sporting injuries and lower limb fractures. Conversely, there was a significant rise in the odds of neck of femur fractures, falls, the use of anaesthetic AGP and Consultant-led operations; a finding

For peer review only - http://bmjopen.bmj.com/site/about/guidelines.xhtml

also reflected by Arafa *et al*.[20]. Since the aetiology of neck of femur fracture are often low energy falls in the home environment, it is not unexpected to observe a consistency of neck of femur fractures in the elderly and the vulnerable during lockdown. Odds of falls may have increased due to prodromal symptoms and clinical manifestations of COVID.

Morbidity and Mortality rates

Mortality rates significantly doubled for both prevalence (or risk) and odds ratios during the COVID-19 period and a third (29-32%) of those deaths had a positive COVID-19 diagnosis. Comparatively, the COVIDSurg Collaborative observed a 30-day mortality rate of 28.8% ($p<0.0001$) of Orthopaedic patients who underwent surgery (both elective and trauma) within the first quarter of the year.[14] Those with neck of femur fractures remain at greatest risk of mortality and there have been further studies evaluating the risk of COVID-19 on this inherently high risk cohort.[21-24] The increased mortality reflect the increased proportion of NOFF patients that have a higher baseline mortality which has been echoed by the Scottish IMPACT-Restart study.[24]

A subgroup analysis separating NOFF to non-NOFF mortality is demonstrated in table 4. There was no statistical difference in the odds and risk ratios between both years for mortality rate in NOFF. The numbers have not changed much, but because of a drop off of other cases, the percentage of NOFF markedly rose. Hence, the mortality expressed as a percentage of cases is notably higher for all operations, and not necessarily if stripped down to hip fractures alone.

As lockdown measures in the UK and globally eases and the incidence of trauma returns to pre-lockdown trends, it is imperative that we understand the true increased risk of mortality

For peer review only - http://bmjopen.bmj.com/site/about/guidelines.xhtml

in the acute trauma patient during the COVID-19 era. A recent publication by Kader et al.[25] has suggested that the rate of mortality from COVID-19 for elective Orthopaedic patients is low; yet this is the first nationwide study to quantify mortality risk for trauma patients. Trauma procedures due to the nature of the injuries are necessary and time-critical, and nobody can afford to postpone trauma care even during a global pandemic.[26]

Furthermore, the Corona Hands Collaborative[27] published that upper limb trauma patients had SARS-CoV-2 complication rate of 0.18% (n=2) with 0.09% (n=1) overall mortality at the peak of the first wave in April 2020. However, their collaborative looked into a shorter post-operative period (30 vs 42 days) but they agreed that patients who had been hospitalised for a prolonged period before their surgery were at increased risk of both COVID-related and post-operative complications. Most of their patient cohort, who were both younger and fitter than our cohorts, would be classified as the 'walking wounded' and could usually be day-case procedures.

Although the trends in mechanisms of injury in our study were reflective of those within a US multi-centre study, there was an opposing trend in the number medical/surgical procedures.[28] That could be due to their study encompassing on level 1 trauma centres with a mean younger patient population. However, we do agree that with time and from experiential learning, hospitals improved their coping strategies with the pandemic and enhanced patient safety by enforcing personal protection equipment, hosting dedicated theatres for COVID-positive patients, separating sites as clean and contaminated, ringfencing COVID-positive patients to dedicated wards, and promoting routine COVID PCR swabs for all admissions and pre-operative checklists.

For peer review only - http://bmjopen.bmj.com/site/about/guidelines.xhtml

With an overall mortality risk in 2020 doubled that of 2019, clinicians need to counsel patients presenting with acute orthopaedic trauma of the increased risk in the COVID-19 era, especially for those identified as increased risk stratification with multiple underlying comorbidities, elderly and frailty. With the ongoing risk of a second wave and resurgence of COVID-19 cases on top of the inevitable winter pressures, this data is of critical importance in the risk management, decision-making and policymaking of trauma patients both in the UK and across the globe.

Strengths and weaknesses of the study and in relation to other studies

This was the first representative observational study of the UK looking into the impact of COVID-19 pandemic on general Trauma and Orthopaedic surgical specialty. Studies thus far have only shed light on local scales or cross-speciality.[10-14] Weaknesses included loss of data points which have been accounted for in the tables (i.e. labelled as unknown). However this did not affect the final analysis of data points. Operations conducted outside the specific study periods will not account for all those operations required such as for NOFF. It does not suggest that the number of NOFF not accounted for have been managed conservatively (as discovered by Cherevu et al.[29]), since some NOFFs may breach time to surgery due to medical reasons and allowing for international guidelines.[30]

For peer review only

Limitations and future research

It is vital to continue exploring the impact of the pandemic on a larger scale. Ideally, more secondary care providers consisting of district general hospitals and major trauma centres will submit data. The diagnosis of COVID was dependent on positive PCR swabs for this study rather than non-specific changes seen on chest CT or plain radiographs. This does not account for false negatives with clinical respiratory symptomatology or true positives in those asymptomatic. Nevertheless, this issue with data has been speculated on in another national study.[27] Data ought to be submitted during the peak of the pandemic as well as at various time intervals as the lockdown measures ease resulting in more freedom of movement while also accounting for the continued risk of subsequent waves and national lockdowns.[31] Further studies will also require to compare the impact of the pandemic on the speciality in the UK compared to other countries on other continents.

For peer review only - http://bmjopen.bmj.com/site/about/guidelines.xhtml

Conclusion

This was the first, longest and largest national representation of the impact of COVID-19 pandemic on acute Orthopaedic trauma referrals and mortality between mid-March to end-April, representing the ‘peak weeks’ during the lockdown. The mortality rate for acute referrals, as well as those undergoing operative intervention, more than doubled in odds when compared to the same time interval one year ago. The majority of mortalities consisted of the elderly with neck of femur fractures and cardiovascular and/or cerebrovascular diseases. This study will aid clinicians in counselling trauma patients of the increased risk of mortality during the era of COVID-19 and also aid in both healthcare infrastructure, resource allocation, decision-making and policymaking as we continue to battle with the pandemic.

For peer review only

For peer review only - http://bmjopen.bmj.com/site/about/guidelines.xhtml

References

1. World Health Organization (a). WHO Coronavirus Disease (COVID-19) Dashboard. Available online at https://covid19.who.int. Cited 2021 May 29
2. Wu F, Zhao S, Yu B, Chen YM, Wang W, Song ZG, et al. A new coronavirus associated with human respiratory disease in China. *Nature.* 2020;579:265-9.
3. World Health Organization. Coronavirus disease 2019 (COVID-19): situation report, 66. 26 March 2020. https://www.who.int/docs/default-source/coronaviruse/situation-reports/20200326-sitrep-66-covid-19.pdf?sfvrsn=9e5b8b48_2. Cited 2021 May 30.
4. Spiteri G, Fielding J, Diercke M, Campese C, Enouf V, Gaymard A, et al. First cases of coronavirus disease 2019 (COVID-19) in the WHO European Region, 24 January to 21 February 2020. Eurosurveillance 2020 (25): 2000178.
5. UK Government (a) (2020) Oral statement to Parliament, Controlling the spread of COVID-19: Health Secretary's statement to Parliament. 16 Mar 2020. www.gov.uk/government/speeches/controlling-the-spread-of-covid-19-health-secretarys-statement-to-parliament. Cited 2021 May 25.
6. UK Government (b) (2020) Guidance: Oral statement to and away from others (social distancing). 23 Mar 2020. https://www.gov.uk/government/publications/full-guidance-on-staying-at-home-and-away-from-others. Cited 2020 Sept 5.
7. NHS England (2020a) Next steps on NHS response to COVID-19: Letter from Sir Simon Stevens and Amanda Pritchard. 17 Mar 2020. www.england.nhs.uk/coronavirus/publication/next-steps-on-nhs-response-to-covid-19-letter-from-simon-stevens-and-amanda-pritchard. Cited 2021 May 27.
8. Royal College of Surgeons. Guidance for surgeons working during the COVID-19 pandemic from the Surgical Royal Colleges of the United Kingdom and Ireland. March

For peer review only - http://bmjopen.bmj.com/site/about/guidelines.xhtml

20, 2020. www.rcseng.ac.uk/coronavirus/joint-guidance-for-surgeons-v1. Cited 2021 May 28.

9. British Orthopaedic Association. British Orthopaedic Association for Standards of Trauma (BOAST). Management of patients with urgent orthopaedic conditions and trauma during the coronavirus pandemic. March 24, 2020. www.boa.ac.uk/resources/statement-for-boa-members-on-trauma-and-orthopaedic-care-in-the-uk-during-coronavirus-pandemic.html. Cited 2021 May 28.

10. Park C, Sugand K, Nathwani D, Bhattacharya R, Sarraf KM. Impact of the COVID-19 pandemic on orthopaedic trauma workload in a London level 1 trauma centre: the "golden month". Acta Orthop. 2020 Jun 23;1-6.

11. Murphy T, Akehurst H, Mutimer J. Impact of the 2020 COVID-19 pandemic on the workload of the orthopaedic service in a busy UK district general hospital. Injury 2020;S0020-1383(20)30560-X.

12. Elhalawany A, Beastall J, Cousins G. The impact of the COVID-19 lockdown on orthopaedic emergency presentations in a remote and rural population, Bone Joint Open. 2020;1(10): 621-627.

13. Karia M, Gupta V, Zahra W, et al. The effect of COVID-19 on the trauma burden, theatre efficiency and training opportunities in a district general hospital: planning for a future outbreak, Bone Joint Open. 2020;1-8:494–499.

14. COVIDSurg Collaborative. Mortality and pulmonary complications in patients undergoing surgery with perioperative SARS-CoV-2 infection: an international cohort study. *Lancet*. 2020 May 29.

For peer review only

For peer review only - http://bmjopen.bmj.com/site/about/guidelines.xhtml

15. British Orthopaedic Association. British Orthopaedic Association Standards for Trauma. January 2012. BOAST 1 Version 2 - Patients sustaining a Fragility Hip Fracture. www.boa.ac.uk/uploads/assets/6750e0bf-4aa3-4680-b1612265704512db/patients%20sustaining%20a%20fragility%20hip%20fracture.pdf. Cited 2021 May 25.

16. Atia F, Pocnetz S, Selby A, et al. The effect of the COVID-19 lockdown on hand trauma surgery utilization. *Bone Jt Open.* 2020;1(10):639-643.

17. Esteban PL, Querolt Coll J, Xicola Martínez M, et al. Has COVID-19 affected the number and severity of visits to a traumatology emergency department? *Bone Jt Open.* 2020;1(10):617-620.

18. Dayananda KSS, Mercer ST, Agarwal R, et al. A comparative review of 1,004 orthopaedic trauma patients before and during the COVID-19 pandemic. Bone Jt Open. 2020;1(8):568-575.

19. Dayananda KSS, Mercer ST, Agarwal R, et al. A comparative review of 1,004 orthopaedic trauma patients before and during the COVID-19 pandemic. Bone Jt Open. 2020;1(8):568-575.

20. Arafa M, Nesar S, Abu-Jabeh H, et al. COVID-19 pandemic and hip fractures: impact and lessons learned, Bone Joint Open. 2020;1-9:530–540.

21. Kayani B, Onochie E, Patil V, Begum F, Cuthbert R, Ferguson D, et al. The effects of COVID-19 on perioperative morbidity and mortality in patients with hip fractures. *Bone Joint J.* 2020;1-10.

For peer review only

22. Hall AJ, Clement ND, Farrow L, IMPACT-Scot Study Group, MacLullich AMJ, Dall GF, et al. IMPACT-Scot report on COVID-19 and hip fractures. *Bone Joint J.* 2020;1-10.

23. Clement ND, Hall AJ, Makaram NS, et al. IMPACT-Restart: the influence of COVID-19 on postoperative mortality and risk factors associated with SARS-CoV-2 infection after orthopaedic and trauma surgery. *Bone Joint J.* 2020;102-B(12):1774-1781.

24. Mackay N, Wilding C, Langley C, et al. The impact of COVID-19 on trauma and orthopaedic patients requiring surgery during the peak of the pandemic. *Bone Jt Open.* 2020;1-9:520–529.

25. Kader N, Clement ND, Patel VR, Caplan N, Banaszkiewicz P, Kader D. The theoretical mortality risk of an asymptomatic patient with a negative SARS-CoV-2 test developing COVID-19 following elective orthopaedic surgery. *Bone Joint J.* 2020;1-5.

26. Service BC, Collins AP, Crespo A, Couto P, Gupta S, Avilucea F, et al. Medically necessary orthopaedic surgery during the COVID-19 pandemic: safe surgical practices and a classification to guide treatment. *JBJS.* 2020 Jul 15;102(14):e76.

27. Dean BJF; Corona Hands Collaborative. Mortality and pulmonary complications in patients undergoing upper extremity surgery at the peak of the SARS-CoV-2 pandemic in the UK: a national cohort study. BMJ Qual Saf. 2020:bmjqs-2020-012156.

For peer review only - http://bmjopen.bmj.com/site/about/guidelines.xhtml

28. Salottolo K, Caiafa R, Mueller J, et al. Multicenter study of US trauma centres examining the effect of the COVID-19 pandemic on injury causes, diagnoses and procedures Trauma Surgery & Acute Care Open 2021;6:e000655.

29. Cheruvu MS, Bhachu DS, Mulrain J, et al. Effect of COVID-19 on a rural orthopaedic hip fracture service. Bone Jt Open. 2020;1(8):500-507.

30. Lewis PM, Waddell JP. When is the ideal time to operate on a patient with a fracture of the hip? : a review of the available literature. Bone Joint J. 2016;98-B(12):1573-1581.

31. Adebowale V, Alderson D, Burn W, Dickson J, Godlee F, Goddard A, et al. Covid-19: Call for a rapid forward looking review of the UK's preparedness for a second wave-an open letter to the leaders of all UK political parties [published correction appears in BMJ. 2020 Jul 16;370:m2866. Dixon, Jeanette [corrected to Dickson, Jeanette]]. *BMJ.* 2020;369:m2514. Published 2020 Jun 23.

For peer review only - http://bmjopen.bmj.com/site/about/guidelines.xhtml

Figure 1a: Types and mechanisms of injuries for acute referrals

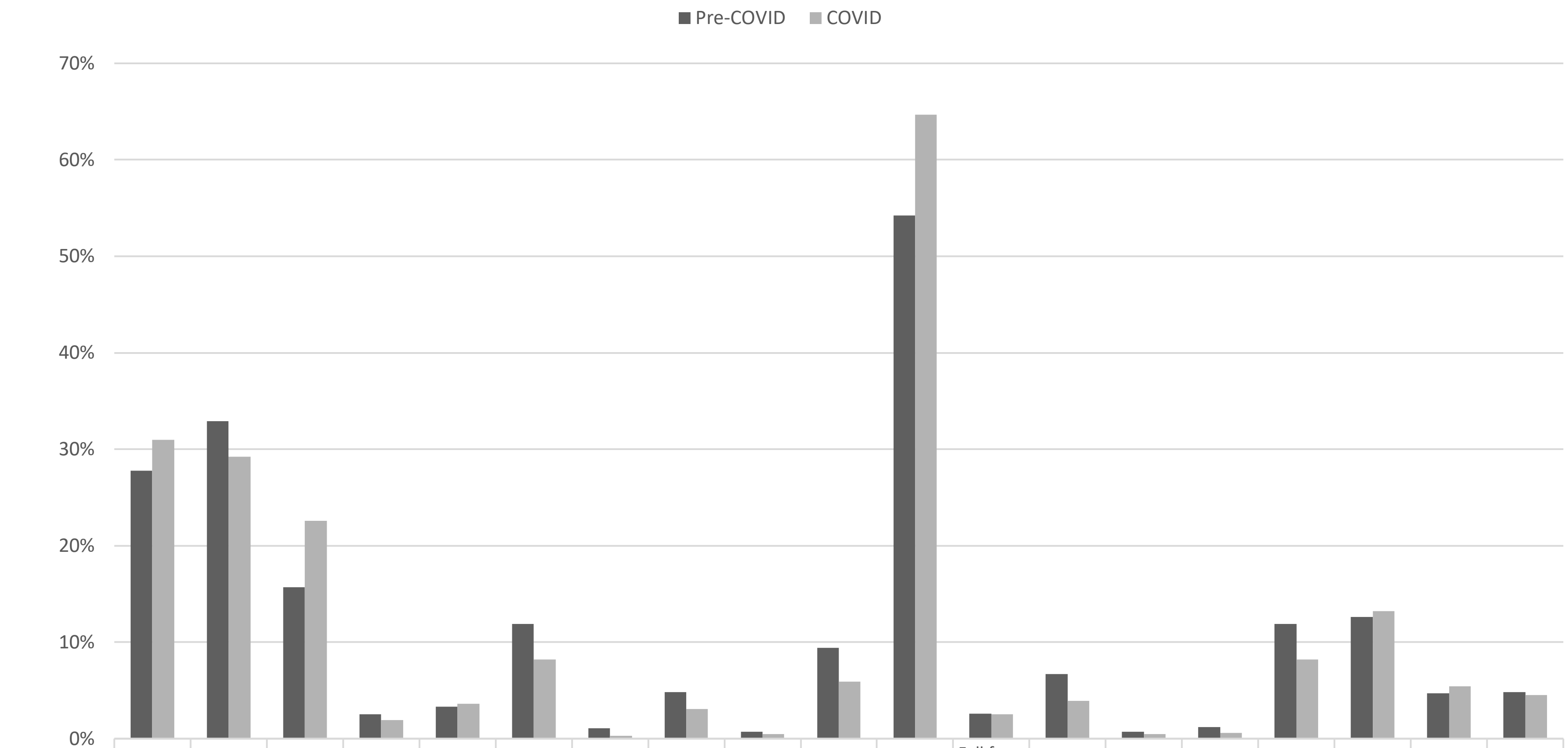

	Upper Limb	Lower Limb	NOF	Pelvis	Polytrauma	Infection	Post-op comps	Other	Assault	Sporting	Fall	Fall from height >1.5m	RTC	Crush injury	Pathological	Infection	Other	Open Injury	Trauma Call
Pre-COVID	27.8%	32.9%	15.7%	2.5%	3.3%	11.9%	1.1%	4.8%	0.7%	9.4%	54.2%	2.6%	6.7%	0.7%	1.2%	11.9%	12.6%	4.7%	4.8%
COVID	31.0%	29.2%	22.6%	1.9%	3.6%	8.2%	0.3%	3.1%	0.5%	5.9%	64.7%	2.5%	3.9%	0.5%	0.6%	8.2%	13.2%	5.4%	4.5%

Type of injuries

Mechanism of injuries

Figure 1b: Types and mechanisms of injuries for operative cases

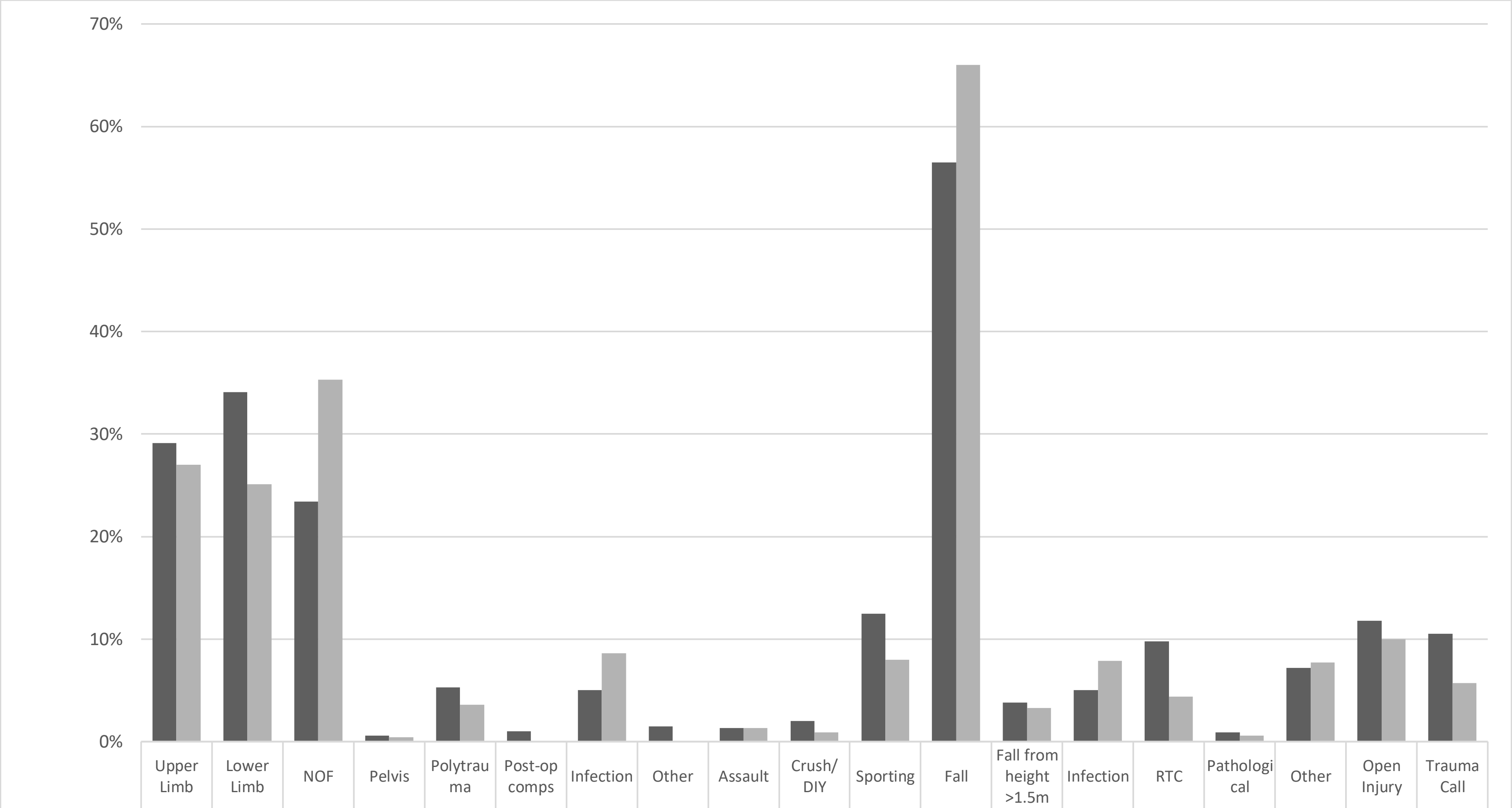

	Upper Limb	Lower Limb	NOF	Pelvis	Polytrauma	Post-op comps	Infection	Other	Assault	Crush/ DIY	Sporting	Fall	Fall from height >1.5m	Infection	RTC	Pathological	Other	Open Injury	Trauma Call
Pre-COVID	29.1%	34.1%	23.4%	0.6%	5.3%	1.0%	5.0%	1.5%	1.3%	2.0%	12.5%	56.5%	3.8%	5.0%	9.8%	0.9%	7.2%	11.8%	10.5%
COVID	27.0%	25.1%	35.3%	0.4%	3.6%	0.0%	8.6%	0.0%	1.3%	0.9%	8.0%	66.0%	3.3%	7.9%	4.4%	0.6%	7.7%	10.0%	5.7%

Type of injuries

Mechanism of injuries

Figure 2: operative and anaesthetic techniques compared between pre- and post-COVID

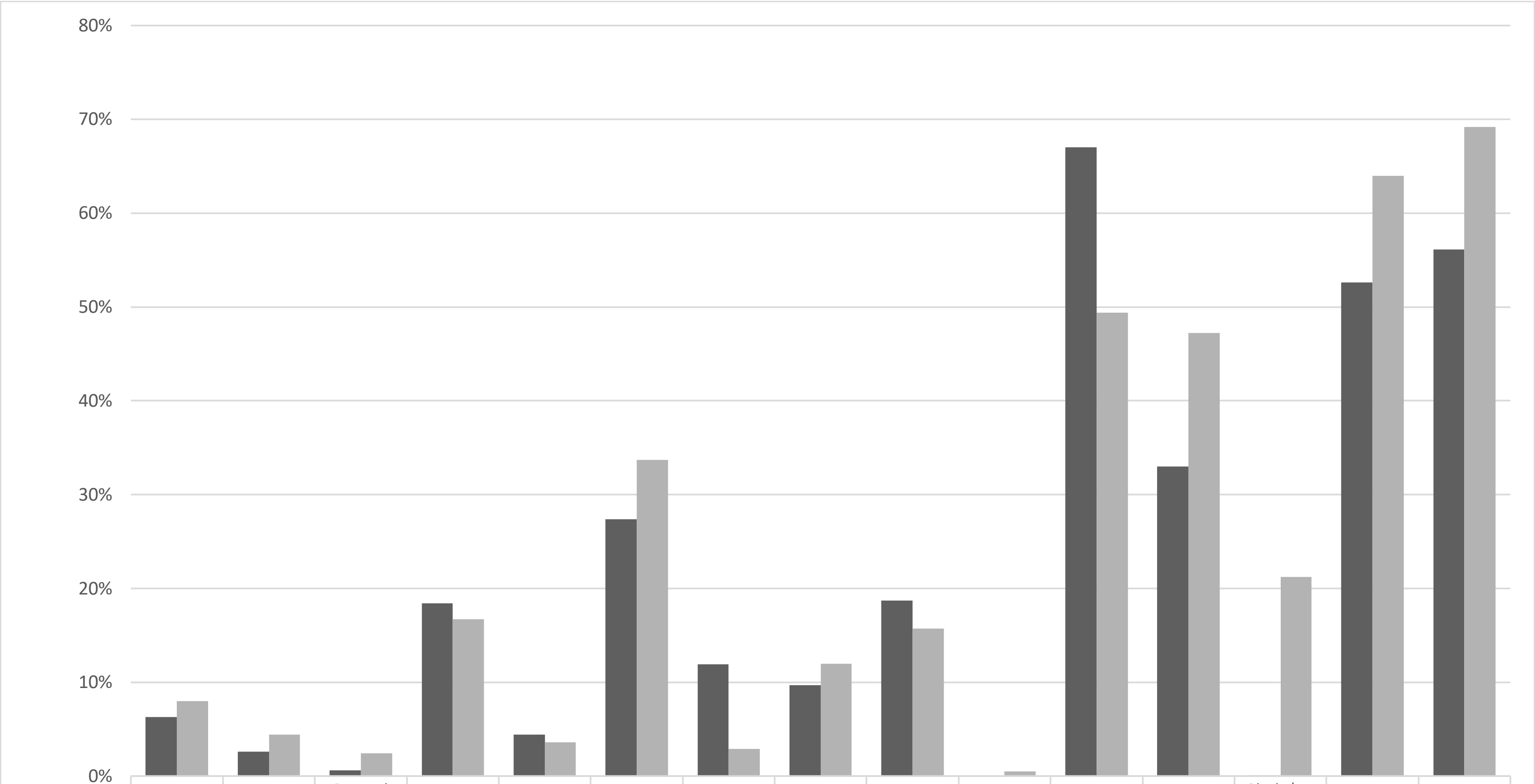

	MUA	ExFix	Removal metal/ FB	ST/Other	K-wire/CRIF	ORIF	DHS	IMN	Arthroplasty	Other	Consultant	Trainee	Blanks/ no document	GA only	GA +/- block
■ COVID	6.3%	2.6%	0.6%	18.4%	4.4%	27.4%	11.9%	9.7%	18.7%	0.0%	67.0%	33.0%	0.1%	52.6%	56.1%
■ Pre-COVID	8.0%	4.4%	2.4%	16.7%	3.6%	33.7%	2.9%	12.0%	15.7%	0.5%	49.4%	47.2%	21.2%	64.0%	69.2%

S

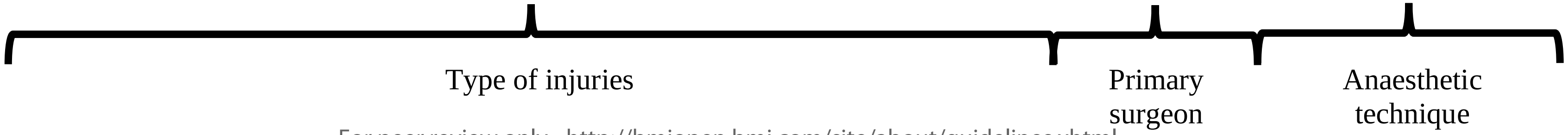

Figure 3: COVID status for acute referrals and operative cases as a measure of proportions (with 5% error bars)

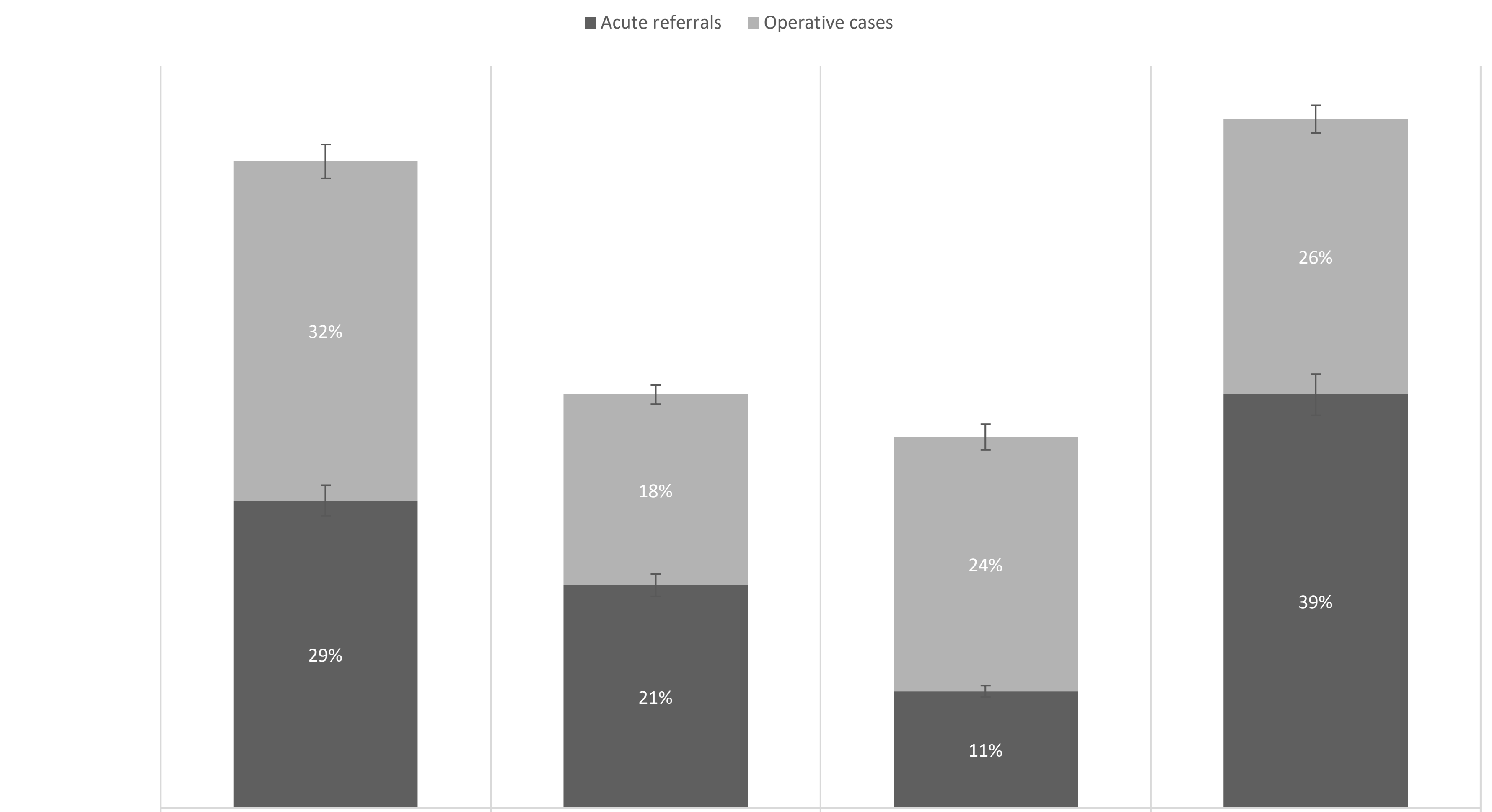

	Positive	Negative	Unknown	Not tested/asymptomatic
Operative cases	32%	18%	24%	26%
Acute referrals	29%	21%	11%	39%

Figure 4: COVID status of all mortalities (with 5% error bars)

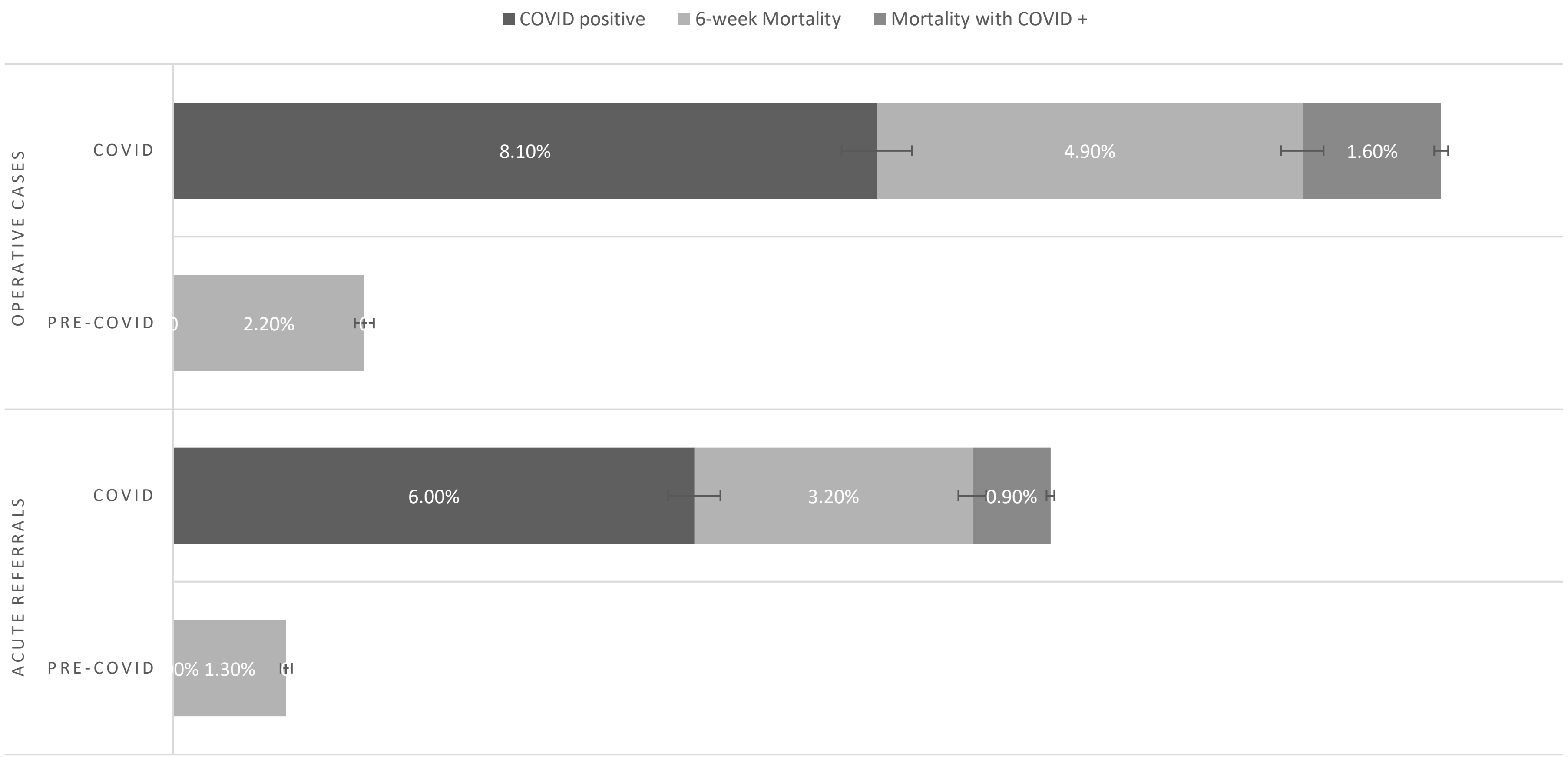

	Acute referrals		Operative cases	
	Pre-COVID	COVID	Pre-COVID	COVID
COVID positive	0.00%	6.00%	0	8.10%
6-week Mortality	1.30%	3.20%	2.20%	4.90%
Mortality with COVID +	0	0.90%	0	1.60%

Figure 5a: types and mechanism of injury for mortalities in acute referral cohorts

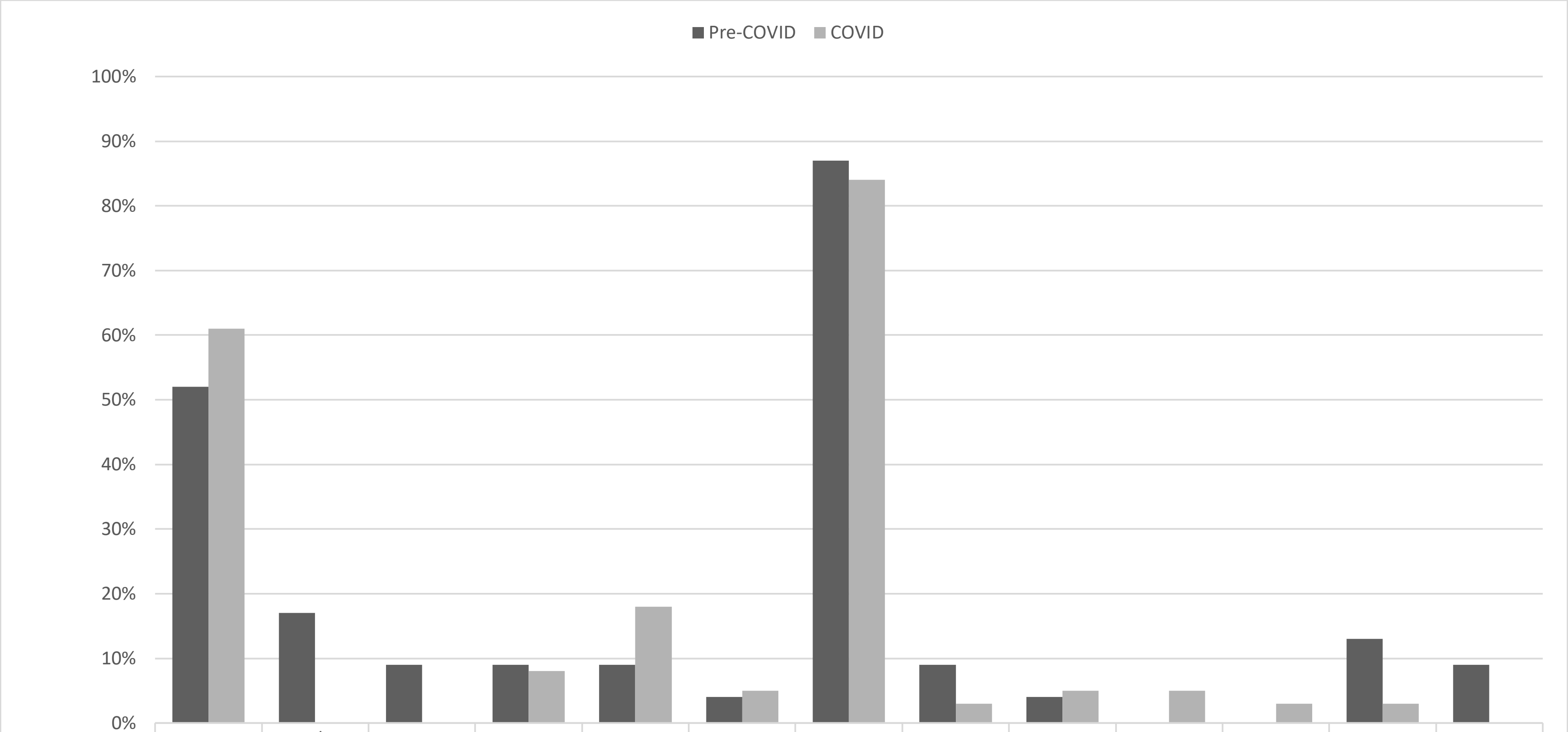

	NOF	Poly-trauma	Pelvis	UL	LL	Other	Falls	RTA	Other	Infection	Sporting	Open injury	Trauma call
Pre-COVID	52%	17%	9%	9%	9%	4%	87%	9%	4%	0%	0%	13%	9%
COVID	61%	0%	0%	8%	18%	5%	84%	3%	5%	5%	3%	3%	0%

***Key**: UL: upper limb / LL: lower limb / RTA: road traffic accidents*

Figure 5b: types and mechanism of injury for mortalities in operative casemix cohorts

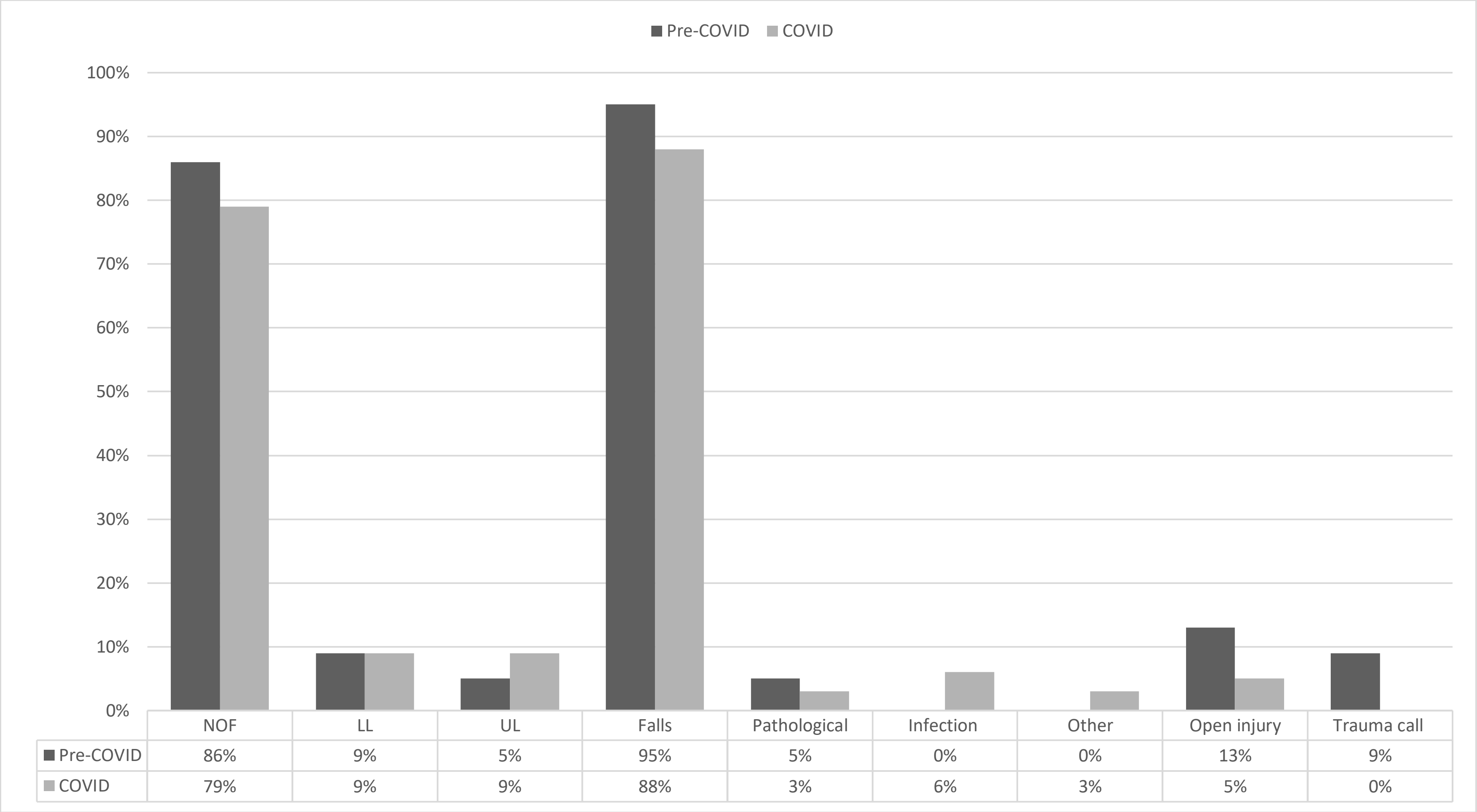

	NOF	LL	UL	Falls	Pathological	Infection	Other	Open injury	Trauma call
Pre-COVID	86%	9%	5%	95%	5%	0%	0%	13%	9%
COVID	79%	9%	9%	88%	3%	6%	3%	5%	0%

***Key**: NOF: neck of femur fracture / UL: upper limb / LL: lower limb*

Figure 5c: Surgical and anaesthetic techniques utilized in mortalities as a means of proportions

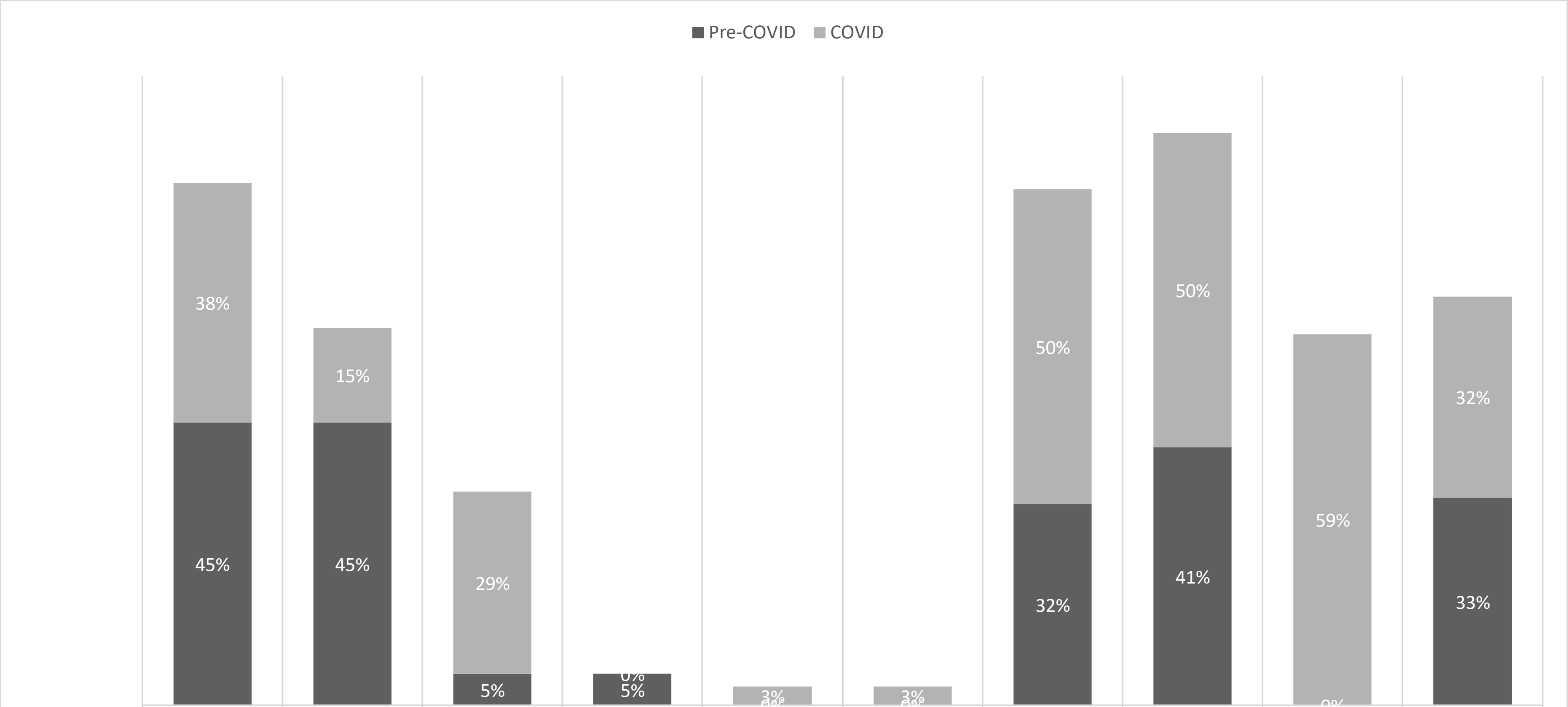

	Hip hemiarthroplasty	IMN	DHS	MUA	Soft tissue	Ex-Fix	AGP	Unknown anesthetic	Consultant-led	Post-op complications
COVID	38%	15%	29%	0%	3%	3%	50%	50%	59%	32%
Pre-COVID	45%	45%	5%	5%	0%	0%	32%	41%	0%	33%

***Key**: IMN: intramedullary nailing / DHS: dynamic hip screw / MUA: manipulation under anaesthesia / Ex-Fix: external fixation / AGP: aerosolizing-generating procedures*

Figure 6: Post-operative complications for both years

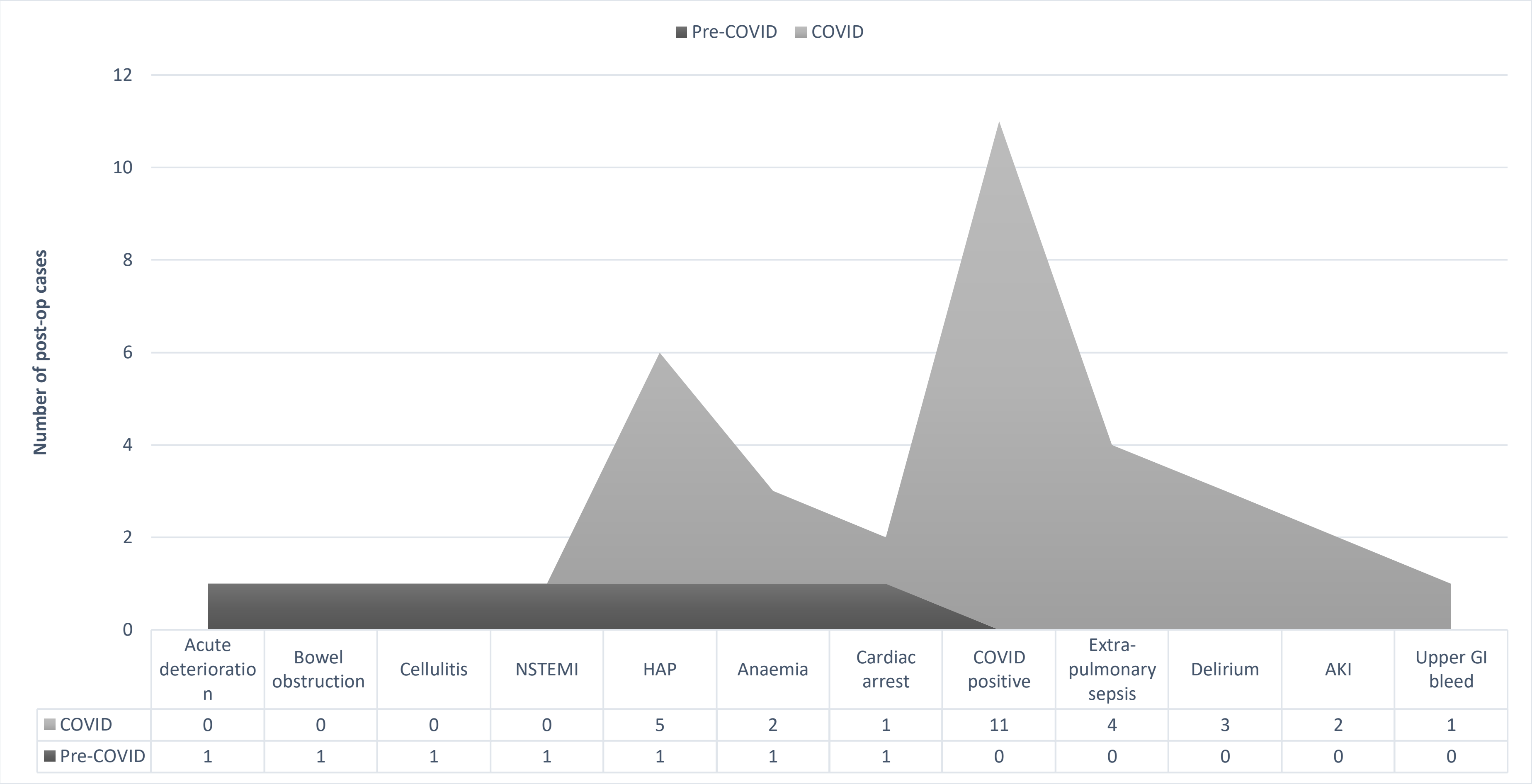

	Acute deterioration	Bowel obstruction	Cellulitis	NSTEMI	HAP	Anaemia	Cardiac arrest	COVID positive	Extra-pulmonary sepsis	Delirium	AKI	Upper GI bleed
COVID	0	0	0	0	5	2	1	11	4	3	2	1
Pre-COVID	1	1	1	1	1	1	1	0	0	0	0	0

***Key**: NSTEMI: non-ST elevated myocardial infarction, HAP: hospital-acquired pneumonia, AKI: acute kidney injury, GI: gastrointestinal*

Figure 7a: Type of comorbidities for all mortalities in both years

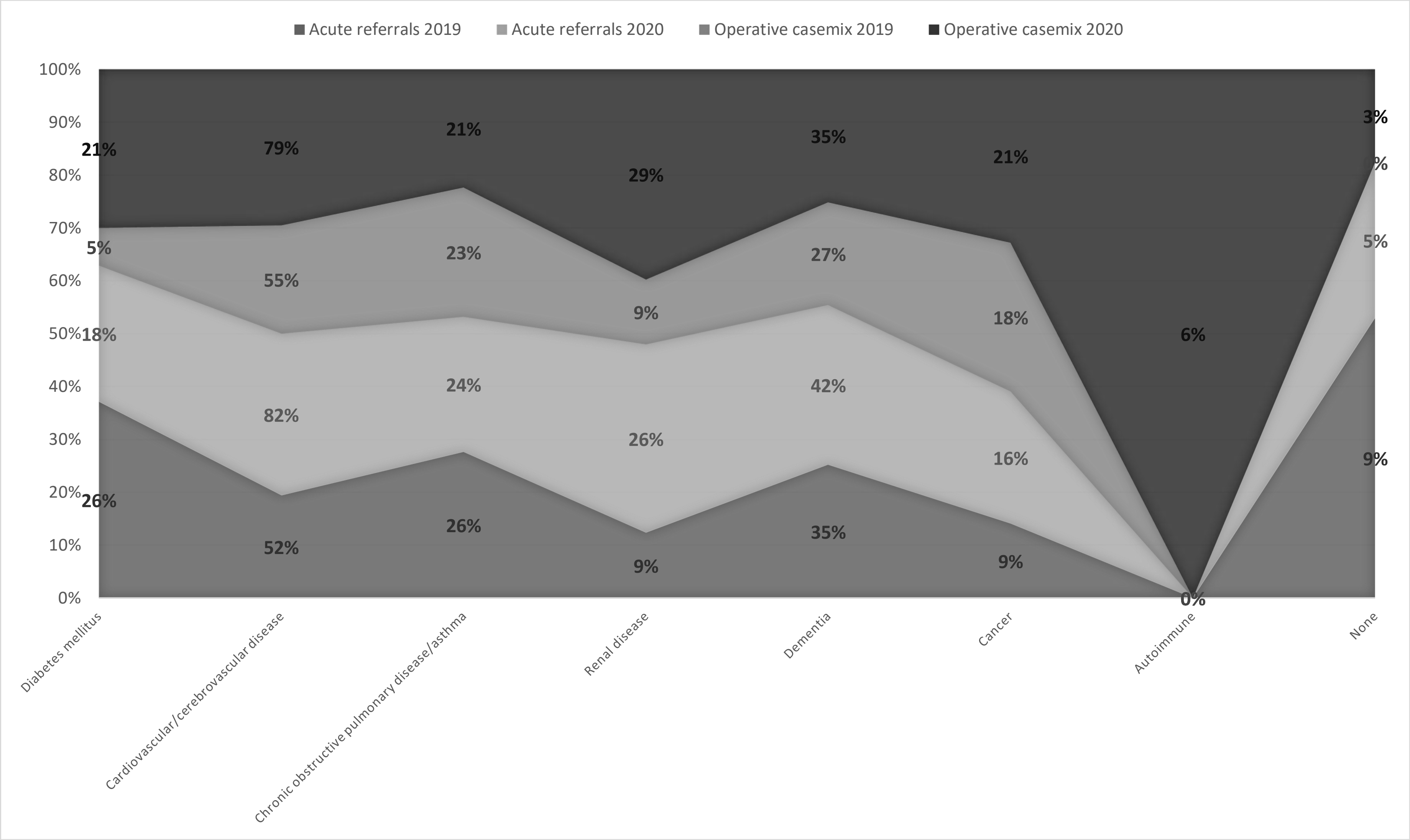

Figure 7b: number of comorbidities for all mortalities in both years

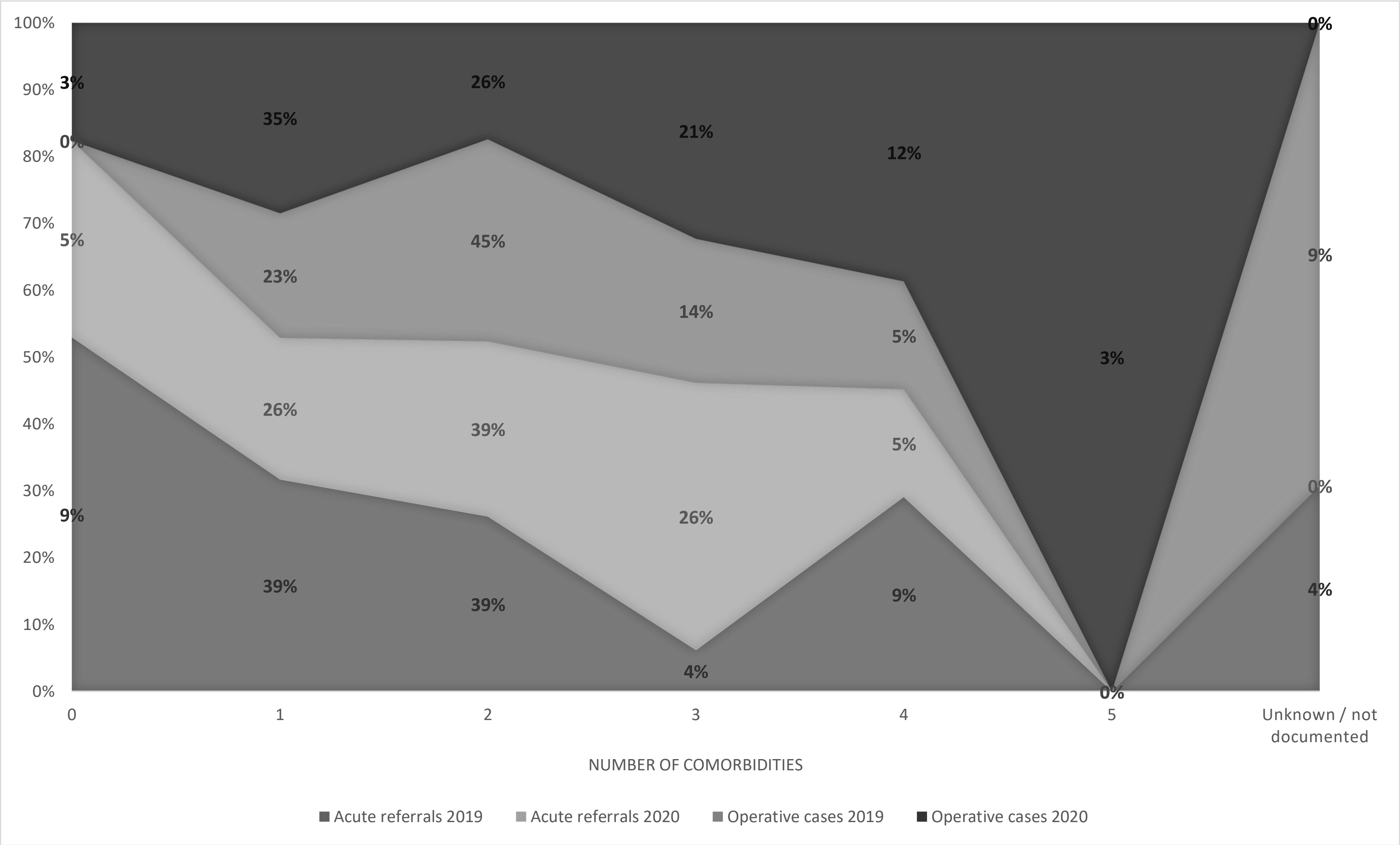

Figure 8a: Six-week Kaplan-Meier survival probability analysis for mortalities between pre- and post-COVID for acutely referred from the Emergency Department

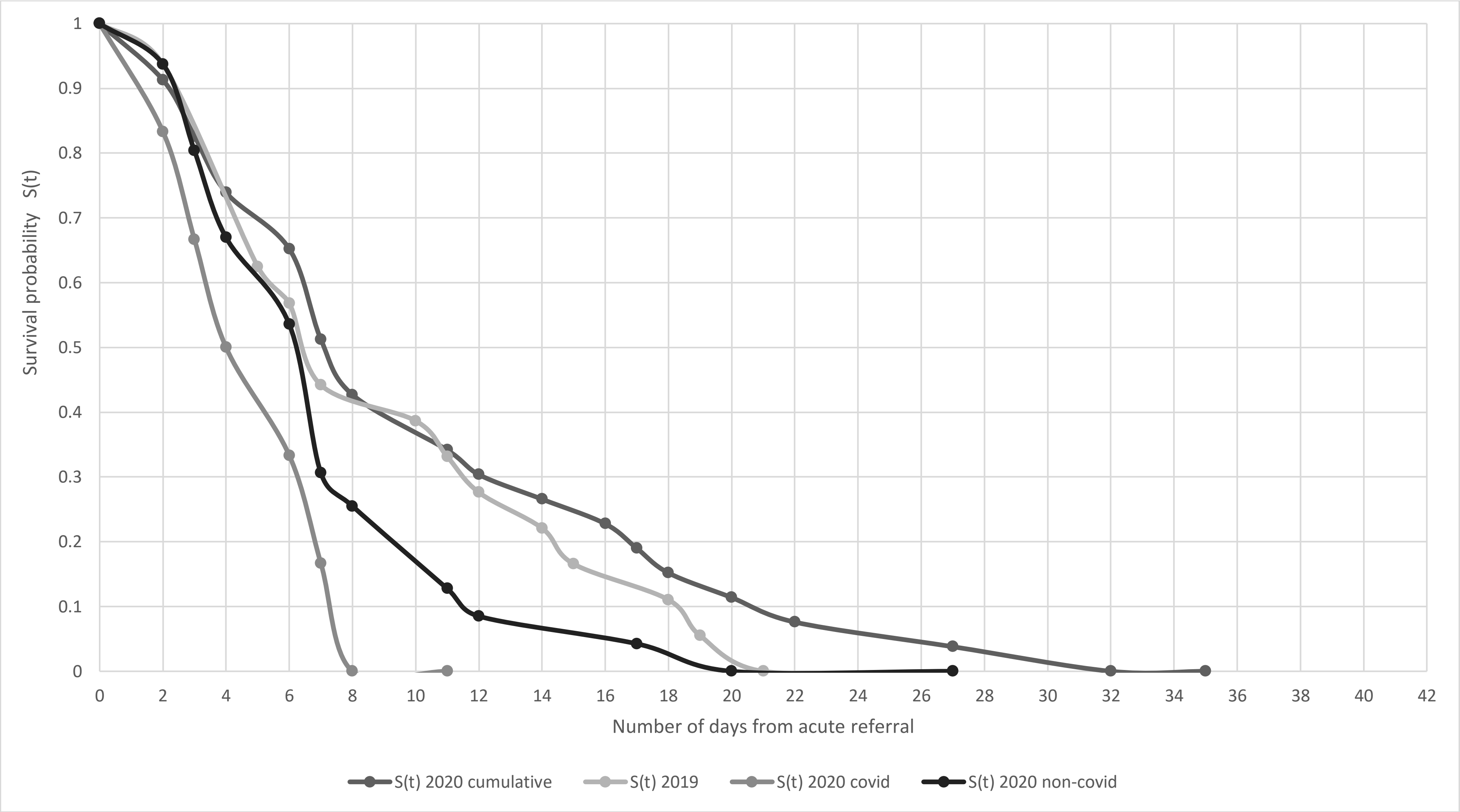

Figure 8b: Six-week Kaplan-Meier survival probability analysis for mortalities between pre- and post-COVID for those undergoing surgery

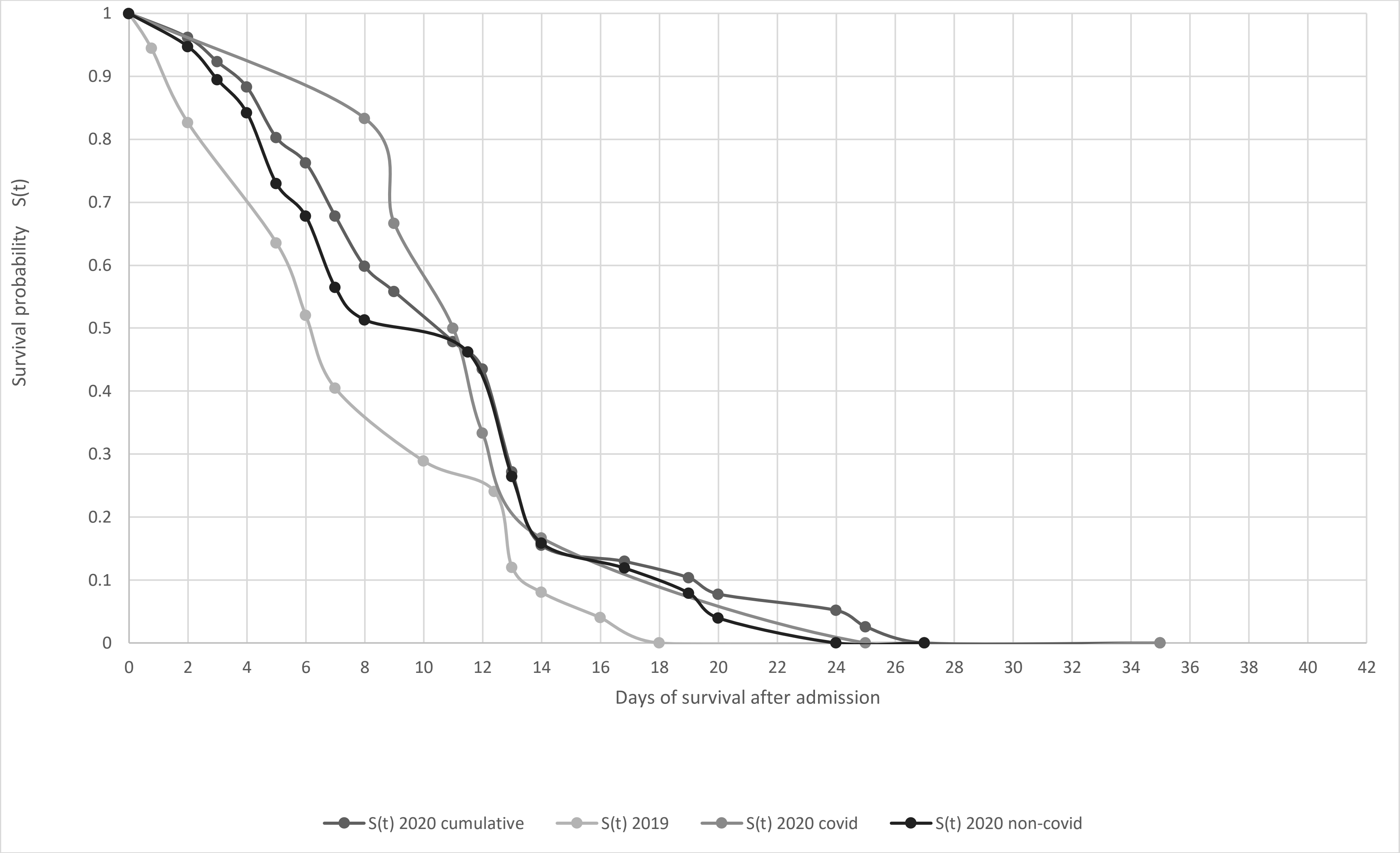

STROBE Statement—checklist of items that should be included in reports of observational studies

	Item No	Recommendation
**Title and abstract**	1	(*a*) Indicate the study's design with a commonly used term in the title or the abstract
		(*b*) Provide in the abstract an informative and balanced summary of what was done and what was found
**Introduction**		
Background/rationale	2	Explain the scientific background and rationale for the investigation being reported
Objectives	3	State specific objectives, including any prespecified hypotheses
**Methods**		
Study design	4	Present key elements of study design early in the paper
Setting	5	Describe the setting, locations, and relevant dates, including periods of recruitment, exposure, follow-up, and data collection
Participants	6	(*a*) *Cohort study*—Give the eligibility criteria, and the sources and methods of selection of participants. Describe methods of follow-up<br>*Case-control study*—Give the eligibility criteria, and the sources and methods of case ascertainment and control selection. Give the rationale for the choice of cases and controls<br>*Cross-sectional study*—Give the eligibility criteria, and the sources and methods of selection of participants
		(*b*) *Cohort study*—For matched studies, give matching criteria and number of exposed and unexposed<br>*Case-control study*—For matched studies, give matching criteria and the number of controls per case
Variables	7	Clearly define all outcomes, exposures, predictors, potential confounders, and effect modifiers. Give diagnostic criteria, if applicable
Data sources/ measurement	8*	For each variable of interest, give sources of data and details of methods of assessment (measurement). Describe comparability of assessment methods if there is more than one group
Bias	9	Describe any efforts to address potential sources of bias
Study size	10	Explain how the study size was arrived at
Quantitative variables	11	Explain how quantitative variables were handled in the analyses. If applicable, describe which groupings were chosen and why
Statistical methods	12	(*a*) Describe all statistical methods, including those used to control for confounding
		(*b*) Describe any methods used to examine subgroups and interactions
		(*c*) Explain how missing data were addressed
		(*d*) *Cohort study*—If applicable, explain how loss to follow-up was addressed<br>*Case-control study*—If applicable, explain how matching of cases and controls was addressed<br>*Cross-sectional study*—If applicable, describe analytical methods taking account of sampling strategy
		(*e*) Describe any sensitivity analyses

Continued on next page

For peer review only - http://bmjopen.bmj.com/site/about/guidelines.xhtml

Section/Topic	Item #	Recommendation
**Results**		
Participants	13*	(a) Report numbers of individuals at each stage of study—eg numbers potentially eligible, examined for eligibility, confirmed eligible, included in the study, completing follow-up, and analysed
		(b) Give reasons for non-participation at each stage
		(c) Consider use of a flow diagram
Descriptive data	14*	(a) Give characteristics of study participants (eg demographic, clinical, social) and information on exposures and potential confounders
		(b) Indicate number of participants with missing data for each variable of interest
		(c) *Cohort study*—Summarise follow-up time (eg, average and total amount)
Outcome data	15*	*Cohort study*—Report numbers of outcome events or summary measures over time
		Case-control study—Report numbers in each exposure category, or summary measures of exposure
		Cross-sectional study—Report numbers of outcome events or summary measures
Main results	16	(*a*) Give unadjusted estimates and, if applicable, confounder-adjusted estimates and their precision (eg, 95% confidence interval). Make clear which confounders were adjusted for and why they were included
		(*b*) Report category boundaries when continuous variables were categorized
		(*c*) If relevant, consider translating estimates of relative risk into absolute risk for a meaningful time period
Other analyses	17	Report other analyses done—eg analyses of subgroups and interactions, and sensitivity analyses
**Discussion**		
Key results	18	Summarise key results with reference to study objectives
Limitations	19	Discuss limitations of the study, taking into account sources of potential bias or imprecision. Discuss both direction and magnitude of any potential bias
Interpretation	20	Give a cautious overall interpretation of results considering objectives, limitations, multiplicity of analyses, results from similar studies, and other relevant evidence
Generalisability	21	Discuss the generalisability (external validity) of the study results
**Other information**		
Funding	22	Give the source of funding and the role of the funders for the present study and, if applicable, for the original study on which the present article is based

*Give information separately for cases and controls in case-control studies and, if applicable, for exposed and unexposed groups in cohort and cross-sectional studies.

Note: An Explanation and Elaboration article discusses each checklist item and gives methodological background and published examples of transparent reporting. The STROBE checklist is best used in conjunction with this article (freely available on the Web sites of PLoS Medicine at http://www.plosmedicine.org/, Annals of Internal Medicine at http://www.annals.org/, and Epidemiology at http://www.epidem.com/). Information on the STROBE Initiative is available at www.strobe-statement.org.

For peer review only

For peer review only - http://bmjopen.bmj.com/site/about/guidelines.xhtml

BMJ Open

The impact of COVID-19 on acute trauma and orthopaedic referrals and surgery in the UK during the first wave of the pandemic: a multicentre observational study

Journal:	*BMJ Open*
Manuscript ID	bmjopen-2021-054919.R1
Article Type:	Original research
Date Submitted by the Author:	12-Oct-2021
Complete List of Authors:	Sugand, Kapil; Imperial College London, MSk Lab<br>Aframian, Arash; Imperial College London, MSk Lab<br>Park, Chang; Imperial College London, Trauma and orthopaedics<br>Sarraf , Khaled; Imperial College London, Trauma and Orthopaedics<br>Collaborative, COVERT; Imperial College London
**Primary Subject Heading**:	Surgery
Secondary Subject Heading:	Emergency medicine, Epidemiology, Surgery, Medical management, Health services research
Keywords:	COVID-19, TRAUMA MANAGEMENT, ORTHOPAEDIC & TRAUMA SURGERY, EPIDEMIOLOGY

SCHOLARONE™
Manuscripts

I, the Submitting Author has the right to grant and does grant on behalf of all authors of the Work (as defined in the below author licence), an exclusive licence and/or a non-exclusive licence for contributions from authors who are: i) UK Crown employees; ii) where BMJ has agreed a CC-BY licence shall apply, and/or iii) in accordance with the terms applicable for US Federal Government officers or employees acting as part of their official duties; on a worldwide, perpetual, irrevocable, royalty-free basis to BMJ Publishing Group Ltd ("BMJ") its licensees and where the relevant Journal is co-owned by BMJ to the co-owners of the Journal, to publish the Work in this journal and any other BMJ products and to exploit all rights, as set out in our licence.

The Submitting Author accepts and understands that any supply made under these terms is made by BMJ to the Submitting Author unless you are acting as an employee on behalf of your employer or a postgraduate student of an affiliated institution which is paying any applicable article publishing charge ("APC") for Open Access articles. Where the Submitting Author wishes to make the Work available on an Open Access basis (and intends to pay the relevant APC), the terms of reuse of such Open Access shall be governed by a Creative Commons licence – details of these licences and which Creative Commons licence will apply to this Work are set out in our licence referred to above.

Other than as permitted in any relevant BMJ Author's Self Archiving Policies, I confirm this Work has not been accepted for publication elsewhere, is not being considered for publication elsewhere and does not duplicate material already published. I confirm all authors consent to publication of this Work and authorise the granting of this licence.

For peer review only

The impact of COVID-19 on acute trauma and orthopaedic referrals and surgery in the UK during the first wave of the pandemic: a multicentre observational study

The COVid-Emergency Related Trauma and orthopaedics (COVERT) Collaborative

Kapil Sugand, Arash Aframian, Chang Park, Khaled M Sarraf, COVERT Collaborative

Department of Trauma and Orthopaedics, Imperial College Healthcare NHS Trust, London, UK

Corresponding author:

Kapil Sugand

Dept T&O, St Mary's Hospital, Praed St, London, W2 1NY

E: ks704@ic.ac.uk

T: 07773642813

Keywords: COVID-19; orthopaedic trauma; UK multi-centre; pandemic wave; mortality

For peer review only - http://bmjopen.bmj.com/site/about/guidelines.xhtml

Word count: 4000 words

For peer review only

For peer review only - http://bmjopen.bmj.com/site/about/guidelines.xhtml

Abstract

Objective: This is the first British multi-centre study observing the impact of the COVID-19 pandemic on orthopaedic trauma with respect to referrals, operative caseload and mortality during its peak.

Design: A longitudinal, multi-centre, retrospective, observational, cohort study was conducted during the peak 6 weeks of the first wave from March 17, 2020 compared to the same period in 2019.

Setting: Hospitals from six major urban cities were recruited around the UK, including London.

Participants: A total of 4840 clinical encounters were initially recorded. 4668 clinical encounters were analysed post-exclusion.

Primary and secondary outcome measures: Primary outcomes included the number of acute trauma referrals and those undergoing operative intervention, mortality rates, and the proportion of patients contracting COVID-19. Secondary outcomes consisted of the mechanism of injury, type of operative intervention and proportion of aerosolising-generating anaesthesia utilised.

Results: During the COVID-19 period there was a 34% reduction in acute orthopaedic trauma referrals compared to 2019 (1792 down to 1183 referrals), and 29.5% less surgical interventions (993 down to 700 operations). The mortality rate significantly (both statistically and clinically) more than doubled for both risk and odds ratios during the COVID period in all referrals (1.3% vs 3.8%, p=0.0005) and in those undergoing operative intervention (2.2% vs 4.9%, p=0.004). Moreover, mortality due to COVID-related complications (versus non-COVID causes) had greater odds by a factor of at least 20 times. For the operative cohort during COVID, there was a greater odds of aerosolising-generating anaesthesia (including

For peer review only

those with superimposed regional blocks) by three-quarters as well as doubled odds of a Consultant acting as the primary surgeon.

Conclusion: Although there was a reduction of acute trauma referrals and those undergoing operative intervention, the mortality rate still more than doubled in odds during the peak of the pandemic compared to the same time interval one year ago.

Keywords: COVID-19; orthopaedic trauma; UK multi-centre; pandemic wave; mortality

Article summary: Strengths and limitations of this study

- This was the first representative observational study of the UK looking into the impact of COVID-19 pandemic on general trauma and orthopaedic surgical specialty.
- There is a valid comparison between two timeframes, exactly one year apart to represent pre-COVID and during COVID.
- Other studies thus far have only shed light on local scales or cross-speciality within a shorter timeframe than this study and not necessarily commenting on mortality rates like this study.
- Weaknesses included loss of data points which have been accounted for in the tables (i.e. labelled as unknown) which did not affect the final analysis of data points.

For peer review only

For peer review only - http://bmjopen.bmj.com/site/about/guidelines.xhtml

- Operations conducted outside the specific study periods will not account for all those operations required such as for hip fractures.

For peer review only

For peer review only - http://bmjopen.bmj.com/site/about/guidelines.xhtml

Introduction

The Global Impact of COVID-19

The COVID-19 pandemic will be remembered as one of the most unprecedented global health crises in modern history. With over 4.5 million deaths and over 224 million recorded cases globally, the pandemic has had a permanent impact on healthcare at the time of article submission.[1] The viral outbreak was first reported in December 2019 with the first patient hospitalised in the city of Wuhan, China.[2] By mid-March the outbreak affected over 190 countries with over 450,000 cases and over 20,000 deaths, thus being declared a pandemic and a global public health emergency by the World Health Organization.[3] On January 24th 2020 Europe reported its first case followed by a case in the United Kingdom (UK) 5 days later.[4]

The British Response to the pandemic

The English government responded by implementing social distancing measures on the 17th March 2020 in an attempt to reduce the rate of transmission and therefore the demands on the National Health Service (NHS).[5] This was followed a week later by more stringent measures, commonly referred to as a societal 'lockdown'.[6] As of the 23rd March 2020, all members of the public were required to stay at home. The NHS has also been deeply affected by the strain imposed by the virus as the healthcare infrastructure has had to evolve to cope with the overwhelming and unexpected pressures on staff, resources and finances. There has been a complete renovation of emergency medicine and orthopaedic services to manage musculoskeletal disease and trauma. In response to the NHS emergency declaration,[7] the Royal Colleges of Surgeons[8] and the British Orthopaedic Association[9] both issued statements and guidelines for delivering emergency trauma and orthopaedic care during the COVID-19 outbreak. The lockdown to limit the spread of the virus has had an unforeseen effect in

For peer review only - http://bmjopen.bmj.com/site/about/guidelines.xhtml

significantly reducing the acute trauma workload described in several single centre studies.[10-13] There has however not been a British multi-centre reflection of the impact of the COVID-19 pandemic on the orthopaedic workload and its potential impact on the mortality.

Aim

To observe the impact of COVID-19 on trauma and orthopaedic acute referrals, operative casemix and mortality rates during the peak 6 weeks of the first wave of the pandemic compared to the same time interval in 2019.

Alternative hypothesis

When comparing both years, there would be a difference in the prevalence of acute orthopaedic referrals, orthopaedic trauma casemix and aerosol-generating anaesthetic procedures due to social distancing/lockdown. Mortality rates and survival probabilities were also hypothesised to differ due to the first COVID-19 outbreak.

For peer review only - http://bmjopen.bmj.com/site/about/guidelines.xhtml

Methods

Study design: This is the first multi-centre longitudinal observational study observing patients who were acutely referred to the trauma and orthopaedic departments as well as those operated on within the same 6-week interval comparing 2019 to 2020.

Setting: 7 principal hospitals contributed data from 6 major urban cities including London, Gateshead, Middlesbrough, Dartford, Newport, and Reading.

Patient sampling: All acute referrals, operative notes, inpatient medical records and discharge summaries were accessed using electronic medical system at each contributing hospital trust.

Study period: The 6-week study period was from the start of social distancing on Tuesday 17th March 2020 to Tuesday 28th April 2020 which encompassed the national lockdown measures instigated on the 23rd March 2020. This period was considered the peak 6 weeks of the epidemic in the UK as outlined by the recorded mortality rates and R-values published by the Office of National Statistics.[14] This time period was compared to the same 6-week interval from Tuesday 19th March to Tuesday 30th April 2019 (i.e. prior to any COVID-19 related measures) to compare the impact of the pandemic one year apart.

Outcomes/objectives: Primary outcomes included the number of acute trauma referrals and those undergoing operative intervention, post-operative complications, mortality rates, and the proportion of patients contracting COVID-19. Secondary outcomes consisted of the

For peer review only - http://bmjopen.bmj.com/site/about/guidelines.xhtml

129 mechanism of injury, type of operative intervention and proportion of aerosolising-generating
130 anaesthesia utilised.
131
132 *Inclusion criteria:* All acute orthopaedic trauma referrals presenting to the Emergency
133 Department during the intervals one year apart were included. All orthopaedic trauma cases
134 that required an operation, including those from acute orthopaedic trauma referrals, within
135 the intervals one year apart. Those patients listed for an operation due to orthopaedic trauma
136 prior to time period of data collection were included in the final analysis. We adhered to
137 STROBE guidelines for observational studies.
138
139 *Exclusion criteria:* Any cases being referred internally from other specialties for trauma and
140 orthopaedic advice and input, as well as referrals from any external centre asking for tertiary
141 advice were excluded from further analysis. Any patients with post-operative complications
142 arising from the period prior to the data collection were excluded. For operative trauma cases,
143 those undergoing spinal procedures were excluded as these are jointly treated by
144 Neurosurgery in most hospitals. All non-urgent semi-elective procedures were excluded from
145 analysis as well, as they would inaccurately assess the impact of any social distancing
146 measures on the trauma workload. Routine elective orthopaedic cases were excluded.
147
148 *Data points:* Demographics including age, sex and ASA grades were recorded for all
149 patients. Injury characteristics were recorded, including the anatomical location and if the
150 injury was open or closed. The mechanism of injury was categorised and whether the patient
151 was referred as a trauma call. The nature of the operative procedures and the anaesthetic
152 techniques were recorded. Patients undergoing multiple procedures were recorded for every

For peer review only - http://bmjopen.bmj.com/site/about/guidelines.xhtml
For peer review only

episode where they were taken to theatre. 6-week mortality rate was recorded as well as the COVID-19 status of any symptomatic patients or suspected cases. Data points were divided into acute referrals and operative casemix as seen in table 1.

Table 1: data points for acute referrals and operative casemix

Age (years)	Gender (Male / female by birth)	ASA (1-5)	Date of injury / presentation
Injury	Mechanism of injury	Open Vs Closed fracture	Trauma Call (Yes / No)
Operative procedure	Anaesthetic Technique (AGP vs non-AGP)	Seniority of Surgeon (Consultants vs trainees)	Comorbidities
Six-week mortality	Post-op complications	Surgery time since admission (hours)	COVID status (from PCR swabs)

- *Anaesthetic techniques:* This was divided into anaesthetic aerosolising-generating procedures (AGP) which consisted of any intubation (including laryngeal mask airway and endotracheal intubation) for a general anaesthetic. All other anaesthetic techniques including regional and local anaesthetics were deemed as non-AGPs.
- *COVID status*: At the time, COVID was being diagnosed with polymerase chain reaction (PCR) from nasal and oropharyngeal swabs with a duration of 1 to 4 days where the sample was tested both locally in the hospital lab as well as corroborated with national lab testing to reduce risk of unequivocacy. Groups of patients were divided into either not swabbed (due to being asymptomatic) or swabbed due to presence of documented symptoms which yielded either negative or positive results.

For peer review only

For peer review only - http://bmjopen.bmj.com/site/about/guidelines.xhtml

Statistical analysis: All the data were recorded, anonymised and verified by four members of the study group for their accuracy. The data were processed using Microsoft Excel (Microsoft, Washington, USA). Shapiro-Wilk test indicated a normal distribution for age and days to discharge from hospital; hence, the mean (± standard deviation; 95% CI) were calculated for both. ASA did not follow normality and was analysed using median (±median absolute deviation [MAD]) and interquartile range (IQR). Both prevalence or risk and odds ratios were calculated as well as a Fisher's exact test for statistical significance for categorical data, defined as $p \leq 0.05$. Percentages and confidence intervals were rounded off to one decimal place.

The collaborative: The COVid Emergency Related Trauma and orthopaedics (COVERT) Collaborative was founded at Imperial College Healthcare NHS Trust. It is currently a member of the COVID Research Group and it has been endorsed by the Royal College of Surgeons of England and Imperial College Healthcare NHS Trust.

Patient and Public Involvement: Patients and the public were not involved in the study design, recruitment or conduct.

For peer review only - http://bmjopen.bmj.com/site/about/guidelines.xhtml

Results

A total of 4840 clinical encounters took place between the study periods. A total of 172 spinal operations and presentations were excluded from the final analysis. Table 2 outlined demographic data. During the COVID-19 period there was a 34% reduction in acute orthopaedic trauma referrals compared to 2019 (1792 down to 1183 referrals), and 29.5% less surgical interventions (993 down to 700 operations). Figures 1-3 have categorised these clinical encounters into acute referrals and operative cases between both years respectively.

Table 2: Demographic data of pre- and post-COVID

		Pre-COVID (2019)		COVID (2020)	
	**Total**	**1792**		**1183**	
**Acute referrals**	*Male*	935	52.2%	560	47.3%
	Female	857	47.8%	623	52.7%
	Mean Age ± SD (95% CI)	52.2 ± 27.9 (50.9 - 53.5)		55.8 ± 27.9 (54.3 - 57.4)	
**Operative cases**	**Total**	**993**		**700**	
	Male	499	50.3%	320	45.7%
	Female	494	49.7%	380	54.3%
	Mean Age ± SD (95% CI)	51.7 ± 28.1 (50 - 53.5)		57.7 ± 26.7 (55.7 - 59.6)	
	Median ASA ± MAD (IQR)	2 ± 1 (2)		2 ± 1 (2)	

Figure 1: Types and mechanisms of injuries for acute referrals

Figure 2: Types and mechanisms of injuries for operative cases

Figure 3: operative and anaesthetic techniques compared between pre- and post-COVID

For peer review only

COVID status

COVID status for overall patient groups including positive results in the mortalities in acute referrals and operative casemix were demonstrated in figure 4. Overall mortalities with positive swab results were confirmed prior to the event of death.

Figure 4: COVID status for acute referrals and operative cases as a measure of proportions

Risk (or prevalence) and odds ratios

Table 3: Risk (or prevalence) and odds ratios for acute referrals and operative caseloads.

Comparisons are made between COVID period against the pre-COVID period. Value >1

indicated greater odds or risk during the COVID period.

		Acute referrals			Operative caseload		
		RR	OR	p-value	RR	OR	p-value
Morbidity & Mortality	Mortality	2.50	2.55	0.0005	2.19	2.25	0.004
	Mortality due to COVID-related complications vs non-COVID causes	14.2	19.7	0.004	15.1	22.0	0.004
	Peri-/post-operative complications including COVID				5.88	6.09	0.00001
	Peri-/post-operative complications excluding COVID				3.65	3.72	0.003
	Peri-/post-operative COVID positive testing				32.6	23.4	0.0009

For peer review only

Anaesthetic technique	General anaesthetic only				1.22	1.61	0.00001
	General anaesthetic ± block				1.23	1.75	0.00001
Consultant involvement	Consultant-led operation				1.36	2.08	0.00001
Operation technique	Open reduction + internal fixation				0.81	0.74	0.007
	Dynamic hip screw				2.02	2.11	0.00001
	Removal of metal/foreign body				0.24	0.23	0.003
Mechanism of injury	Road traffic accident	0.58	0.56	0.001	0.45	0.43	0.00001
	Fall (<1.5m)	1.19	1.54	0.00001	1.17	1.49	0.0001
	Sporting injury	0.63	0.60	0.0005	0.64	0.61	0.003
	Infection	0.69	0.66	0.001	1.70	1.77	0.005
	Trauma call				0.55	0.52	0.0005
Type of injury	Neck of femur (NOF) fracture	1.44	1.57	0.00001	1.51	1.79	0.00001
	Lower limb (excl. NOF)	0.89	0.84	0.04	0.74	0.65	0.0001
Gender	Male	0.91	0.82	0.01			

Table 3 outlined the risk [RR] (or prevalence [PR]) and odds ratios [OR] alongside their 95% confidence intervals and statistical significance. The risk ratio is synonymous with the prevalence ratio. Only those factors that were statistically significant within the acute referrals and operative caseloads were included.

Morbidity and Mortality

For peer review only - http://bmjopen.bmj.com/site/about/guidelines.xhtml

Table 3 indicated that the mortality rate more than doubled significantly for both risk (RR=2.19-2.50) and odds (OR=2.25-2.55) ratios during the COVID period. This certainly has both statistical as well as clinical significance. COVID-related complications were still responsible for increasing the odds of mortality by 20 to 22 times within all mortalities from both acute referrals and operative cases (as compared to non-COVID causes for all mortality in the year 2019). Table 4 confirmed that the mean age of mortalities across the board were in the elderly patient population with a high median ASA grade. Males were consistently in the minority, while neck of femur fracture was the modal diagnosis due to falls and persistently in the majority, followed by lower limb injuries (figures 5-6).

At least 82% of operations were related to neck of femur fractures in which half of all operations during the COVID period involved anaesthetic AGPs. Whereas the mortalities from pre-COVID operations did not have Consultant-led (as primary surgeon) surgery, that increased to three-fifths of all operations conducted during the COVID period (figure 7). The mean date of presentation to hospital was one week ahead in year 2020 compared to a year ago but the time from admission to mortality differed only by a mean of less than a day in both categories.

Table 4: Patient demographics, date of injuries, and time to mortality

	Acute referrals		***Operative casemix***	
	**2019 (n=23)**	**2020 (n=38)**	**2019 (n=22)**	**2020 (n=34)**
Mortality	1.3%	3.2%	2.2%	4.9%
Mortality with COVID positive PCR result		0.9% (total)<br>28.0% (mortality cohort)		1.6% (total)<br>32.9% (mortality cohort)
Post-op morbidity			0.7%	4.1%

For peer review only

Age (years; mean±SD; 95% CI)	80.2 ± 16.4 (73.2 - 87.2)		77 ± 23 (67 - 88)		83.9±12.2 (78.7 - 89.1)		84.0±13.5 (79.4 - 88.5)	
Male	9	39%	16	42%	8	36%	15	44%
ASA (median±MAD; IQR)					3 ± 0 (1)		3 ± 0 (0)	
Date of injury (mean days±SD; 95%CI)	6/4 ± 11 (1/4 - 10/4)		31/3 ± 12 (26/3 - 5/4)		6/4 ±12 (1/4 - 11/4)		30/3 ±14 (25/3 - 4/4)	
Time from admission to mortality (mean days±SD; 95%CI)	10.3 ± 7.5 (7.1 - 13.5)		11 ± 10 (7 - 15)		14.3 ± 10.4 (9.8 - 18.7)		13.8 ± 10.4 (10.2 - 17.3)	

Figure 5: types and mechanism of injury for mortalities in acute referral cohorts

Figure 6: types and mechanism of injury for mortalities in operative casemix cohorts

Figure 7: Surgical and anaesthetic techniques utilised in mortalities as a means of proportions

Taking into account that COVID was a peri-operative complication since patients may have been symptomatic with COVID manifestations pre-operatively but only had the swab results return with a positive finding either pre- or post-operatively; the commonest post-operative complication in the COVID period was a hospital-acquired pneumonia but with negative COVID swab results or the decision not to test at all. The second most common post-operative complication in the year 2020 was extra-pulmonary sepsis (figure 8). The proportion of post-operative complications had significantly increased when including or excluding COVID as a peri- or post-operative complication in 2020 (0.70% vs. 2.57-4.14%; p=0.003) with varying odds (3.72-23.4) and risk (3.65-32.6) ratios (table 3).

For peer review only - http://bmjopen.bmj.com/site/about/guidelines.xhtml

Figure 8: Post-operative complications for both years

Figures 9-10 focused on the total number and nature of comorbidities within the mortality groups. Multiple contingency chi-square test was insignificant for both number of comorbidities and individual comorbidities between both years, except for cardiovascular and cerebrovascular disease in acute referrals.

Figure 9: Type of comorbidities for all mortalities in both years

Figure 10: number of comorbidities for all mortalities in both years

Survival probability

A six-week Kaplan-Meier survival probability analysis for mortalities between both years was plotted in figures 11-12.

Figure 11: Six-week Kaplan-Meier survival probability analysis for mortalities between pre- and post-COVID for acutely referred from the Emergency Department

Figure 12: Six-week Kaplan-Meier survival probability analysis for mortalities between pre- and post-COVID for those undergoing surgery

For peer review only - http://bmjopen.bmj.com/site/about/guidelines.xhtml

Discussion

Comment on alternative hypothesis

There was a significant difference between pre- and post-COVID periods at its peak. The alternative hypothesis was not rejected with respect to prevalence of (i) acute orthopaedic trauma referrals (reduced by 34%), (ii) surgical interventions (reduced by 29.5%), (iii) anaesthetic aerosolising-generating procedures, (iv) mortality rates (more than doubled in the COVID period), and (v) survival probability between pre- and post-COVID eras.

Corroboration of our results with current literature

The 34% reduction in acute trauma referrals is in keeping with previous single centre studies performed in the UK with results ranging between 26-59%.[10-13,15,16] As described in these previous studies we would attribute the overall reduction of trauma workload to be due to reduction in travel and outdoor activities during the national lockdown. MacDonald *et al*.[17] described a similar effect in their multi-centre study with a reduction of operative workload by 26.5% compared to 29.5% in our study. Sites recruited for this study confirmed that they continued to operate at their own facilities during the data collection period whereas some later used alternative and external facilities including private hospitals through NHS England pathways (as mentioned by Dayananda et al.[18]), which may have impacted nosocomial rates of COVID, morbidity and mortality. However, this would be difficult to assess since it would also depend on the diversions of the ambulance services to 'clean' versus 'contaminated' hospital sites.

Changes in trends during COVID

For peer review only - http://bmjopen.bmj.com/site/about/guidelines.xhtml

With respect to the acute referrals, patients had half (OR=0.52) the odds of presenting as a trauma call. This was due to the odds ratios of road traffic accidents, sporting injuries, infection, and lower limb injuries were significantly less (by 34-44%; OR=0.56-0.66) during the COVID period. Conversely, there was a significant rise in the odds of neck of femur fractures, falls, the use of anaesthetic AGP and Consultant-led operations; a finding also reflected by Arafa *et al.*[19]

Although the expectation was to minimise the use of aerosolising-generating anaesthetic procedures, there was in fact an increased prevalence of using general anaesthesia ± block up to an odds of 75%, in order to create a 'closed circuit' for the airways. As the anaesthetic methods was not well documented in the pre-COVID era in a fifth (21.3%) of cases, this skewed the data as it may have been difficult to extract that data from last year. The odds of a Consultant-led operation doubled (OR=2.08) during the COVID period as a consequence of all elective operations being suspended, more Consultants being relocated to trauma theatre and increased pressure within the theatre environments leading to Consultant-delivered, rather than Consultant-led care. With respect to surgical procedures, there was a significant reduction in prevalence ratio of open reduction and internal fixation by a fifth (PR=0.81) and removal of metalwork and foreign bodies by three-quarters (PR=0.24), while there was a doubling (PR=2.02) in dynamic hip screw fixation in the COVID era.

Morbidity and Mortality rates

Mortality during COVID-19 timeframe

Overall 6-week mortality rates significantly doubled for both prevalence (or risk) and odds ratios during the COVID-19 period (table 3). For mortalities within all acute referrals, 0.9% (figure 4) of the entire cohort and 28.9% of those mortalities tested positive for COVID. For

For peer review only - http://bmjopen.bmj.com/site/about/guidelines.xhtml

mortalities within the operative casemix, 1.6% (figure 4) of the entire cohort and 32.4% of those mortalities had a confirmed positive COVID-19 diagnosis prior to their death.

Comparatively, the COVIDSurg Collaborative observed a mortality rate of 28.8% (p<0.0001) of orthopaedic patients who underwent surgery (both elective and trauma) within the first quarter of the year.[20] The increased mortality during the pandemic is partly due to selection of cases that required surgical intervention. The decrease in acute referrals and operations indicated a higher threshold for treatment (due to a redistribution of hospital resources during the pandemic). However, no such case was denied surgery but in the worst-case scenario patients were offered postponed treatment. There are many cases with less severe orthopaedic trauma where there is a 2–3 week window of opportunity for acute operative management. Table 2 demonstrated that the COVID cohort on average was 6.9% older for the acute referrals and 11.6% older for the operative casemix which could be proportional to the risk of developing age-related and involutional morbidities and frailty.

Role of morbidity in mortality during COVID-19

Results from figures 8-10 were corroborated by the COVIDSurg publication[20] which confirmed a significant association of mortality with myocardial infarction and congestive heart failure. However, hypertension and stroke/transient ischemic attacks were not significantly associated. In our study, all cardiovascular diseases (including peripheral vascular, arrhythmias, hypertension, heart failure, myocardial infarction and acute coronary syndromes) were combined with cerebrovascular diseases (consisting of strokes and transient ischemic attacks). Unlike their study, our study did not find a significant association with chronic kidney disease, chronic obstructive disease (which included asthma) and dementia in all mortalities during the 2020 timeframe regardless of the COVID status. The differences

For peer review only - http://bmjopen.bmj.com/site/about/guidelines.xhtml

may stem from that their study looked at the comparison of mortality rates within the same cohort during the COVID era, whereas this study is sub-analysing the entire mortality cohort on its own to observe for specific associations and risks.

Survival probability between both years

There were similar patterns of survival probability between both cohorts (i.e. 2019 vs 2020 cumulative). However, the lowest survival probability and the shortest timeframe were observed in the confirmed COVID positive cohort as seen in figure 11. This was due to the most vulnerable patient profile. 8 (72.7%) patients had femoral trauma, most being neck of femur fractures, distal femur fracture and a dislocated hip hemiarthroplasty post-fracture. Other patients presented with septic arthritis, post-operative complication and knee swelling; yet every patient also suffered from multiple comorbidities including those leading to immunosuppression as seen in figures 11-12. Although these patients were prioritised in the Emergency Department and recognised for their poor physiological reserve, due to the stresses of the acute and emergency services, these patients may have had to wait longer to be treated acutely and appropriately admitted.

Unexpectedly, there was a reversal of trends observed for the 6-week Kaplan-Meier survival analysis once admitted and operated on in figure 12. Mortalities within the pre-COVID period had the lowest survival probability compared to the post-COVID cohort. The COVID positive mortalities were observed to have the highest survival probability 11 days prior to converging with those mortalities without COVID symptoms.

During the pandemic, wards were ring-fenced to host confirmed COVID positive patients with a heightened care of nursing, medical cover and personal protective equipment. Prior to

For peer review only - http://bmjopen.bmj.com/site/about/guidelines.xhtml

the onset of a possible vaccination to counteract the virus, symptomatic management and shielding were the mainstay treatments for COVID positive patients. None of these patients were stepped up to the Intensive Treatment Unit due to being categorised as high-risk stratification for mortality based on age and extent of comorbidities.

Justification of conducting this study

As lockdown measures in the UK and globally eases and the incidence of trauma returns to pre-lockdown trends, it is imperative that we understand the true increased risk of mortality in the acute trauma patient during the COVID-19 era. A recent publication by Kader et al.[21] has suggested that the rate of mortality from COVID-19 for elective orthopaedic patients is low; yet this is the first British multi-centre study to quantify mortality risk for trauma patients. Trauma procedures due to the nature of the injuries are necessary and time-critical, and nobody can afford to postpone trauma care even during a global pandemic.[22]

Furthermore, the Corona Hands Collaborative[23] published that upper limb trauma patients had SARS-CoV-2 complication rate of 0.18% (n=2) with 0.09% (n=1) overall mortality at the peak of the first wave in April 2020. However, their collaborative looked into a shorter post-operative period (30 vs 42 days) but they agreed that patients who had been hospitalised for a prolonged period before their surgery were at increased risk of both COVID-related and post-operative complications. Most of their patient cohort, who were both younger and fitter than our cohorts, would be classified as the 'walking wounded' and could usually be day-case procedures.

Although the trends in mechanisms of injury in our study were reflective of those within a US multi-centre study, there was an opposing trend in the number medical/surgical

For peer review only - http://bmjopen.bmj.com/site/about/guidelines.xhtml

procedures.[24] That could be due to their study encompassing on level 1 trauma centres with a mean younger patient population. However, we do agree that with time and from experiential learning, hospitals improved their coping strategies with the pandemic and enhanced patient safety by enforcing personal protection equipment, hosting dedicated theatres for COVID-positive patients, separating sites as clean and contaminated, ringfencing COVID-positive patients to dedicated wards, and promoting routine COVID PCR swabs for all admissions and pre-operative checklists.

With an overall mortality risk in 2020 doubled that of 2019, clinicians need to counsel patients presenting with acute orthopaedic trauma of the increased risk in the COVID-19 era, especially for those identified as increased risk stratification with multiple underlying comorbidities, elderly and frailty. With the ongoing risk of a subsequent wave and resurgence of COVID-19 cases on top of the inevitable winter pressures, this data is of critical importance in the risk management, decision-making and policymaking of trauma patients both in the UK and across the globe.

Observations of hip fractures and mortality

Since the aetiology of neck of femur fracture are often low energy falls in the home environment, it is not unexpected to observe a consistency of neck of femur fractures in the elderly and the vulnerable during lockdown as seen in figures 1-2. Those with neck of femur fractures remain at greatest risk of mortality and there have been further studies evaluating the risk of COVID-19 on this inherently high-risk cohort.[25-28] COVID-19 itself has been identified as an independent risk factor in increasing mortality in neck of femur fractures.[29]

For peer review only

The increased mortality reflect the increased proportion of NOFF patients that have a higher baseline mortality which has been echoed by the Scottish IMPACT-Restart study.[28] There are several justifications such as reduced help, lack of assistance and staff shortages due to the effect of the national lockdown which required elderly patients to be more independent, unsupervised and at higher risk of falling. Nevertheless, it should be considered that odds of falls may have increased due to prodromal symptoms and clinical manifestations of COVID.

However on subgroup analysis of hip fractures that were operated on in 2020, 20.2% tested positive for COVID, 47.3% tested negative and the remaining 32.4% were not tested due to being asymptomatic. Furthermore, 82.3% of all mortalities in 2020 sustained a neck of femur fracture in which only 35.7% of this cohort had a positive swab result, 21.4% with negative swab results and the remaining 42.9% were not swabbed due to being asymptomatic. There was no statistical difference in the odds and risk ratios between both years for mortality rate in NOFF. The numbers have not changed much, but because of a drop off of other cases, the percentage of NOFF markedly rose. Hence, the mortality expressed as a percentage of cases is notably higher for all operations, and not necessarily if stripped down to hip fractures alone.

If these 'at risk' patients were symptomatic with the virus, then aggressive pre-operative optimisation would occur. Since 91% (n=10) of COVID positive patients had sustained a neck of femur fracture, the National Hip Fracture Database best practice tariff of operating within an ideal 36-hour window set by the Royal College of Physicians was suspended until the patient was stabilised. All hip fracture patients in this cohort were operated on and had dedicated orthogeriatric input commencing from hospital admission. Hence the early peri-operative period and surgery encompassed within the 10-day period post-admission.

For peer review only - http://bmjopen.bmj.com/site/about/guidelines.xhtml

Moreover, neck of femur fractures are recognised as a pre-terminal illness and are known to carry a high risk of mortality in the first month which is trebled in the first year after the injury.[30]

Strengths and weaknesses of the study and in relation to other studies

This was the first representative observational multi-centre study of the UK looking into the impact of COVID-19 pandemic on general trauma and orthopaedic surgical specialty. Studies thus far have only shed light on local scales or cross-speciality.[10-13,20] Weaknesses included loss of data points which have been accounted for in the tables (i.e. labelled as unknown). However this did not affect the final analysis of data points. Operations conducted outside the specific study periods will not account for all those operations required such as for NOFF. It does not suggest that the number of NOFF not accounted for have been managed conservatively (as discovered by Cherevu et al.[31]), since some NOFFs may breach time to surgery due to medical reasons or being influenced by international guidelines.[32]

Limitations and future research

It is vital to continue exploring the impact of the pandemic on a larger scale. Ideally, more secondary care providers consisting of district general hospitals and major trauma centres will submit data. The diagnosis of COVID was dependent on positive PCR swabs for this study rather than non-specific changes seen on chest CT or plain radiographs. This does not account for false negatives with clinical respiratory symptomatology or true positives in those asymptomatic. Nevertheless, this issue with data has been speculated on in another national study.[23] Data ought to be submitted during the peak of the pandemic as well as at various time intervals as the lockdown measures ease resulting in more freedom of movement while also accounting for the continued risk of subsequent waves and national lockdowns.[33] Further

For peer review only - http://bmjopen.bmj.com/site/about/guidelines.xhtml

studies will also require to compare the impact of the pandemic on the speciality in the UK compared to other countries on other continents.

For peer review only

For peer review only - http://bmjopen.bmj.com/site/about/guidelines.xhtml

Conclusion

This was the first, longest and largest British multi-centre representation of the impact of COVID-19 pandemic on acute orthopaedic trauma referrals and mortality between mid-March to end-April, representing the peak of the first wave during the lockdown. The mortality rate for acute referrals, as well as those undergoing operative intervention, more than doubled in odds when compared to the same time interval one year ago. The majority of mortalities consisted of the elderly with neck of femur fractures and cardiovascular and/or cerebrovascular diseases. This study will aid clinicians in counselling trauma patients of the increased risk of mortality during the era of COVID-19 and also aid in both healthcare infrastructure, resource allocation, decision-making and policymaking as we continue to battle with the pandemic.

For peer review only

For peer review only - http://bmjopen.bmj.com/site/about/guidelines.xhtml

Research Ethics Approval - Human Participants: This study involves human participants but an Ethics Committee(s) or Institutional Board(s) exempted this study. All data points were utilised for routine auditing purposes to reflect departmental activity and service provision without altering clinical care pathways. Each centre contributing data to this study registered their interests with local authority and the auditing or clinical governance departments. No informed consent was required as there was no identifiable data. All data were anonymised at the time of collection and submission. Each patient was assigned a unique identification number which was cross-referenced with the patients' individual hospital identification or medical record numbers. This cross-referenced list remained internally within the hospital trust computer server handled by the contributing team from each trust. The data was transferred and stored using the NHS.net email server which has been approved for transfer of patient data. Data protection compliance was abided by at all times. The lead centre was Imperial College Healthcare NHS Trust where this study was first approved as a clinical audit prior to expanding onto a national scale. All centres gave permission for the use of their data. This study was assessed using the UKRI/MRC/NHS Health Research Authority Ethics Decision Tool and was considered an 'audit/not research'; and therefore it was not subject to further ethical review by the NHS Research Ethics Committee (NHS REC).

Competing Interests: None declared

Funding: Nil

Data sharing statement: Underlying data, code and supporting documentation may be made available as a redacted version to interested parties, subject to the completion of a protocol and signing of a Data Transfer Agreement.

For peer review only

505

506 **Acknowledgements:** none

For peer review only

For peer review only - http://bmjopen.bmj.com/site/about/guidelines.xhtml

References

1. World Health Organization (a). WHO Coronavirus Disease (COVID-19) Dashboard. Available online at https://covid19.who.int. Cited 2021 Sep 12.
2. Wu F, Zhao S, Yu B, Chen YM, Wang W, Song ZG, et al. A new coronavirus associated with human respiratory disease in China. *Nature.* 2020;579:265-9.
3. World Health Organization. Coronavirus disease 2019 (COVID-19): situation report, 66. 26 March 2020. https://www.who.int/docs/default-source/coronaviruse/situation-reports/20200326-sitrep-66-covid-19.pdf?sfvrsn=9e5b8b48_2. Cited 2021 Aug 30.
4. Spiteri G, Fielding J, Diercke M, Campese C, Enouf V, Gaymard A, et al. First cases of coronavirus disease 2019 (COVID-19) in the WHO European Region, 24 January to 21 February 2020. Eurosurveillance 2020 (25): 2000178.
5. UK Government (a) (2020) Oral statement to Parliament, Controlling the spread of COVID-19: Health Secretary's statement to Parliament. 16 Mar 2020. www.gov.uk/government/speeches/controlling-the-spread-of-covid-19-health-secretarys-statement-to-parliament. Cited 2021 Aug 25.
6. UK Government (b) (2020) Guidance: Oral statement to and away from others (social distancing). 23 Mar 2020. https://www.gov.uk/government/publications/full-guidance-on-staying-at-home-and-away-from-others. Cited 2021 Sept 5.
7. NHS England (2020a) Next steps on NHS response to COVID-19: Letter from Sir Simon Stevens and Amanda Pritchard. 17 Mar 2020. www.england.nhs.uk/coronavirus/publication/next-steps-on-nhs-response-to-covid-19-letter-from-simon-stevens-and-amanda-pritchard. Cited 2021 Aug 27.
8. Royal College of Surgeons. Guidance for surgeons working during the COVID-19 pandemic from the Surgical Royal Colleges of the United Kingdom and Ireland. March

For peer review only

20, 2020. www.rcseng.ac.uk/coronavirus/joint-guidance-for-surgeons-v1. Cited 2021 Aug 28.

9. British Orthopaedic Association. British Orthopaedic Association for Standards of Trauma (BOAST). Management of patients with urgent orthopaedic conditions and trauma during the coronavirus pandemic. March 24, 2020. www.boa.ac.uk/resources/statement-for-boa-members-on-trauma-and-orthopaedic-care-in-the-uk-during-coronavirus-pandemic.html. Cited 2021 Aug 28.

10. Park C, Sugand K, Nathwani D, Bhattacharya R, Sarraf KM. Impact of the COVID-19 pandemic on orthopaedic trauma workload in a London level 1 trauma centre: the "golden month". Acta Orthop. 2020 Jun 23;1-6.

11. Murphy T, Akehurst H, Mutimer J. Impact of the 2020 COVID-19 pandemic on the workload of the orthopaedic service in a busy UK district general hospital. Injury 2020;S0020-1383(20)30560-X.

12. Elhalawany A, Beastall J, Cousins G. The impact of the COVID-19 lockdown on orthopaedic emergency presentations in a remote and rural population, Bone Joint Open. 2020;1(10): 621-627.

13. Karia M, Gupta V, Zahra W, et al. The effect of COVID-19 on the trauma burden, theatre efficiency and training opportunities in a district general hospital: planning for a future outbreak, Bone Joint Open. 2020;1-8:494–499.

14. Office of National Statistics. Deaths registered weekly in England and Wales, provisional: 27 Aug, 2021. Released 7 Sept, 2021. www.ons.gov.uk/peoplepopulationandcommunity/birthsdeathsandmarriages/deaths/bullet

For peer review only

For peer review only - http://bmjopen.bmj.com/site/about/guidelines.xhtml

ins/deathsregisteredweeklyinenglandandwalesprovisional/27august2021. Cited 2021 Sep 12.

15. Atia F, Pocnetz S, Selby A, et al. The effect of the COVID-19 lockdown on hand trauma surgery utilization. *Bone Jt Open.* 2020;1(10):639-643.

16. Esteban PL, Querolt Coll J, Xicola Martínez M, et al. Has COVID-19 affected the number and severity of visits to a traumatology emergency department? *Bone Jt Open.* 2020;1(10):617-620.

17. MacDonald DRW, Neilly DW, Davies PSE, et al; SCORE authors. Effects of the COVID-19 lockdown on orthopaedic trauma: a multicentre study across Scotland. *Bone Jt Open*. 2020;1(9):541-548.

18. Dayananda KSS, Mercer ST, Agarwal R, et al. A comparative review of 1,004 orthopaedic trauma patients before and during the COVID-19 pandemic. Bone Jt Open. 2020;1(8):568-575.

19. Arafa M, Nesar S, Abu-Jabeh H, et al. COVID-19 pandemic and hip fractures: impact and lessons learned, Bone Joint Open. 2020;1-9:530–540.

20. COVIDSurg Collaborative. Mortality and pulmonary complications in patients undergoing surgery with perioperative SARS-CoV-2 infection: an international cohort study. *Lancet*. 2020 May 29.

21. Kader N. Clement ND. Patel VR. Caplan N. Banaszkiewicz P. Kader D. The theoretical mortality risk of an asymptomatic patient with a negative SARS-CoV-2 test developing COVID-19 following elective orthopaedic surgery. *Bone Joint J.* 2020;1-5.

For peer review only

22. Service BC, Collins AP, Crespo A, Couto P, Gupta S, Avilucea F, et al. Medically necessary orthopaedic surgery during the COVID-19 pandemic: safe surgical practices and a classification to guide treatment. *JBJS*. 2020;102(14):e76.

23. Dean BJF; Corona Hands Collaborative. Mortality and pulmonary complications in patients undergoing upper extremity surgery at the peak of the SARS-CoV-2 pandemic in the UK: a national cohort study. BMJ Qual Saf. 2020:bmjqs-2020-012156.

24. Salottolo K, Caiafa R, Mueller J, et al. Multicenter study of US trauma centres examining the effect of the COVID-19 pandemic on injury causes, diagnoses and procedures. *Trauma Surgery & Acute Care Open*. 2021;6:e000655.

25. Kayani B, Onochie E, Patil V, Begum F, Cuthbert R, Ferguson D, et al. The effects of COVID-19 on perioperative morbidity and mortality in patients with hip fractures. *Bone Joint J.* 2020;1-10.

26. Hall AJ, Clement ND, Farrow L, IMPACT-Scot Study Group, MacLullich AMJ, Dall GF, et al. IMPACT-Scot report on COVID-19 and hip fractures. *Bone Joint J.* 2020;1-10.

27. Clement ND, Hall AJ, Makaram NS, et al. IMPACT-Restart: the influence of COVID-19 on postoperative mortality and risk factors associated with SARS-CoV-2 infection after orthopaedic and trauma surgery. *Bone Joint J.* 2020;102-B(12):1774-1781.

For peer review only - http://bmjopen.bmj.com/site/about/guidelines.xhtml

28. Mackay N, Wilding C, Langley C, et al. The impact of COVID-19 on trauma and orthopaedic patients requiring surgery during the peak of the pandemic. *Bone Jt Open.* 2020;1-9:520–529.

29. Narang A, Chan G, Aframian A, et al. Thirty-day mortality following surgical management of hip fractures during the COVID-19 pandemic: findings from a prospective multi-centre UK study. *Int Orthop*. 2021;45(1):23-31.

30. British Orthopaedic Association. British Orthopaedic Association Standards for Trauma. January 2012. BOAST 1 Version 2 - Patients sustaining a Fragility Hip Fracture. www.boa.ac.uk/uploads/assets/6750e0bf-4aa3-4680-b1612265704512db/patients%20sustaining%20a%20fragility%20hip%20fracture.pdf. Cited 2021 Aug 25.

31. Cheruvu MS, Bhachu DS, Mulrain J, et al. Effect of COVID-19 on a rural orthopaedic hip fracture service. Bone Jt Open. 2020;1(8):500-507.

32. Lewis PM, Waddell JP. When is the ideal time to operate on a patient with a fracture of the hip? : a review of the available literature. Bone Joint J. 2016;98-B(12):1573-1581.

33. Adebowale V, Alderson D, Burn W, Dickson J, Godlee F, Goddard A, et al. Covid-19: Call for a rapid forward looking review of the UK's preparedness for a second wave-an open letter to the leaders of all UK political parties [published correction appears in BMJ.

For peer review only

For peer review only - http://bmjopen.bmj.com/site/about/guidelines.xhtml

2020 Jul 16;370:m2866. Dixon, Jeanette [corrected to Dickson, Jeanette]]. *BMJ.*
2020;369:m2514. Published 2020 Jun 23.

For peer review only

For peer review only - http://bmjopen.bmj.com/site/about/guidelines.xhtml

Contributorship statement according to the ICMJE guidelines

We have read the ICJME guidelines attentively and have outlined the collaborative contributors below who have all satisfied the criteria to be recognised as a collaborative co-author if this study is published. The core committee of the collaborative consists of KS, AA, CP and KMS who all conceptualised the study, led the planning/investigation/methodology/design, supervised the collaborative contributors' roles, as well as the initial and final version of the manuscript. Additionally, KS (primary author) was also leading data curation, project administration and resource allocation with KMS. KS was also leading on validation. KS, CP and AA led the data analysis and the reporting of the results. All other collaborative members, from the seven centres and outside the core committee, were involved in data curation, formal analysis and in resource allocation internally under consultant supervision (i.e. those with FRCS). Individual contributions from the core committee and every collaborative member has been outlined below:

COVERT Collaborative members, their affiliations and extent of contribution

Core Committee

Name	Qualification	Contribution	E-mail

For peer review only - http://bmjopen.bmj.com/site/about/guidelines.xhtml

1	**Kapil Sugand** Primary & corresponding author *58 Thames Avenue, Greenford, Middlesex, UB6 8JN* *E: ks704@ic.ac.uk* *T: 00447773642813*	MRCS	• Conceptualisation<br>• Data curation<br>• Formal analysis<br>• Investigation<br>• Methodology/Design<br>• Planning<br>• Project administration<br>• Resources<br>• Supervise<br>• Validation<br>• Data analysis<br>• Visualisation<br>• Writing original draft<br>• Writing – reviewing and editing	Ks704@ic.ac.uk<br>ORCID: 0000-0003-3185-954X
2	Arash Aframian	MRCS	• Conceptualisation<br>• Formal analysis<br>• Investigation<br>• Methodology/Design<br>• Planning<br>• Supervise<br>• Data analysis<br>• Writing – reviewing and editing	arash.aframian@gmail.com
3	Chang Park	MRCS	• Conceptualisation<br>• Methodology/Design<br>• Planning<br>• Supervise<br>• Data analysis<br>• Writing – reviewing and editing	changpark3@gmail.com
4	Khaled M Sarraf (senior author)	FRCS	• Conceptualisation<br>• Investigation<br>• Methodology/Design<br>• Planning<br>• Project administration<br>• Resources	khaled.sarraf@nhs.net

For peer review only - http://bmjopen.bmj.com/site/about/guidelines.xhtml

- Supervise
- Writing – reviewing and editing

COVERT Collaborators

Affiliation: Department of Trauma and Orthopaedics, Imperial College Healthcare NHS Trust, London, W2 1NY, England

	Name	Qualifi-cation	Contribution	E-mail
5	Thomas Ashdown	MRCS	• Data curation<br>• Formal analysis<br>• Resources	thomas.ashdown@nhs.net
6	Ramla Ali	MBBS	• Data curation<br>• Formal analysis<br>• Resources	ramla.ali@nhs.net
7	William Harland	MBBS	• Data curation<br>• Formal analysis<br>• Resources	william.harland@nhs.net
8	Charles Hallett	MBBS	• Data curation<br>• Formal analysis<br>• Resources	Ortho@pansurg.org
9	Chinmay Gupte	FRCS	• Project administration<br>• Resources<br>• Supervise	c.gupte00@ic.ac.uk
10	Dominic Spicer	FRCS	• Project administration<br>• Resources<br>• Supervise	dominic.spicer@nhs.net
11	Robin Strachan	FRCS	• Project administration<br>• Resources<br>• Supervise	robin.strachan@nhs.net

For peer review only

12	Dinesh Nathwani	FRCS	• Project administration<br>• Resources<br>• Supervise	d.nathwani@imperial.ac.uk
13	Rajarshi Bhattacharya	FRCS	• Project administration<br>• Resources<br>• Supervise	rajarshi.bhattacharya@nhs.net

Affiliation: Department of Trauma and Orthopaedics, Chelsea and Westminster Hospital NHS Foundation Trust, London, SW10 9NH, England

	Name	Qualification	Contribution	E-mail
14	Catrin Morgan	MRCS	• Data curation<br>• Formal analysis<br>• Project administration<br>• Supervise	catrin.morgan@nhs.net
15	Katharine Bennett-Brown	MRCS	• Data curation<br>• Formal analysis<br>• Resources	katebennettbrown@gmail.com
16	Henry Simon	MRCS	• Data curation<br>• Formal analysis<br>• Resources	henrysimon@hotmail.co.uk
17	Rupen Dattani	FRCS	• Project administration<br>• Resources<br>• Supervise	rupen.dattani@nhs.net

Affiliation: Department of Trauma and Orthopaedics, Queen Elizabeth Hospital, Gateshead Health NHS Foundation Trust, Gateshead, NE9 6SX, England

	Name	Qualification	Contribution	E-mail
18	Adham Mousa	MRCS	• Data curation<br>• Formal analysis<br>• Project administration	adham.mousa@nhs.net

For peer review only

			• Supervise	
19	Karen Vejsbjerg	MRCS	• Data curation<br>• Formal analysis<br>• Resources	karen.vejsbjerg1@nhs.net
20	Michael Zhang	MBBS	• Data curation<br>• Formal analysis<br>• Resources	michael.zhang1@nhs.net
21	Vikas Kumar	MBBS	• Data curation<br>• Formal analysis<br>• Resources	vikas.kumar7@nhs.net
22	Hannah Emerson	MBBS	• Data curation<br>• Formal analysis<br>• Resources	hannah.emerson2@nhs.net
23	Andrew Oliver	MBBS	• Data curation<br>• Formal analysis<br>• Resources	andrew.oliver2@nhs.net
24	Callum Smith	MBBS	• Data curation<br>• Formal analysis<br>• Resources	callum.smith8@nhs.net
25	Andreas Hinsche	FRCS	• Project administration<br>• Resources<br>• Supervise	a.hinsche@nhs.net

Affiliation: Department of Trauma and Orthopaedics, Darent Valley Hospital, Dartford and Gravesham NHS Trust, Dartford, DA2 8DA, England

	Name	Qualifi-cation	Contribution	E-mail
26	Markus L Sagmeister	MRCS	• Data curation<br>• Formal analysis<br>• Project administration<br>• Supervise	markus.sagmeister@nhs.net
27	Gihan Jayasinghe	MRCS	• Data curation<br>• Formal analysis<br>• Resources	gihan.jayasinghe@nhs.net

For peer review only - http://bmjopen.bmj.com/site/about/guidelines.xhtml

28	Marissa Lewis	MBBS	• Data curation<br>• Formal analysis<br>• Resources	marissa.lewis2@nhs.net
29	Michael Avery	MBBS	• Data curation<br>• Formal analysis<br>• Resources	michael.avery@nhs.net

Affiliation: Department of Trauma and Orthopaedics, Royal Berkshire NHS Foundation Trust, Reading, RG1 5AN, England

	**Name**	**Qualifi-cation**	**Contribution**	**E-mail**
30	Tariq Aboelmagd	MRCS	• Data curation<br>• Formal analysis<br>• Project administration<br>• Supervise	taboelmagd@doctors.org.uk
31	Shireen Ibish	MRCS	• Data curation<br>• Formal analysis<br>• Resources	shireen.ibish@nhs.net
32	Ruben Thumbadoo	MRCS	• Data curation<br>• Formal analysis<br>• Resources	ruben.thumbadoo@nhs.net
33	Asanka Wijendra	MRCS	• Data curation<br>• Formal analysis<br>• Resources	awijendra@gmail.com
34	Joseph Dixon	MRCS	• Data curation<br>• Formal analysis<br>• Resources	joedixon@doctors.org.uk
35	Vatsal Gupta	MRCS	• Data curation<br>• Formal analysis<br>• Resources	vatsalg001@gmail.com

Affiliation: Department of Trauma and Orthopaedics, James Cook University Hospital, South Tees NHS Foundation Trust, Middlesbrough, TS4 3BW, England

For peer review only - http://bmjopen.bmj.com/site/about/guidelines.xhtml

	Name	Qualification	Contribution	E-mail
36	Laura Clifton	MRCS	• Data curation<br>• Formal analysis<br>• Project administration<br>• Supervise	laura.clifton8@nhs.net
37	Suhib Taher	MRCS	• Data curation<br>• Formal analysis<br>• Resources	Suhib.taher@nhs.net
38	Nicholas Jia Jie Wei	MRCS	• Data curation<br>• Formal analysis<br>• Resources	nicholas.wei1@nhs.net
39	Emma Reay	FRCS	• Formal analysis<br>• Resources<br>• Supervise	emma.reay1@nhs.net

Affiliation: Department of Trauma and Orthopaedics, Aneurin Bevan University Health Board, Royal Gwent Hospital, Newport, NP20 2UB, Wales

	Name	Qualification	Contribution	E-mail
40	Ashish Khurana	FRCS	• Data curation<br>• Formal analysis<br>• Project administration<br>• Supervise	ashish.khurana@wales.nhs.uk
41	Kathryn Rooney	MRCS	• Data curation<br>• Formal analysis<br>• Resources	Kathryn.Rooney@wales.nhs.uk
42	Nakanda Kakaire	MRCS	• Data curation<br>• Formal analysis<br>• Resources	nakandamiriam.kakaire@wales.nhs.uk
43	Oluwawyemisi Adesina	MRCS	• Data curation<br>• Formal analysis<br>• Resources	quentinpz@yahoo.com

For peer review only

Figure 1: Types and mechanisms of injuries for acute referrals

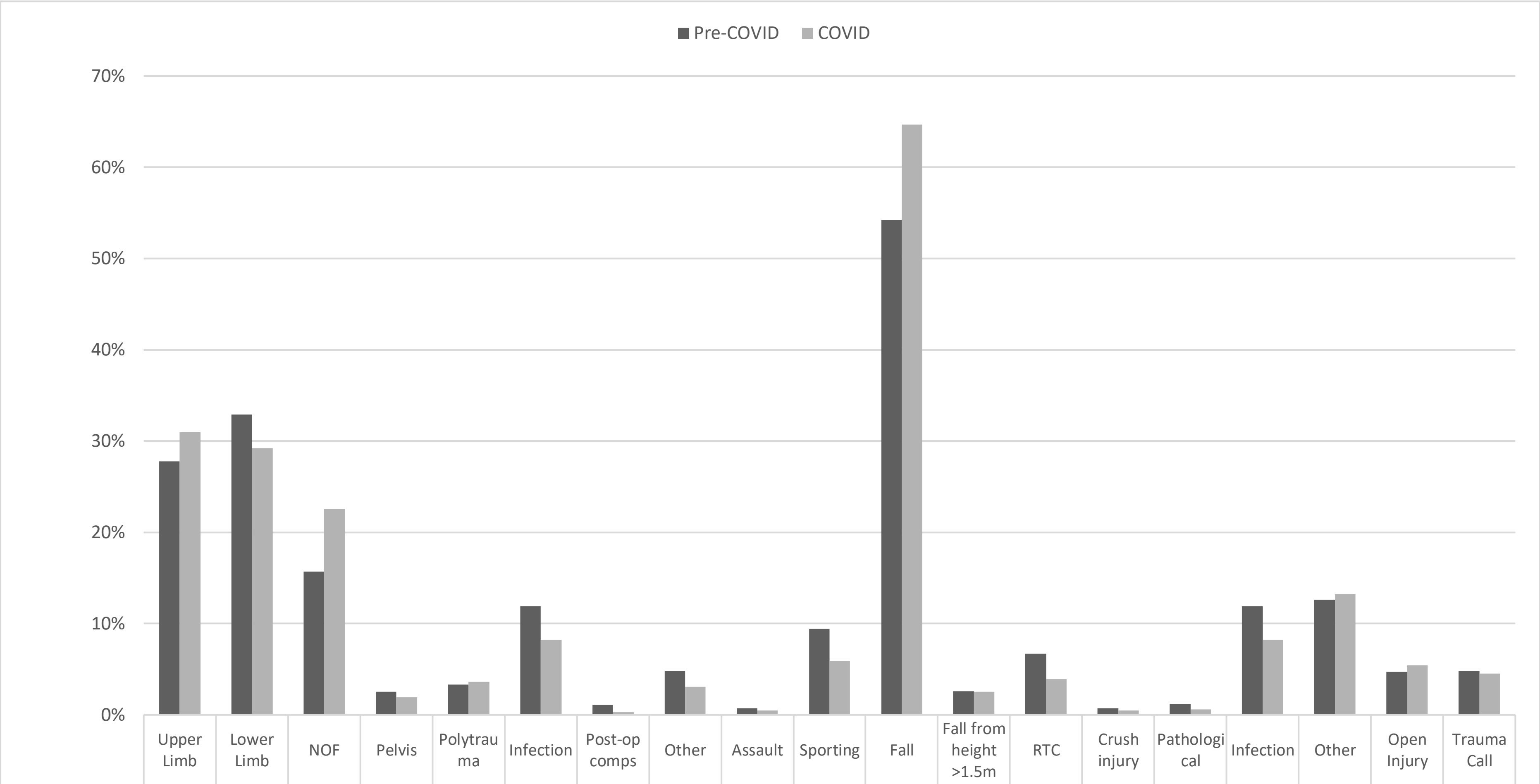

	Upper Limb	Lower Limb	NOF	Pelvis	Polytrauma	Infection	Post-op comps	Other	Assault	Sporting	Fall	Fall from height >1.5m	RTC	Crush injury	Pathological	Infection	Other	Open Injury	Trauma Call
Pre-COVID	27.8%	32.9%	15.7%	2.5%	3.3%	11.9%	1.1%	4.8%	0.7%	9.4%	54.2%	2.6%	6.7%	0.7%	1.2%	11.9%	12.6%	4.7%	4.8%
COVID	31.0%	29.2%	22.6%	1.9%	3.6%	8.2%	0.3%	3.1%	0.5%	5.9%	64.7%	2.5%	3.9%	0.5%	0.6%	8.2%	13.2%	5.4%	4.5%

Type of injuries | Mechanism of injuries

Figure 2: Types and mechanisms of injuries for operative cases

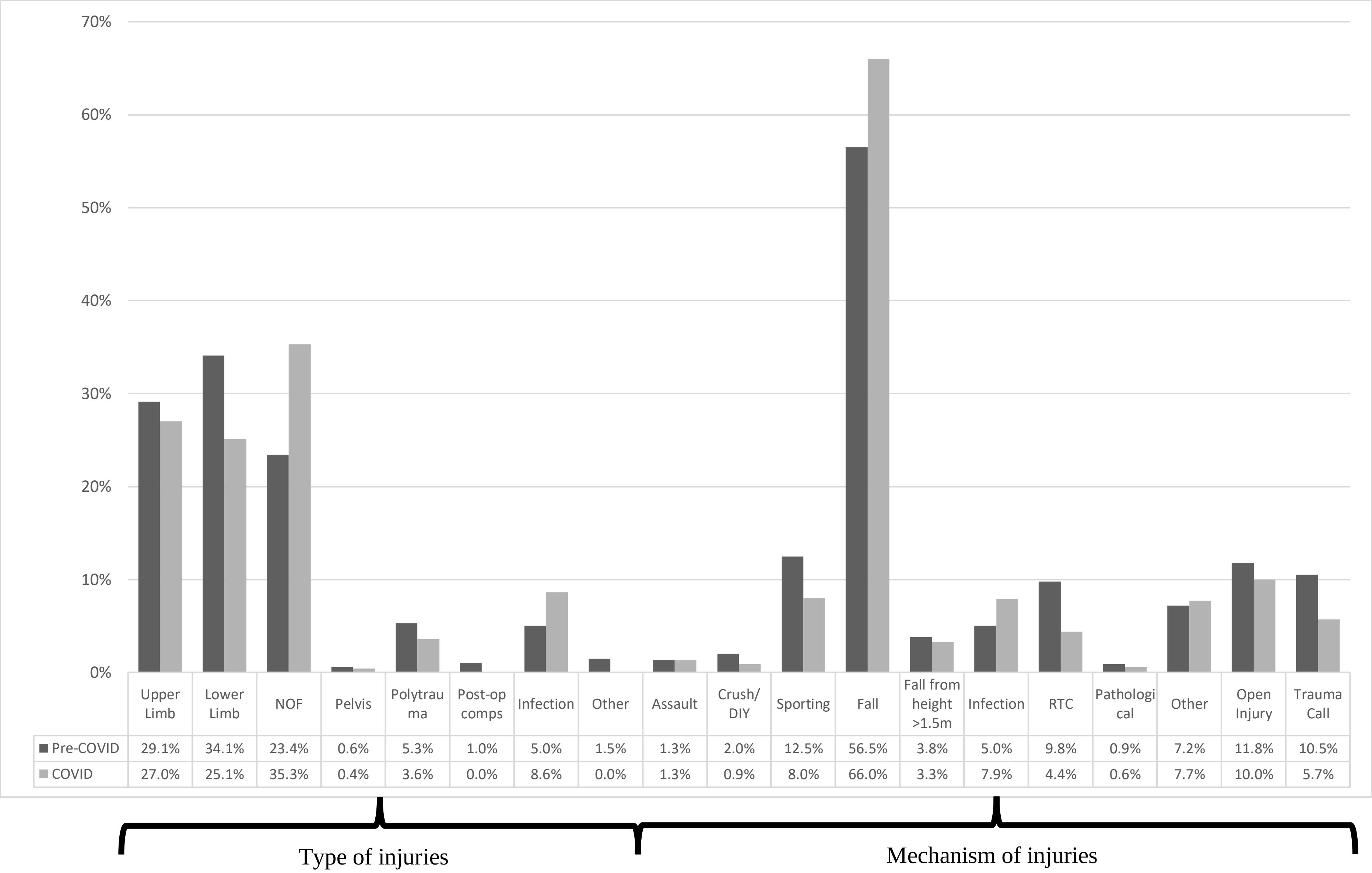

	Upper Limb	Lower Limb	NOF	Pelvis	Polytrau ma	Post-op comps	Infection	Other	Assault	Crush/ DIY	Sporting	Fall	Fall from height >1.5m	Infection	RTC	Pathologi cal	Other	Open Injury	Trauma Call
Pre-COVID	29.1%	34.1%	23.4%	0.6%	5.3%	1.0%	5.0%	1.5%	1.3%	2.0%	12.5%	56.5%	3.8%	5.0%	9.8%	0.9%	7.2%	11.8%	10.5%
COVID	27.0%	25.1%	35.3%	0.4%	3.6%	0.0%	8.6%	0.0%	1.3%	0.9%	8.0%	66.0%	3.3%	7.9%	4.4%	0.6%	7.7%	10.0%	5.7%

Figure 3: operative and anaesthetic techniques compared between pre- and post-COVID

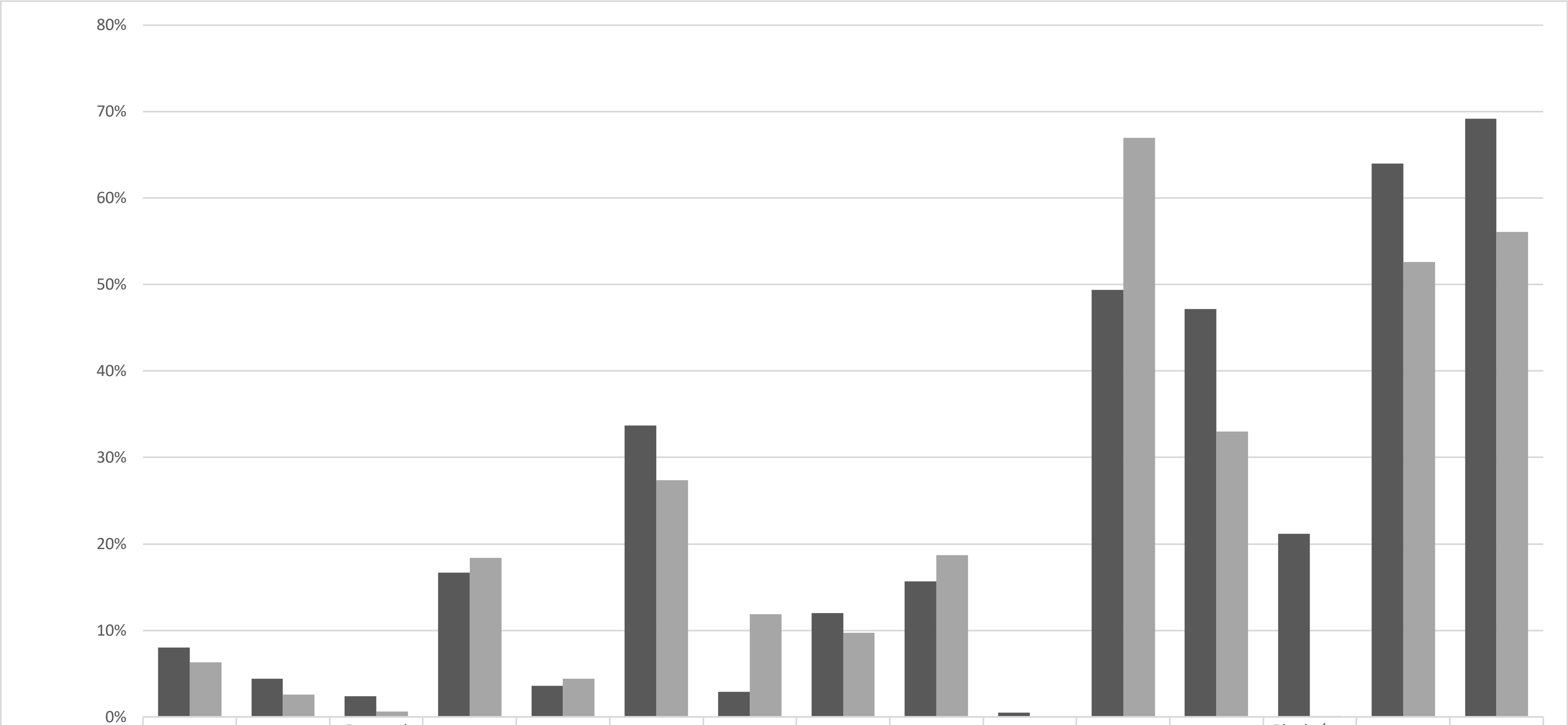

	MUA	ExFix	Removal metal/ FB	ST/Other	K-wire/CRIF	ORIF	DHS	IMN	Arthroplasty	Other	Consultant	Trainee	Blanks/ no document	GA only	GA +/- block
Pre-COVID	8.0%	4.4%	2.4%	16.7%	3.6%	33.7%	2.9%	12.0%	15.7%	0.5%	49.4%	47.2%	21.2%	64.0%	69.2%
COVID	6.3%	2.6%	0.6%	18.4%	4.4%	27.4%	11.9%	9.7%	18.7%	0.0%	67.0%	33.0%	0.1%	52.6%	56.1%

Type of injuries | Primary surgeon | Anaesthetic technique

Figure 4: COVID status of both cohorts including positive results in all mortalities (with 5% error bars)

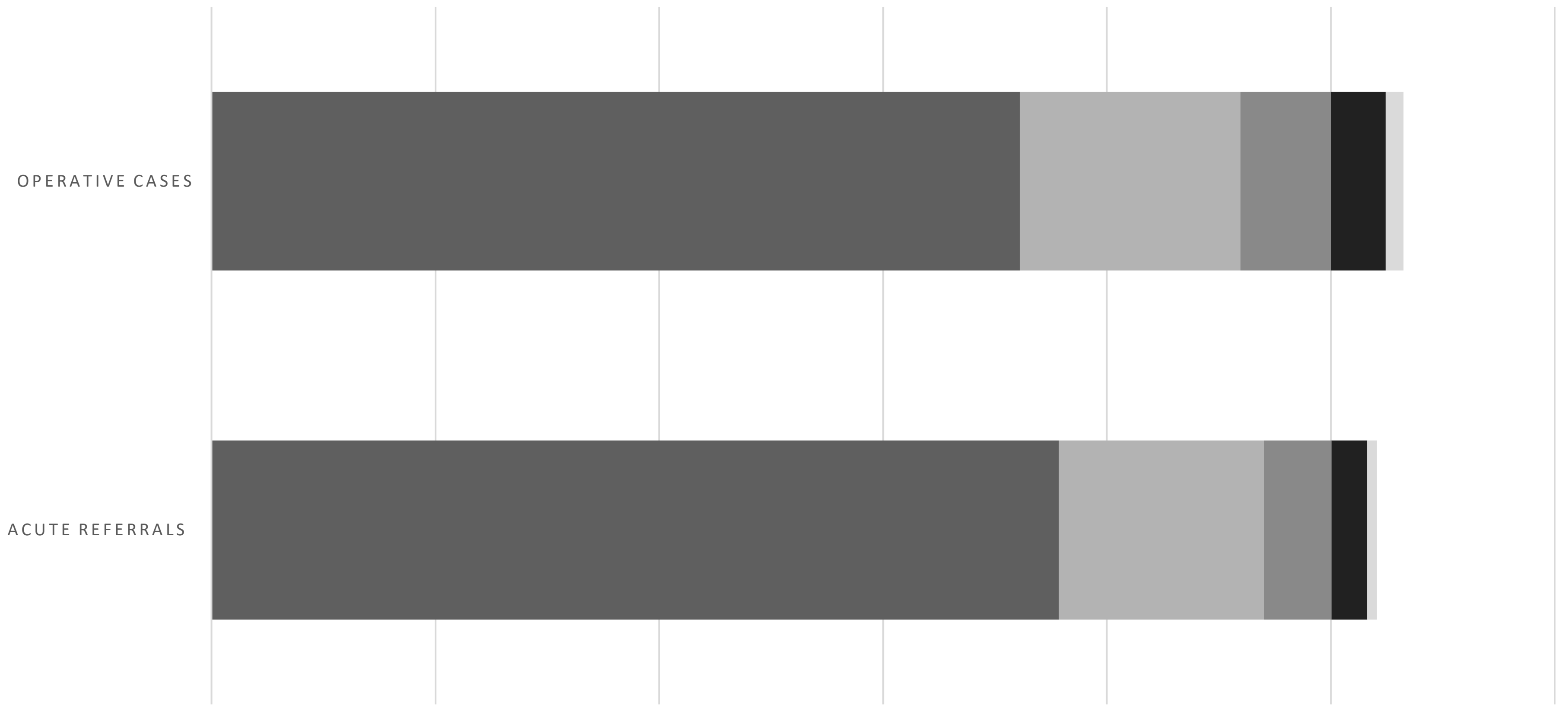

	Acute referrals	Operative cases
Not tested/asymptomatic/unknown	75.7%	72.2%
COVID -	18.3%	19.7%
COVID +	6.0%	8.1%
6-week Mortality	3.2%	4.9%
Mortality with COVID +	0.9%	1.6%

Figure 5: types and mechanism of injury for mortalities in acute referral cohorts

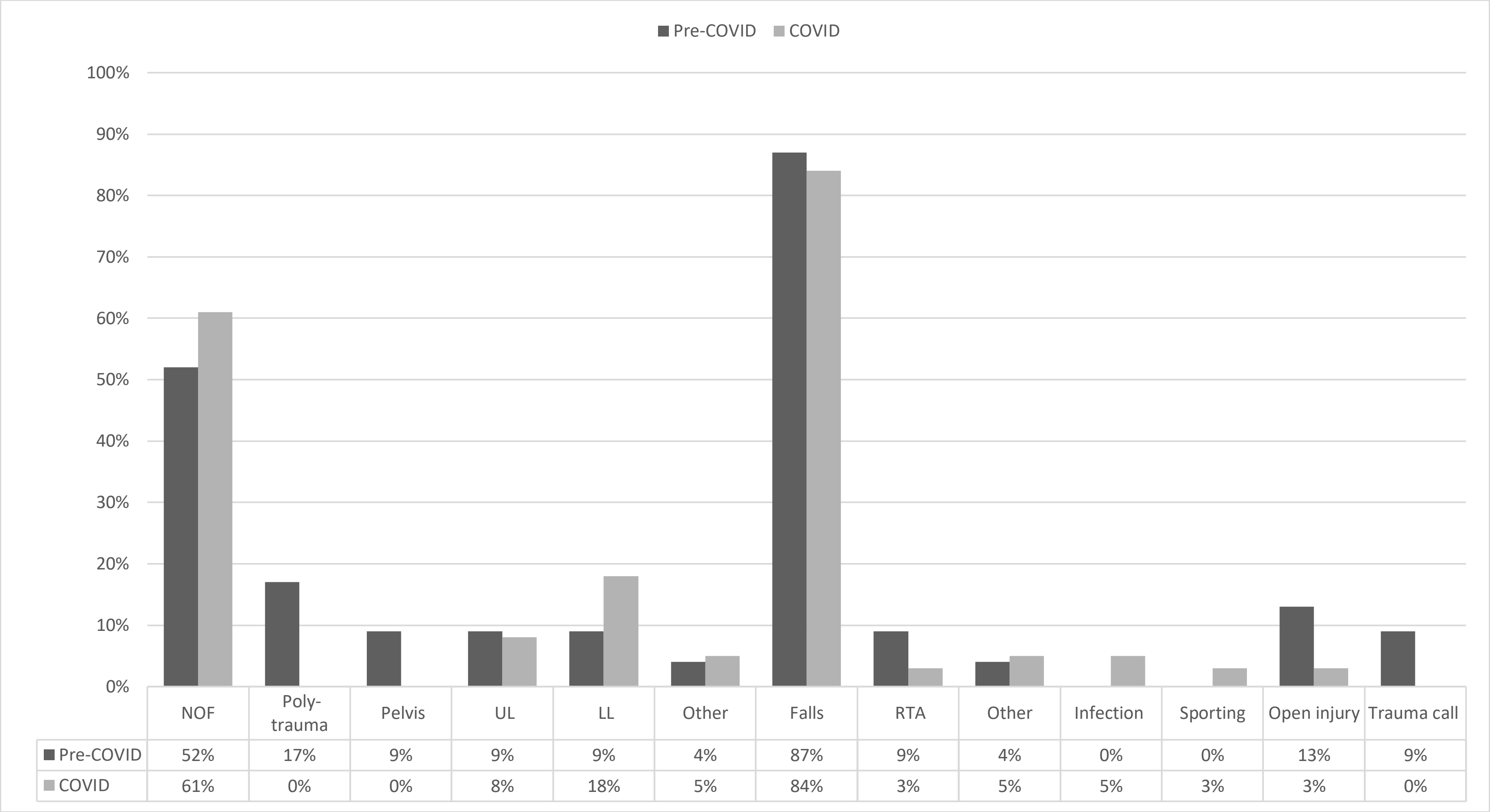

	NOF	Poly-trauma	Pelvis	UL	LL	Other	Falls	RTA	Other	Infection	Sporting	Open injury	Trauma call
Pre-COVID	52%	17%	9%	9%	9%	4%	87%	9%	4%	0%	0%	13%	9%
COVID	61%	0%	0%	8%	18%	5%	84%	3%	5%	5%	3%	3%	0%

***Key**: UL: upper limb / LL: lower limb / RTA: road traffic accidents*

Figure 6: types and mechanism of injury for mortalities in operative casemix cohorts

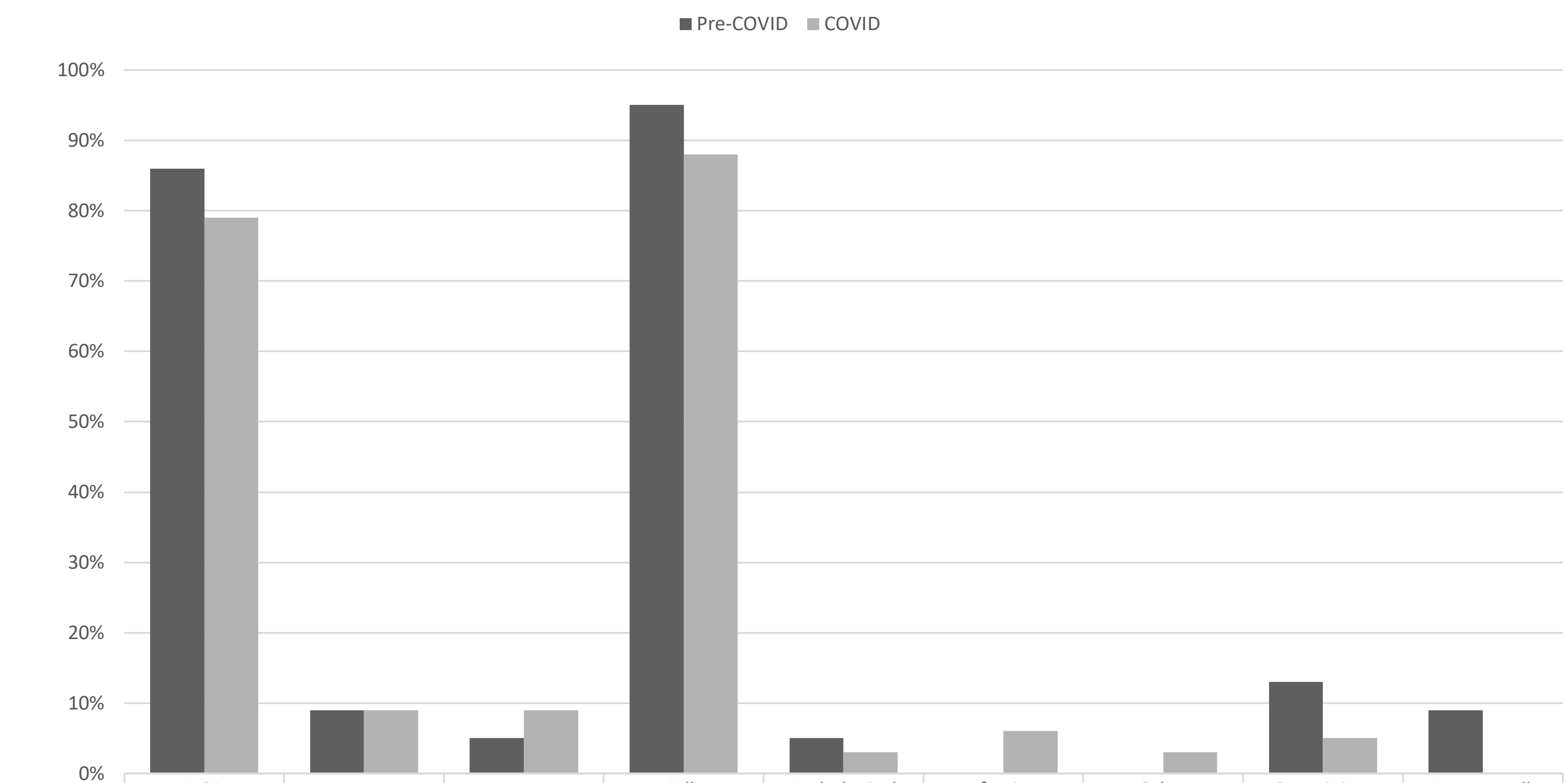

	NOF	LL	UL	Falls	Pathological	Infection	Other	Open injury	Trauma call
Pre-COVID	86%	9%	5%	95%	5%	0%	0%	13%	9%
COVID	79%	9%	9%	88%	3%	6%	3%	5%	0%

Key*: NOF: neck of femur fracture / UL: upper limb / LL: lower limb*

Figure 7: Surgical and anaesthetic techniques utilised in mortalities as a means of proportions

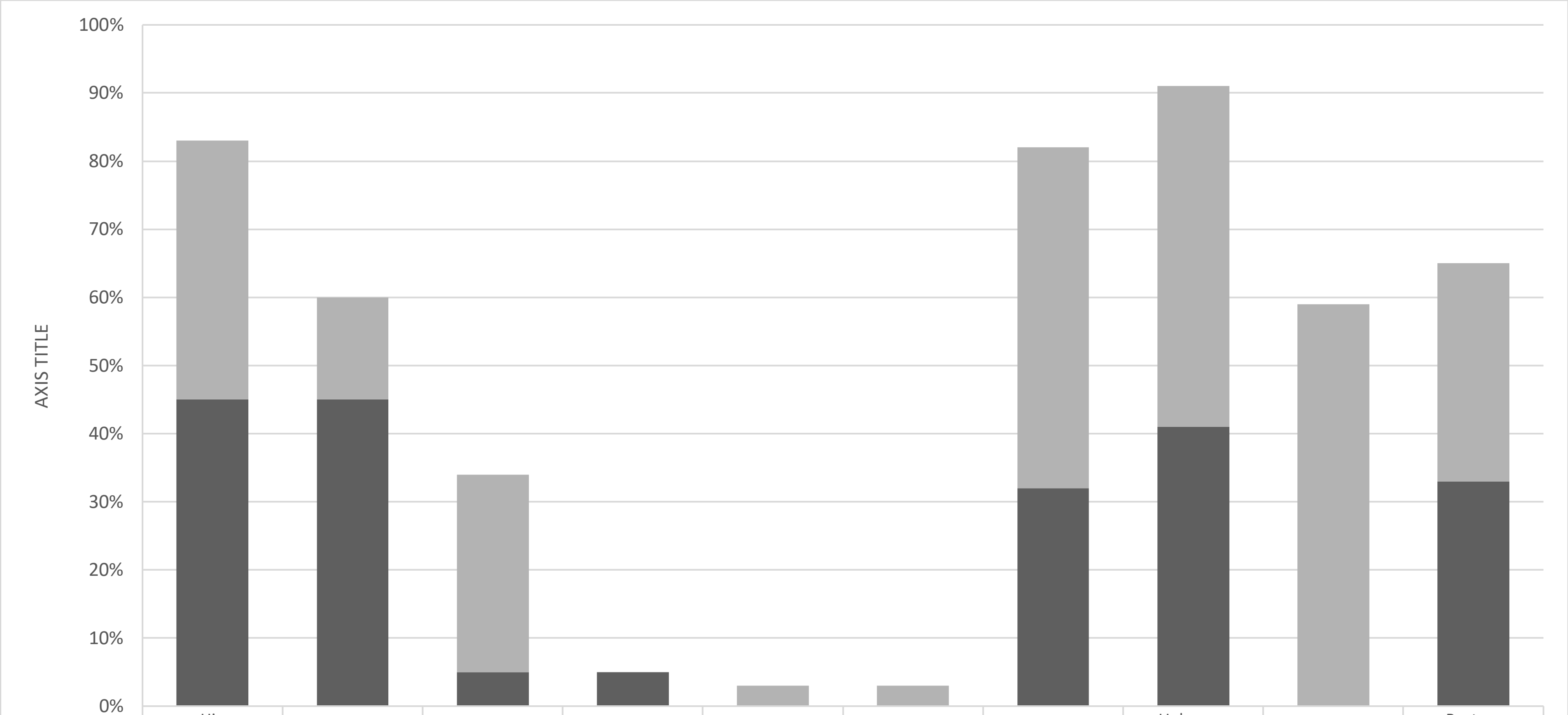

	Hip hemiarthroplasty	IMN	DHS	MUA	Soft tissue	Ex-Fix	AGP	Unknown anesthetic	Consultant-led	Post-op complications
COVID	38%	15%	29%	0%	3%	3%	50%	50%	59%	32%
Pre-COVID	45%	45%	5%	5%	0%	0%	32%	41%	0%	33%

***Key**: IMN: intramedullary nailing / DHS: dynamic hip screw / MUA: manipulation under anaesthesia / Ex-Fix: external fixation / AGP: aerosolizing-generating procedures*

Figure 8: Post-operative complications for both years

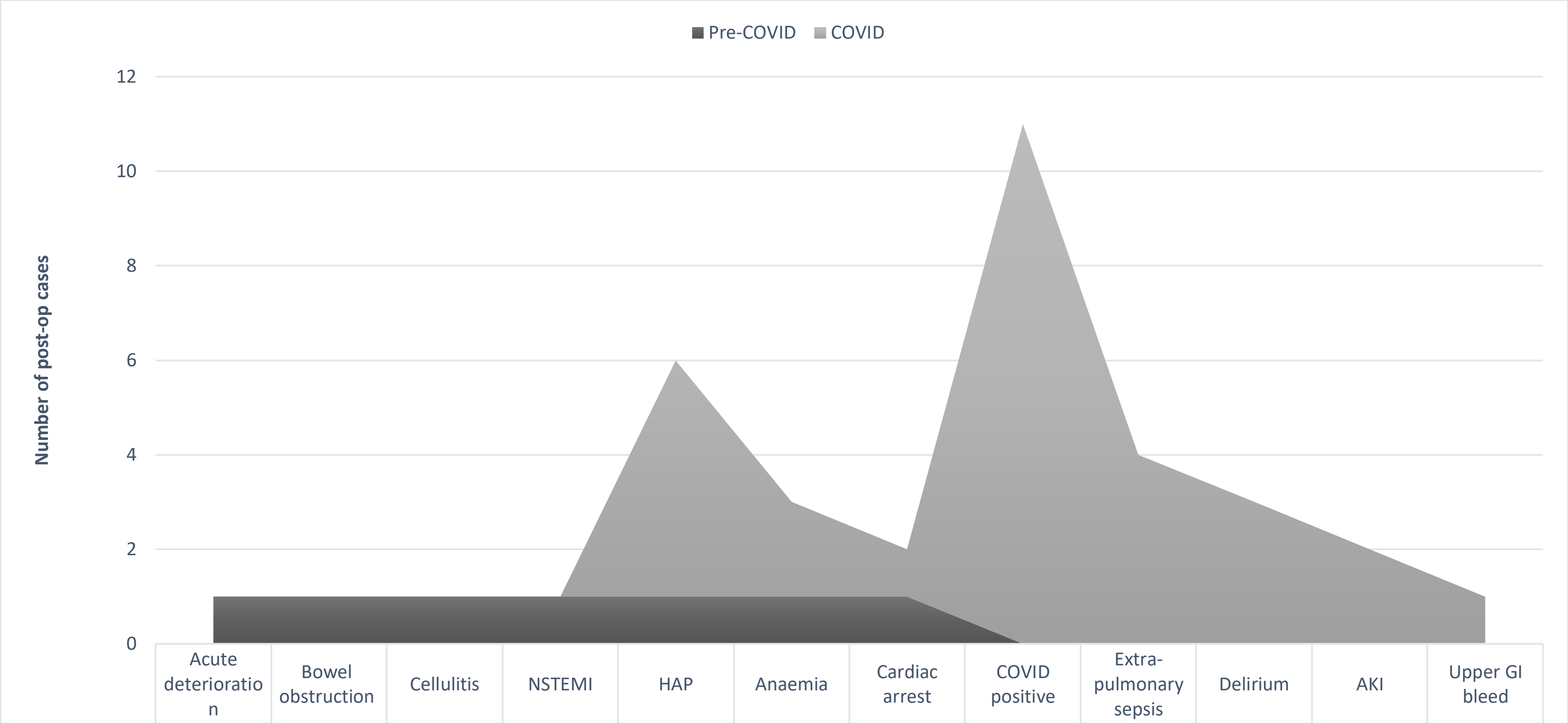

	Acute deterioration	Bowel obstruction	Cellulitis	NSTEMI	HAP	Anaemia	Cardiac arrest	COVID positive	Extra-pulmonary sepsis	Delirium	AKI	Upper GI bleed
COVID	0	0	0	0	5	2	1	11	4	3	2	1
Pre-COVID	1	1	1	1	1	1	1	0	0	0	0	0

Key: *NSTEMI: non-ST elevated myocardial infarction, HAP: hospital-acquired pneumonia, AKI: acute kidney injury, GI: gastrointestinal*

Figure 9: Type of comorbidities for all mortalities in both years

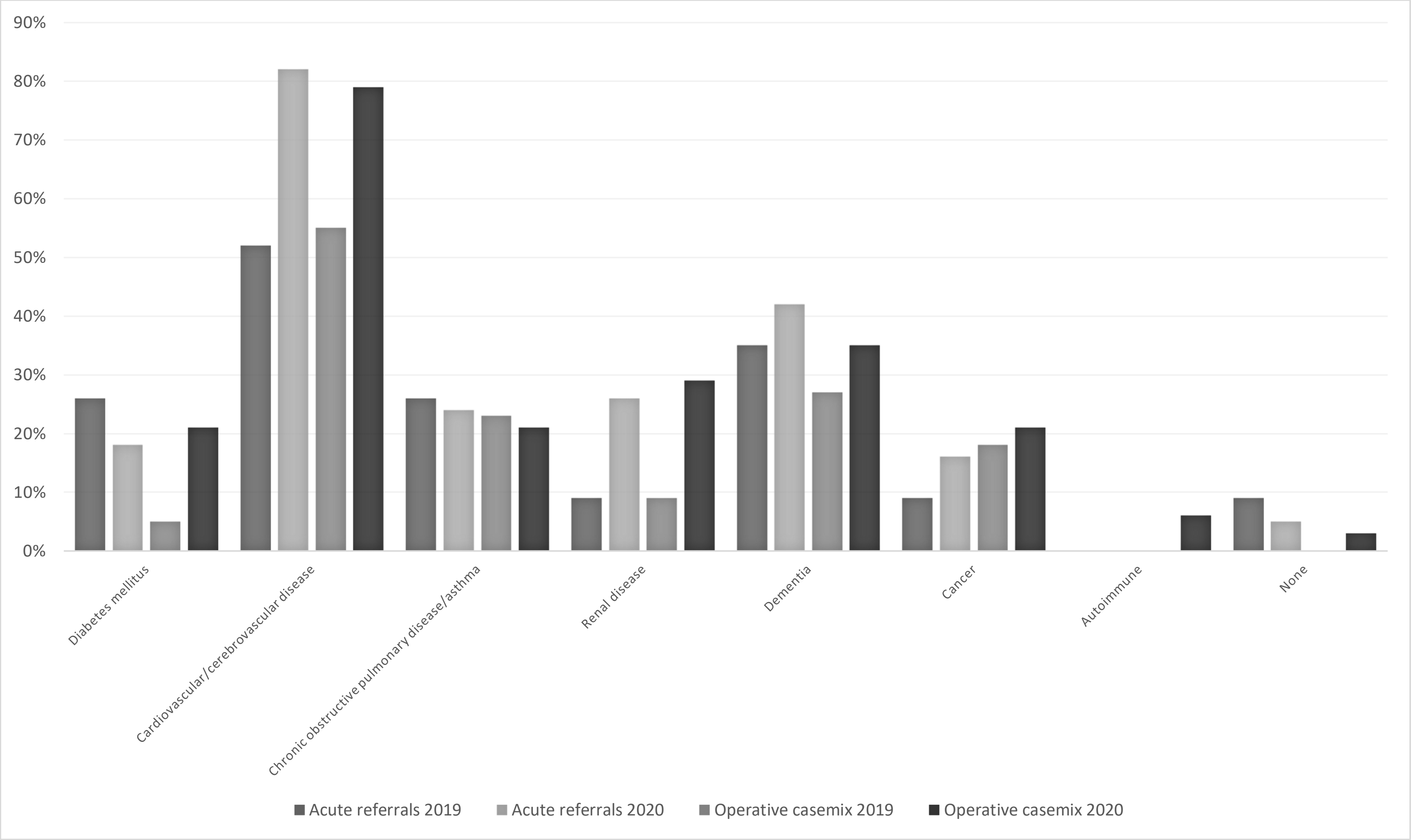

Figure 10: number of comorbidities for all mortalities in both years

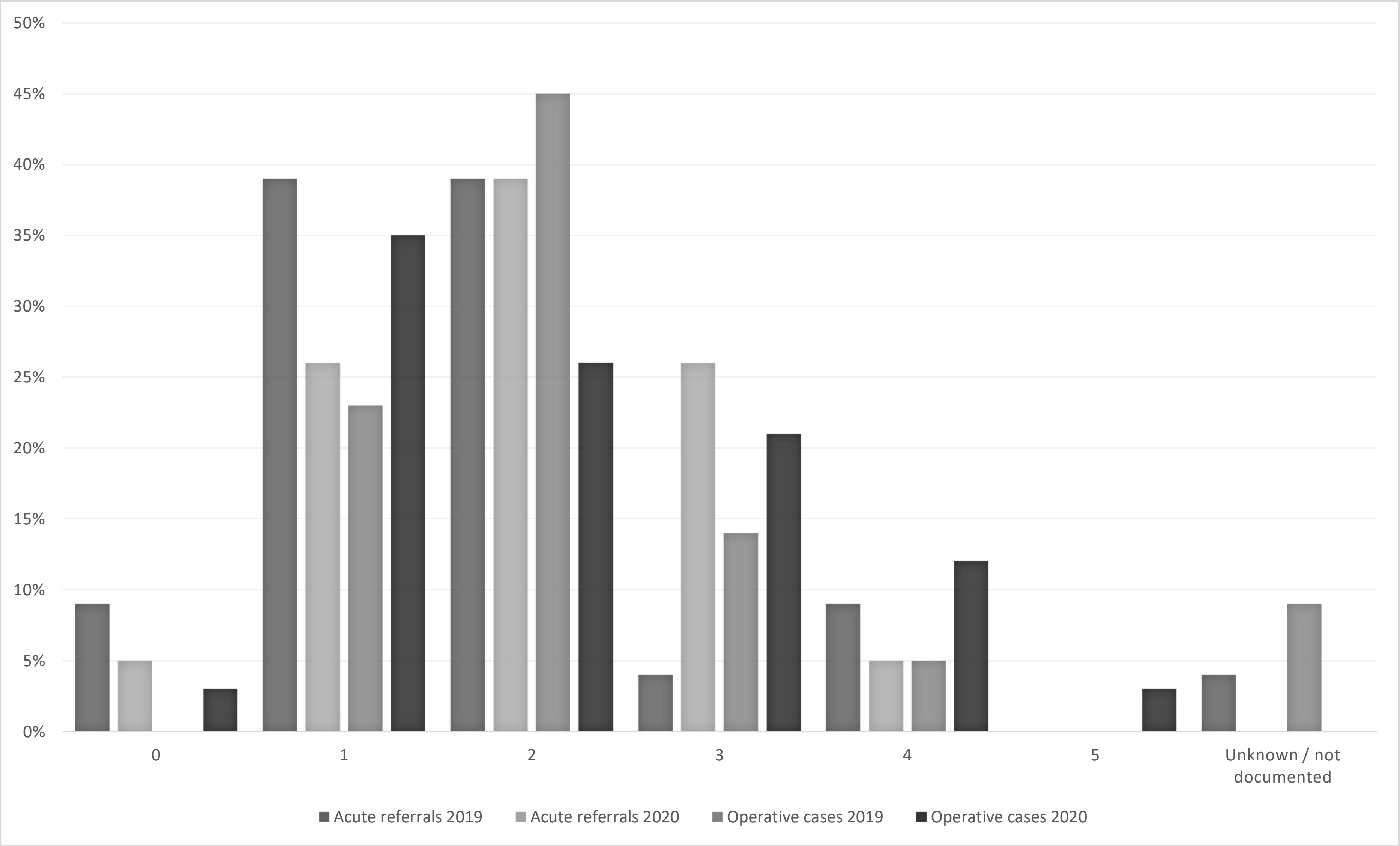

Figure 11: Six-week Kaplan-Meier survival probability analysis for mortalities between pre- and post-COVID for acutely referred from the Emergency Department

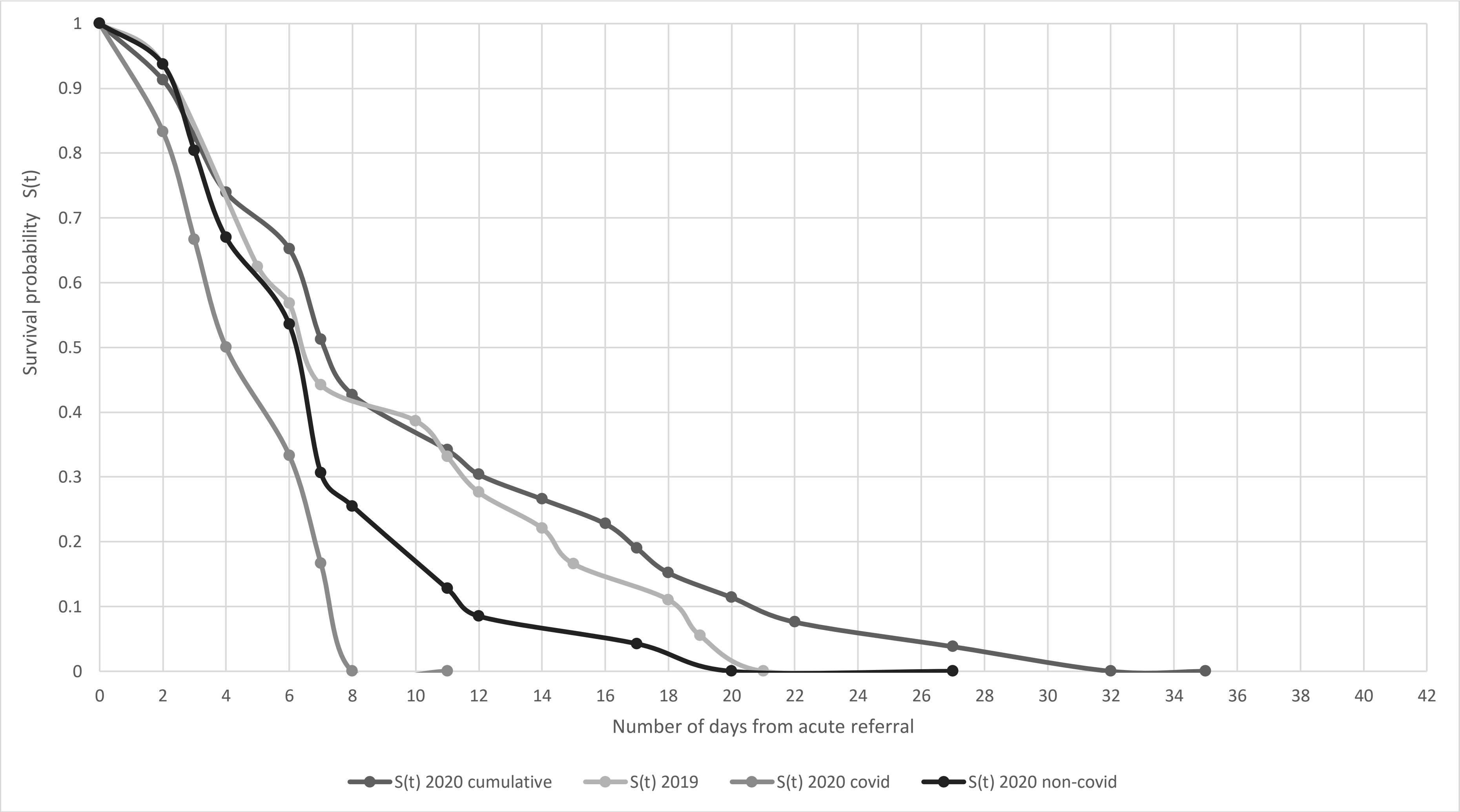

Figure 12: Six-week Kaplan-Meier survival probability analysis for mortalities between pre- and post-COVID for those undergoing surgery

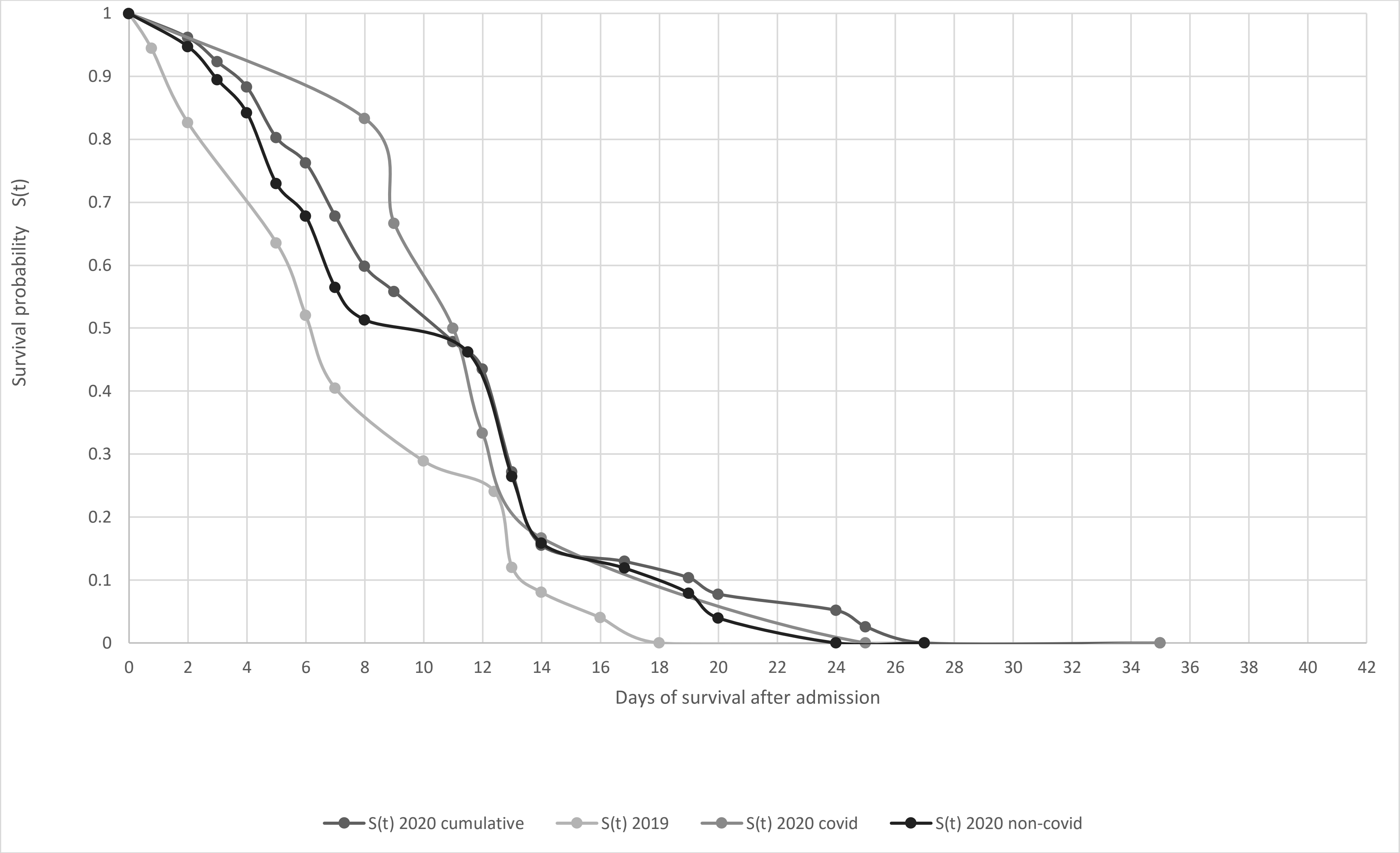

STROBE Statement—Checklist of items that should be included in reports of ***cohort studies***

	Item No	Recommendation	Page No
**Title and abstract**	1	(*a*) Indicate the study's design with a commonly used term in the title or the abstract	1, 7-8
		(*b*) Provide in the abstract an informative and balanced summary of what was done and what was found	8
**Introduction**			
Background/rationale	2	Explain the scientific background and rationale for the investigation being reported	9-10
Objectives	3	State specific objectives, including any prespecified hypotheses	10
**Methods**			
Study design	4	Present key elements of study design early in the paper	11
Setting	5	Describe the setting, locations, and relevant dates, including periods of recruitment, exposure, follow-up, and data collection	11-12
Participants	6	(*a*) Give the eligibility criteria, and the sources and methods of selection of participants. Describe methods of follow-up	12
		(*b*) For matched studies, give matching criteria and number of exposed and unexposed	12
Variables	7	Clearly define all outcomes, exposures, predictors, potential confounders, and effect modifiers. Give diagnostic criteria, if applicable	11-13 Table 1
Data sources/ measurement	8*	For each variable of interest, give sources of data and details of methods of assessment (measurement). Describe comparability of assessment methods if there is more than one group	11,14
Bias	9	Describe any efforts to address potential sources of bias	11-12
Study size	10	Explain how the study size was arrived at	11-12
Quantitative variables	11	Explain how quantitative variables were handled in the analyses. If applicable, describe which groupings were chosen and why	11-12, 14
Statistical methods	12	(*a*) Describe all statistical methods, including those used to control for confounding	14
		(*b*) Describe any methods used to examine subgroups and interactions	14
		(*c*) Explain how missing data were addressed	29
		(*d*) If applicable, explain how loss to follow-up was addressed	n/a
		(*e*) Describe any sensitivity analyses	n/a
**Results**			
Participants	13*	(a) Report numbers of individuals at each stage of study—eg numbers potentially eligible, examined for eligibility, confirmed eligible, included in the study, completing follow-up, and analysed	16 Table 2, Figs 1-3
		(b) Give reasons for non-participation at each stage	16
		(c) Consider use of a flow diagram	n/a
Descriptive data	14*	(a) Give characteristics of study participants (eg demographic, clinical, social) and information on exposures and potential confounders	16 Table 2, Figs 1-3
		(b) Indicate number of participants with missing data for each variable of interest	Figs 1-4
		(c) Summarise follow-up time (eg, average and total amount)	n/a

For peer review only

For peer review only - http://bmjopen.bmj.com/site/about/guidelines.xhtml

Outcome data	15*	Report numbers of outcome events or summary measures over time	Tables 2-4, Figures 1-12
Main results	16	(*a*) Give unadjusted estimates and, if applicable, confounder-adjusted estimates and their precision (eg, 95% confidence interval). Make clear which confounders were adjusted for and why they were included	9-15, Tables 2-4, Figs 1-12
		(*b*) Report category boundaries when continuous variables were categorized	n/a
		(*c*) If relevant, consider translating estimates of relative risk into absolute risk for a meaningful time period	Table 3
Other analyses	17	Report other analyses done—eg analyses of subgroups and interactions, and sensitivity analyses	20-21 Table 4 Figs 8-12
**Discussion**			
Key results	18	Summarise key results with reference to study objectives	22-29
Limitations	19	Discuss limitations of the study, taking into account sources of potential bias or imprecision. Discuss both direction and magnitude of any potential bias	29-30
Interpretation	20	Give a cautious overall interpretation of results considering objectives, limitations, multiplicity of analyses, results from similar studies, and other relevant evidence	22-29
Generalisability	21	Discuss the generalisability (external validity) of the study results	22-29
**Other information**			
Funding	22	Give the source of funding and the role of the funders for the present study and, if applicable, for the original study on which the present article is based	15

*Give information separately for exposed and unexposed groups.

Note: An Explanation and Elaboration article discusses each checklist item and gives methodological background and published examples of transparent reporting. Information on the STROBE Initiative is available at http://www.strobe-statement.org.

For peer review only

BMJ Open

The impact of COVID-19 on acute trauma and orthopaedic referrals and surgery in the UK during the first wave of the pandemic: a multicentre observational study from the COVid-Emergency Related Trauma and orthopaedics (COVERT) Collaborative

Journal:	*BMJ Open*
Manuscript ID	bmjopen-2021-054919.R2
Article Type:	Original research
Date Submitted by the Author:	13-Dec-2021
Complete List of Authors:	Sugand, Kapil; Imperial College London, MSk Lab<br>Aframian, Arash; Imperial College London, MSk Lab<br>Park, Chang; Imperial College London, Trauma and orthopaedics<br>Sarraf , Khaled; Imperial College London, Trauma and Orthopaedics<br>Collaborative, COVERT; Imperial College London
**Primary Subject Heading**:	Surgery
Secondary Subject Heading:	Emergency medicine, Epidemiology, Surgery, Medical management, Health services research
Keywords:	COVID-19, TRAUMA MANAGEMENT, ORTHOPAEDIC & TRAUMA SURGERY, EPIDEMIOLOGY

SCHOLARONE™
Manuscripts

I, the Submitting Author has the right to grant and does grant on behalf of all authors of the Work (as defined in the below author licence), an exclusive licence and/or a non-exclusive licence for contributions from authors who are: i) UK Crown employees; ii) where BMJ has agreed a CC-BY licence shall apply, and/or iii) in accordance with the terms applicable for US Federal Government officers or employees acting as part of their official duties; on a worldwide, perpetual, irrevocable, royalty-free basis to BMJ Publishing Group Ltd ("BMJ") its licensees and where the relevant Journal is co-owned by BMJ to the co-owners of the Journal, to publish the Work in this journal and any other BMJ products and to exploit all rights, as set out in our licence.

The Submitting Author accepts and understands that any supply made under these terms is made by BMJ to the Submitting Author unless you are acting as an employee on behalf of your employer or a postgraduate student of an affiliated institution which is paying any applicable article publishing charge ("APC") for Open Access articles. Where the Submitting Author wishes to make the Work available on an Open Access basis (and intends to pay the relevant APC), the terms of reuse of such Open Access shall be governed by a Creative Commons licence – details of these licences and which Creative Commons licence will apply to this Work are set out in our licence referred to above.

Other than as permitted in any relevant BMJ Author's Self Archiving Policies, I confirm this Work has not been accepted for publication elsewhere, is not being considered for publication elsewhere and does not duplicate material already published. I confirm all authors consent to publication of this Work and authorise the granting of this licence.

For peer review only

The impact of COVID-19 on acute trauma and orthopaedic referrals and surgery in the UK during the first wave of the pandemic: a multicentre observational study from the COVid-Emergency Related Trauma and orthopaedics (COVERT) Collaborative

Kapil Sugand, Arash Aframian, Chang Park, Khaled M Sarraf, COVERT Collaborative

Department of Trauma and Orthopaedics, Imperial College Healthcare NHS Trust, London, UK

Corresponding author:

Kapil Sugand

Dept T&O, St Mary's Hospital, Praed St, London, W2 1NY

E: ks704@ic.ac.uk

T: 07773642813

Keywords: COVID-19; orthopaedic trauma; UK multi-centre; pandemic wave; mortality

Word count: 4000 words

For peer review only - http://bmjopen.bmj.com/site/about/guidelines.xhtml

Abstract

Objective: This is the first British multi-centre study observing the impact of the COVID-19 pandemic on orthopaedic trauma with respect to referrals, operative caseload and mortality during its peak.

Design: A longitudinal, multi-centre, retrospective, observational, cohort study was conducted during the peak 6 weeks of the first wave from March 17, 2020 compared to the same period in 2019.

Setting: Hospitals from six major urban cities were recruited around the UK, including London.

Participants: A total of 4840 clinical encounters were initially recorded. 4668 clinical encounters were analysed post-exclusion.

Primary and secondary outcome measures: Primary outcomes included the number of acute trauma referrals and those undergoing operative intervention, mortality rates, and the proportion of patients contracting COVID-19. Secondary outcomes consisted of the mechanism of injury, type of operative intervention and proportion of aerosolising-generating anaesthesia utilised.

Results: During the COVID-19 period there was a 34% reduction in acute orthopaedic trauma referrals compared to 2019 (1792 down to 1183 referrals), and a 29.5% reduction in surgical interventions (993 down to 700 operations). The mortality rate was more than doubled for both risk and odds ratios during the COVID period for all referrals (1.3% vs 3.8%, p=0.0005) and for those undergoing operative intervention (2.2% vs 4.9%, p=0.004). Moreover, mortality due to COVID-related complications (versus non-COVID causes) had greater odds by a factor of at least 20 times. For the operative cohort during COVID, there was an increase in odds of aerosolising-generating anaesthesia (including those with

For peer review only

superimposed regional blocks) by three-quarters, as well as doubled odds of a consultant acting as the primary surgeon.

Conclusion: Although there was a reduction of acute trauma referrals and those undergoing operative intervention, the mortality rate still more than doubled in odds during the peak of the pandemic compared to the same time interval one year previous.

Keywords: COVID-19; orthopaedic trauma; UK multi-centre; pandemic wave; mortality

Strengths and limitations of this study

- This was the first representative observational study of the UK looking into the impact of COVID-19 pandemic on general trauma and orthopaedic surgical specialty.
- There is a valid comparison between two timeframes, exactly one year apart to represent pre-COVID and during COVID.
- Other studies thus far have only shed light on local scales or cross-speciality within a shorter timeframe than this study and not necessarily commenting on mortality rates like this study.
- Weaknesses included loss of data points which have been accounted for in the tables (i.e. labelled as unknown) which did not affect the final analysis of data points.

For peer review only - http://bmjopen.bmj.com/site/about/guidelines.xhtml

- Operations conducted outside the specific study periods will not account for all those operations required such as for hip fractures.

For peer review only

For peer review only - http://bmjopen.bmj.com/site/about/guidelines.xhtml

Introduction

The Global Impact of COVID-19

The COVID-19 pandemic will be remembered as one of the most unprecedented global health crises in modern history. With over 5 million deaths and over 263 million recorded cases globally, the pandemic has had a permanent impact on healthcare at the time of article submission.[1] The viral outbreak was first reported in December 2019 with the first patient hospitalised in the city of Wuhan, China.[2] By mid-March the outbreak affected over 190 countries with over 450,000 cases and over 20,000 deaths, thus being declared a pandemic and a global public health emergency by the World Health Organization.[3] On January 24th 2020 Europe reported its first case followed by a case in the United Kingdom (UK) 5 days later.[4]

The British Response to the pandemic

The English government responded by implementing social distancing measures on the 17th March 2020 in an attempt to reduce the rate of transmission and therefore the demands on the National Health Service (NHS).[5] This was followed a week later by more stringent measures, commonly referred to as a societal 'lockdown'.[6] As of the 23rd March 2020, all members of the public were required to stay at home. The NHS has also been deeply affected by the strain imposed by the virus as the healthcare infrastructure has had to evolve to cope with the overwhelming and unexpected pressures on staff, resources and finances. There has been a complete renovation of emergency medicine and orthopaedic services to manage musculoskeletal disease and trauma. In response to the NHS emergency declaration,[7] the Royal Colleges of Surgeons[8] and the British Orthopaedic Association[9] both issued statements and guidelines for delivering emergency trauma and orthopaedic care during the COVID-19 outbreak. The lockdown to limit the spread of the virus has had an unforeseen effect in

For peer review only - http://bmjopen.bmj.com/site/about/guidelines.xhtml

significantly reducing the acute trauma workload described in several single centre studies.[10-13] There has however not been a British multi-centre reflection of the impact of the COVID-19 pandemic on the orthopaedic workload and its potential impact on the mortality.

Aim

To observe the impact of COVID-19 on trauma and orthopaedic acute referrals, operative casemix and mortality rates during the peak 6 weeks of the first wave of the pandemic compared to the same time interval in 2019.

Alternative hypothesis

When comparing both years, there would be a difference in the prevalence of acute orthopaedic referrals, orthopaedic trauma casemix and aerosol-generating anaesthetic procedures due to social distancing/lockdown. Mortality rates and survival probabilities were also hypothesised to differ due to the first COVID-19 outbreak.

For peer review only

Methods

Study design: This is the first multi-centre longitudinal observational study observing patients who were acutely referred to the trauma and orthopaedic departments as well as those operated on within the same 6-week interval comparing 2019 to 2020.

Setting: Seven principal hospitals contributed data from 6 major urban cities including London, Gateshead, Middlesbrough, Dartford, Newport, and Reading.

Patient sampling: All acute referrals, operative notes, inpatient medical records and discharge summaries were accessed using electronic medical system at each contributing hospital trust.

Study period: The 6-week study period was from the start of social distancing on Tuesday 17th March 2020 to Tuesday 28th April 2020 which encompassed the national lockdown measures instigated on the 23rd March 2020. This period was considered the peak 6 weeks of the epidemic in the UK as outlined by the recorded mortality rates and R-values published by the Office of National Statistics.[14] This time period was compared to the same 6-week interval from Tuesday 19th March to Tuesday 30th April 2019 (i.e. prior to any COVID-19 related measures) to compare the impact of the pandemic one year apart.

Outcomes/objectives: Primary outcomes included the number of acute trauma referrals and those undergoing operative intervention, post-operative complications, mortality rates, and the proportion of patients contracting COVID-19. Secondary outcomes consisted of the

For peer review only - http://bmjopen.bmj.com/site/about/guidelines.xhtml

mechanism of injury, type of operative intervention and proportion of aerosolising-generating anaesthesia utilised.

Inclusion criteria: All acute orthopaedic trauma referrals presenting to the Emergency Department during the intervals one year apart were included. All orthopaedic trauma cases that required an operation, including those from acute orthopaedic trauma referrals, within the intervals one year apart. Those patients listed for an operation due to orthopaedic trauma prior to time period of data collection were included in the final analysis. We adhered to STROBE guidelines for observational studies.

Exclusion criteria: Any cases being referred internally from other specialties for trauma and orthopaedic advice and input, as well as referrals from any external centre asking for tertiary advice were excluded from further analysis. Any patients with post-operative complications arising from the period prior to the data collection were excluded. For operative trauma cases, those undergoing spinal procedures were excluded as these are jointly treated by Neurosurgery in most hospitals. All non-urgent semi-elective procedures were excluded from analysis as well, as they would inaccurately assess the impact of any social distancing measures on the trauma workload. Routine elective orthopaedic cases were excluded.

Data points: Demographics including age, sex and ASA grades were recorded for all patients. Injury characteristics were recorded, including the anatomical location and if the injury was open or closed. The mechanism of injury was categorised and whether the patient was referred as a trauma call. The nature of the operative procedures and the anaesthetic techniques were recorded. Patients undergoing multiple procedures were recorded for every

For peer review only - http://bmjopen.bmj.com/site/about/guidelines.xhtml

episode where they were taken to theatre. 6-week mortality rate was recorded as well as the COVID-19 status of any symptomatic patients or suspected cases. Data points were divided into acute referrals and operative casemix as seen in table 1.

Table 1: data points for acute referrals and operative casemix

Age (years)	Gender (Male / female by birth)	ASA (1-5)	Date of injury / presentation
Injury	Mechanism of injury	Open Vs Closed fracture	Trauma Call (Yes / No)
Operative procedure	Anaesthetic Technique (AGP vs non-AGP)	Seniority of Surgeon (Consultants vs trainees)	Comorbidities
6-week mortality	Post-op complications	Surgery time since admission (hours)	COVID status (from PCR swabs)

- *Anaesthetic techniques:* This was divided into anaesthetic aerosolising-generating procedures (AGP) which consisted of any intubation (including laryngeal mask airway and endotracheal intubation) for a general anaesthetic. All other anaesthetic techniques including regional and local anaesthetics were deemed as non-AGPs.
- *COVID status*: At the time, COVID was being diagnosed with polymerase chain reaction (PCR) from nasal and oropharyngeal swabs with a duration of 1 to 4 days where the sample was tested both locally in the hospital lab as well as corroborated with national lab testing to reduce risk of unequivocacy. Groups of patients were divided into either not swabbed (due to being asymptomatic) or swabbed due to presence of documented symptoms which yielded either negative or positive results.

For peer review only

For peer review only - http://bmjopen.bmj.com/site/about/guidelines.xhtml

168

169 *Statistical analysis:* All the data were recorded, anonymised and verified by four members of the study group for their accuracy. The data were processed using Microsoft Excel (Microsoft, Washington, USA). Shapiro-Wilk test indicated a normal distribution for age and days to discharge from hospital; hence, the mean (± standard deviation; 95% CI) were calculated for both. ASA did not follow normality and was analysed using median (±median absolute deviation [MAD]) and interquartile range (IQR). Both prevalence or risk and odds ratios were calculated as well as a Fisher's exact test for statistical significance for categorical data, defined as $p \leq 0.05$. Percentages and confidence intervals were rounded off to one decimal place.

178

179 *The collaborative*: The COVid Emergency Related Trauma and orthopaedics (COVERT) Collaborative was founded at Imperial College Healthcare NHS Trust. It is currently a member of the COVID Research Group and it has been endorsed by the Royal College of Surgeons of England and Imperial College Healthcare NHS Trust.

183

184 *Patient and Public Involvement:* Patients and the public were not involved in the study design, recruitment or conduct.

For peer review only - http://bmjopen.bmj.com/site/about/guidelines.xhtml

Results

A total of 4840 clinical encounters took place between the study periods. A total of 172 spinal operations and presentations were excluded from the final analysis. Table 2 outlined demographic data. During the COVID-19 period there was a 34% reduction in acute orthopaedic trauma referrals compared to 2019 (1792 down to 1183 referrals), and 29.5% less surgical interventions (993 down to 700 operations). Figures 1-3 have categorised these clinical encounters into types and mechanisms of injury for both acute referrals and operative cases between both years respectively.

Table 2: Demographic data of pre- and post-COVID

		Pre-COVID (2019)		COVID (2020)	
	**Total**	**1792**		**1183**	
**Acute referrals**	*Male*	935	52.2%	560	47.3%
	Female	857	47.8%	623	52.7%
	Mean Age ± SD (95% CI)	52.2 ± 27.9 (50.9 - 53.5)		55.8 ± 27.9 (54.3 - 57.4)	
**Operative cases**	**Total**	**993**		**700**	
	Male	499	50.3%	320	45.7%
	Female	494	49.7%	380	54.3%
	Mean Age ± SD (95% CI)	51.7 ± 28.1 (50 - 53.5)		57.7 ± 26.7 (55.7 - 59.6)	
	Median ASA ± MAD (IQR)	2 ± 1 (2)		2 ± 1 (2)	

Figure 1: Types and mechanisms of injuries for acute referrals

Figure 2: Types and mechanisms of injuries for operative cases

Figure 3: operative and anaesthetic techniques compared between pre- and post-COVID

For peer review only

For peer review only - http://bmjopen.bmj.com/site/about/guidelines.xhtml

COVID status

COVID status for both acute referrals and operative casemix including results in all mortalities were demonstrated in figure 4. Mortalities with positive swab results were confirmed prior to the event of death within 6-weeks post-presentation. Approximately three-quarters were not tested and a fifth had negative results. 0.9% of the acute referrals resulted in deaths and 28.9% of those mortalities tested positive for COVID. Furthermore, post-operative mortalities represented 1.6% of the entire operative casemix, and 32.4% of those mortalities had a confirmed positive COVID-19 diagnosis prior to their death.

Figure 4: COVID status for acute referrals and operative cases as a measure of proportions

Risk (or prevalence) and odds ratios

Table 3: Risk (or prevalence) and odds ratios for acute referrals and operative caseloads.

Comparisons are made between COVID period against the pre-COVID period. Value >1

indicated greater odds or risk during the COVID period.

		Acute referrals			Operative caseload		
		RR	OR	p-value	RR	OR	p-value
	Mortality	2.50	2.55	0.0005	2.19	2.25	0.004
	Mortality due to COVID-related complications vs non-COVID causes	14.2	19.7	0.004	15.1	22.0	0.004

For peer review only

**Morbidity & Mortality**	Peri-/post-operative complications including COVID	[redacted]	[redacted]	[redacted]	5.88	6.09	0.00001
	Peri-/post-operative complications excluding COVID	[redacted]	[redacted]	[redacted]	3.65	3.72	0.003
	Peri-/post-operative COVID positive testing	[redacted]	[redacted]	[redacted]	32.6	23.4	0.0009
**Anaesthetic technique**	General anaesthetic only	[redacted]	[redacted]	[redacted]	1.22	1.61	0.00001
	General anaesthetic ± block	[redacted]	[redacted]	[redacted]	1.23	1.75	0.00001
**Consultant involvement**	Consultant-led operation	[redacted]	[redacted]	[redacted]	1.36	2.08	0.00001
**Operation technique**	Open reduction + internal fixation	[redacted]	[redacted]	[redacted]	0.81	0.74	0.007
	Dynamic hip screw	[redacted]	[redacted]	[redacted]	2.02	2.11	0.00001
	Removal of metal/foreign body	[redacted]	[redacted]	[redacted]	0.24	0.23	0.003
**Mechanism of injury**	Road traffic accident	0.58	0.56	0.001	0.45	0.43	0.00001
	Fall (<1.5m)	1.19	1.54	0.00001	1.17	1.49	0.0001
	Sporting injury	0.63	0.60	0.0005	0.64	0.61	0.003
	Infection	0.69	0.66	0.001	1.70	1.77	0.005
	Trauma call	[redacted]	[redacted]	[redacted]	0.55	0.52	0.0005
**Type of injury**	Neck of femur (NOF) fracture	1.44	1.57	0.00001	1.51	1.79	0.00001
	Lower limb (excl. NOF)	0.89	0.84	0.04	0.74	0.65	0.0001
**Gender**	Male	0.91	0.82	0.01	[redacted]	[redacted]	[redacted]

For peer review only

Table 3 outlined the risk [RR] (or prevalence [PR]) and odds ratios [OR] alongside their 95% confidence intervals and statistical significance. The risk ratio is synonymous with the prevalence ratio. Only those factors that were statistically significant within the acute referrals and operative caseloads were included. There were trends demonstrating increase in mortality rates, use of anaesthetic AGPs, Consultant-led operations, hip fracture surgery and falls; but a decrease in other lower limb operations, open reduction and internal fixation, removal of metalwork and foreign bodies, road traffic accidents, sporting injuries and infection.

Mortality

Table 3 indicated that the 6-week mortality rate more than doubled significantly for both risk (RR=2.19-2.50) and odds (OR=2.25-2.55) ratios during the COVID period. COVID-related complications were still responsible for increasing the odds of mortality by 20 to 22 times within all mortalities from both acute referrals and operative cases (as compared to non-COVID causes for all mortality in the year 2019). Table 4 confirmed that the mean age of mortalities across the board were in the elderly patient population with a high median ASA grade. Males were consistently in the minority, while neck of femur fracture was the modal diagnosis due to falls and persistently in the majority, followed by lower limb injuries (figures 5-6). At least 82% of operations were related to neck of femur fractures in which half of all operations during the COVID period involved anaesthetic AGPs. Whereas the mortalities from pre-COVID operations did not have Consultant-led (as primary surgeon) surgery, that increased to three-fifths of all operations conducted during the COVID period (figure 7).

Table 4: Patient demographics, date of injuries, and time to mortality

For peer review only

	Acute referrals		*Operative casemix*	
	**2019 (n=23)**	**2020 (n=38)**	**2019 (n=22)**	**2020 (n=34)**
Mortality	1.3%	3.2%	2.2%	4.9%
Mortality with COVID positive PCR result		0.9% (total)<br>28.0% (mortality cohort)		1.6% (total)<br>32.9% (mortality cohort)
Post-op morbidity			0.7%	4.1%
Age (years; mean±SD; 95% CI)	80.2 ± 16.4<br>(73.2 - 87.2)	77 ± 23<br>(67 - 88)	83.9±12.2<br>(78.7 - 89.1)	84.0±13.5<br>(79.4 - 88.5)
Male	9 39%	16 42%	8 36%	15 44%
ASA (median±MAD; IQR)			3 ± 0<br>(1)	3 ± 0<br>(0)
Date of injury (mean days±SD; 95%CI)	6/4 ± 11<br>(1/4 - 10/4)	31/3 ± 12<br>(26/3 - 5/4)	6/4 ±12<br>(1/4 - 11/4)	30/3 ±14<br>(25/3 - 4/4)
Time from admission to mortality (mean days±SD; 95%CI)	10.3 ± 7.5<br>(7.1 - 13.5)	11 ± 10<br>(7 - 15)	14.3 ± 10.4<br>(9.8 - 18.7)	13.8 ± 10.4<br>(10.2 - 17.3)

Figure 5: types and mechanism of injury for mortalities in acute referral cohorts

Figure 6: types and mechanism of injury for mortalities in operative casemix cohorts

Figure 7: Surgical and anaesthetic techniques utilised in mortalities as a means of proportions

Sub-group analysis for neck of femur fracture

A subgroup analysis of hip fractures was conducted due to its recognised risk of mortality within orthopaedic trauma. Those who were operated on in 2020, 20.2% tested positive for COVID, 47.3% tested negative and the remaining 32.4% were not tested due to being asymptomatic. Furthermore, 82.3% of all mortalities in 2020 sustained a neck of femur

For peer review only - http://bmjopen.bmj.com/site/about/guidelines.xhtml

fracture in which only 35.7% of this cohort had a positive swab result, 21.4% with negative swab results and the remaining 42.9% were not swabbed due to being asymptomatic. There was no statistical difference in the odds and risk ratios between both years for mortality rate in NOFF (table 3). The absolute numbers did not change much, but because of a drop in other presentations, the relative percentage of NOFF markedly rose. Hence, the mortality expressed as a percentage of cases is notably higher for all operations, and not necessarily if stripped down to hip fractures alone.

Pre- and post-operative morbidity

Taking into account that COVID was a peri-operative complication since patients may have been symptomatic with COVID manifestations pre-operatively but only had the swab results return with a positive finding either pre- or post-operatively; the commonest post-operative complication in the COVID period was a hospital-acquired pneumonia but with negative COVID swab results or the decision not to test at all. The second most common post-operative complication in the year 2020 was extra-pulmonary sepsis (Appendix 1). The proportion of post-operative complications had significantly increased when including or excluding COVID as a peri- or post-operative complication in 2020 (0.70% vs. 2.57-4.14%; p=0.003) with varying odds (3.72-23.4) and risk (3.65-32.6) ratios (table 3). Appendices 2-3 focused on the total number and nature of comorbidities within the mortality groups. Multiple contingency chi-square test was insignificant for both number of comorbidities and individual comorbidities between both years, except for cardiovascular and cerebrovascular disease in acute referrals.

Appendix 1: Post-operative complications for both years

Appendix 2: Type of comorbidities for all mortalities in both years

For peer review only - http://bmjopen.bmj.com/site/about/guidelines.xhtml

Appendix 3: number of comorbidities for all mortalities in both years

Survival probability

6-week Kaplan-Meier survival probability analysis for mortalities between both years were plotted in figures 8-9. There were similar patterns of survival probability between both cohorts (i.e. 2019 vs 2020 cumulative). However, the lowest survival probability and the shortest timeframe were observed in the confirmed COVID positive cohorts (figure 8). 8 (72.7%) patients had femoral trauma, most being neck of femur fractures, distal femur fracture and a dislocated hip hemiarthroplasty post-fracture. Unexpectedly, there was a reversal of trends observed for the 6-week Kaplan-Meier survival analysis once admitted and operated on in figure 9. Mortalities within the pre-COVID period had the lowest survival probability compared to the post-COVID cohort. The COVID positive mortalities were observed to have the highest survival probability 11 days prior to converging with those mortalities without COVID symptoms.

Figure 8: Six-week Kaplan-Meier survival probability analysis for mortalities between pre- and post-COVID for acutely referred from the Emergency Department

Figure 9: Six-week Kaplan-Meier survival probability analysis for mortalities between pre- and post-COVID for those undergoing surgery

For peer review only - http://bmjopen.bmj.com/site/about/guidelines.xhtml

Discussion

Comment on alternative hypothesis

There was a significant difference between pre- and post-COVID periods at its peak. The alternative hypothesis was not rejected with respect to prevalence of (i) acute orthopaedic trauma referrals (reduced by 34%), (ii) surgical interventions (reduced by 29.5%), (iii) anaesthetic aerosolising-generating procedures, (iv) 6-week mortality rates (more than doubled in the COVID period), and (v) survival probability between pre- and post-COVID eras.

Corroboration of our results with current literature

The 34% reduction in acute trauma referrals is in keeping with previous single centre studies performed in the UK with results ranging between 26-59%.[10-13,15,16] As described in these previous studies we would attribute the overall reduction of trauma workload to be due to reduction in travel and outdoor activities during the national lockdown. MacDonald *et al*.[17] described a similar effect in their multi-centre study with a reduction of operative workload by 26.5% compared to 29.5% in our study. Sites recruited for this study confirmed that they continued to operate at their own facilities during the data collection period whereas some later used alternative and external facilities including private hospitals through NHS England pathways (as mentioned by Dayananda et al.[18]), which may have impacted nosocomial rates of COVID, morbidity and mortality. However, this would be difficult to assess since it would also depend on the diversions of the ambulance services to 'clean' versus 'contaminated' hospital sites.

Changes in trends during the peak of COVID

For peer review only - http://bmjopen.bmj.com/site/about/guidelines.xhtml

With respect to the operative caseload (table 3), patients had half (OR=0.52, p<0.001) the odds of presenting as a trauma call. This was due to the odds ratios of road traffic accidents, sporting injuries, infection, and lower limb injuries were significantly less (by 34-44%; OR=0.56-0.66, p<0.01) during the COVID period. Conversely, there was a significant rise in the odds of neck of femur fractures, falls, the use of anaesthetic AGP and Consultant-led operations; a finding also reflected by Arafa *et al.*[19]

Although the expectation was to minimise the use of aerosolising-generating anaesthetic procedures, there was in fact an increased prevalence of using general anaesthesia ± block up to an odds of 75%, in order to create a 'closed circuit' for the airways. As the anaesthetic methods was not well documented in the pre-COVID era in a fifth (21.3%) of cases, this skewed the data as it may have been difficult to extract that data from 2019. The odds of a Consultant-led operation doubled (OR=2.08) during the COVID period as a consequence of all elective operations being suspended, hence more Consultants were relocated to trauma theatre and increased pressure within the theatre environments led to Consultant-delivered, rather than Consultant-led care. With respect to surgical procedures, there was a significant reduction in prevalence ratio of open reduction and internal fixation by a fifth (PR=0.81) and removal of metalwork and foreign bodies by three-quarters (PR=0.24), while there was a doubling (PR=2.02) in dynamic hip screw fixation in the COVID era.

Mortality and Morbidity

Mortality during COVID-19 timeframe

Comparatively, the COVIDSurg Collaborative observed a mortality rate of 28.8% (p<0.0001) of orthopaedic patients who underwent surgery (both elective and trauma) within the first quarter of the year.[20] The increased mortality during the pandemic is partly due to selection

For peer review only - http://bmjopen.bmj.com/site/about/guidelines.xhtml

of cases that required surgical intervention. The decrease in acute referrals and operations indicated a higher threshold for treatment (due to a redistribution of hospital resources during the pandemic). However, no such case was denied surgery but in the worst-case scenario patients were offered postponed treatment which is acceptable practice (i.e. within 2 weeks).

Role of morbidity in mortality during COVID-19

Results from figures 5-7 and appendices 1-3 were corroborated with the COVIDSurg publication[20] which confirmed a significant association of mortality with myocardial infarction and congestive heart failure. However, hypertension and stroke/transient ischemic attacks were not significantly associated. In our study, all cardiovascular diseases (including peripheral vascular, arrhythmias, hypertension, heart failure, myocardial infarction and acute coronary syndromes) were combined with cerebrovascular diseases (consisting of strokes and transient ischemic attacks). Unlike their study, our study did not find a significant association with chronic kidney disease, chronic obstructive disease (which included asthma) and dementia in all mortalities during the 2020 timeframe regardless of the COVID status. The differences may stem from that their study looked at the comparison of mortality rates within the same cohort during the COVID era, whereas this study is sub-analysing the entire mortality cohort on its own to observe for specific associations and risks.

For peer review only

Survival probability between both years

As expected, reduced survival probability reflected the most vulnerable patient profiles, usually with multiple pre- and post-operative comorbidities (appendix 1-3). A reason for a transient increase, and unexpected reversal, in 6-week survival probability in the operative COVID cohort (figures 8-9) may be explained by dedicated wards being ring-fenced to host confirmed COVID positive patients with a heightened care of nursing, medical cover and

personal protective equipment. Prior to the onset of a possible vaccination, symptomatic management and shielding were the mainstay treatments for COVID positive patients. None of these patients were stepped up to the Intensive Treatment Unit due to being categorised as high-risk stratification for mortality based on age and extent of comorbidities.

Justification of conducting this study

As lockdown measures in the UK and globally eases and the incidence of trauma returns to pre-lockdown trends, it is imperative that we understand the true increased risk of mortality in acute trauma during the COVID-19 era. A recent publication by Kader et al.[21] has suggested that the rate of mortality from COVID-19 for elective orthopaedic patients is low; yet this is the first British multi-centre study to quantify mortality risk for trauma patients. Trauma procedures due to the nature of the injuries are necessary and time-critical, and nobody can afford to postpone trauma care even during a global pandemic.[22]

Furthermore, the Corona Hands Collaborative[23] published that upper limb trauma patients had SARS-CoV-2 complication rate of 0.18% (n=2) with 0.09% (n=1) overall mortality at the peak of the first wave in April 2020. However, their collaborative looked into a shorter post-operative period (30 vs 42 days) but they agreed that patients who had been hospitalised for a prolonged period before their surgery were at increased risk of both COVID-related and post-operative complications. Most of their patient cohort, who were both younger and fitter than our cohorts, would be classified as the 'walking wounded' and could usually be managed as day-case procedures.

Although the trends in mechanisms of injury in our study were reflective of those within a US multi-centre study, there was an opposing trend in the number medical/surgical

For peer review only - http://bmjopen.bmj.com/site/about/guidelines.xhtml

procedures.[24] That could be due to their study encompassing level 1 trauma centres with a mean younger patient population. However, we do agree that with time and from experiential learning, hospitals improved their coping strategies with the pandemic and enhanced patient safety by enforcing personal protection equipment, hosting dedicated theatres for COVID-positive patients, separating sites as clean and contaminated, ringfencing COVID-positive patients to dedicated wards, and promoting routine COVID PCR swabs for all admissions and pre-operative checklists.

With an overall mortality risk in 2020 doubled that of 2019, clinicians need to counsel patients presenting with acute orthopaedic trauma of the increased risk in the COVID-19 era, especially for those identified as increased risk stratification with multiple underlying comorbidities, elderly and frailty. With the ongoing risk of a subsequent wave and resurgence of COVID-19 cases on top of the inevitable winter pressures, this data is of critical importance in the risk management, decision-making and policymaking of trauma patients both in the UK and across the globe.

Neck of femur fractures

Since the aetiology of neck of femur fracture is often low energy falls in the home environment, it is not unexpected to observe a consistency of neck of femur fractures in the elderly and the vulnerable during lockdown as seen in figures 1-2. Those with neck of femur fractures remain at greatest risk of mortality and there have been further studies evaluating the risk of COVID-19 on this inherently high-risk cohort.[25-28] COVID-19 itself has been identified as an independent risk factor in increasing mortality in neck of femur fractures.[29,30]

For peer review only - http://bmjopen.bmj.com/site/about/guidelines.xhtml

The increased mortality reflect the increased proportion of NOFF patients that have a higher baseline mortality which has been echoed by the Scottish IMPACT-Restart study.[28] There are several justifications such as reduced help, lack of assistance and staff shortages due to the effect of the national lockdown which required elderly patients to be more independent, unsupervised and at higher risk of falling. Nevertheless, it should be considered that odds of falls may have increased due to prodromal symptoms and clinical manifestations of COVID.

If these 'at risk' patients were symptomatic with the virus, then aggressive pre-operative optimisation would occur. Since 91% (n=10) of COVID positive patients had sustained a neck of femur fracture, the National Hip Fracture Database best practice tariff of operating within an ideal 36-hour window set by the Royal College of Physicians was suspended until the patient was stabilised. All hip fracture patients in this cohort were operated on and had dedicated orthogeriatric input commencing from hospital admission. Hence the early peri-operative period and surgery encompassed within the 10-day period post-admission. Moreover, neck of femur fractures are recognised as a pre-terminal illness and are known to carry a high risk of mortality in the first month which is trebled in the first year after the injury.[31]

Strengths and weaknesses of the study and in relation to other studies

This was the first representative observational multi-centre study of the UK looking into the impact of COVID-19 pandemic on general trauma and orthopaedic surgical specialty. Studies thus far have only shed light on local scales, cross-speciality, reflecting a fraction of our study population or contain 30-day mortality at most.[10-13,20,30,32,33] Weaknesses included loss of data points which have been accounted for in the tables (i.e. labelled as unknown). However this did not affect the final analysis of data points (table 1). Operations conducted

For peer review only

For peer review only - http://bmjopen.bmj.com/site/about/guidelines.xhtml

outside the specific study periods will not account for all those operations required such as for NOFF. It does not suggest that the number of NOFF not accounted for have been managed conservatively (as discovered by Cherevu et al.[34]), since some NOFFs may have breached time to surgery due to medical reasons or being influenced by international guidelines.[35]

Limitations and future research

It is vital to continue exploring the impact of the pandemic on a larger scale. Ideally, more secondary care providers consisting of district general hospitals and major trauma centres will submit data. The diagnosis of COVID was dependent on positive PCR swabs for this study rather than non-specific changes seen on chest CT or plain radiographs. This does not account for false negatives with clinical respiratory symptomatology or true positives in those asymptomatic. Nevertheless, this issue with data has been speculated on in another national study.[23] Data ought to be submitted during the peak of the pandemic as well as at various time intervals as the lockdown measures ease resulting in more freedom of movement while also accounting for the continued risk of subsequent waves and national lockdowns.[36] Further studies will also require to compare the impact of the pandemic on the speciality in the UK compared to other countries on other continents.

For peer review only - http://bmjopen.bmj.com/site/about/guidelines.xhtml

Conclusion

This was the first, longest and largest British multi-centre representation of the impact of COVID-19 pandemic on acute orthopaedic trauma referrals and mortality between mid-March to end-April, representing the peak of the first wave during the lockdown. The mortality rate for acute referrals, as well as those undergoing operative intervention, more than doubled in odds when compared to the same time interval one year ago. The majority of mortalities consisted of the elderly with neck of femur fractures and cardiovascular and/or cerebrovascular diseases. This study will aid clinicians in counselling trauma patients of the increased risk of mortality during the era of COVID-19 and also aid in both healthcare infrastructure, resource allocation, decision-making and policymaking as we continue to battle with the pandemic.

For peer review only - http://bmjopen.bmj.com/site/about/guidelines.xhtml

Research Ethics Approval - Human Participants: This study involves human participants but an Ethics Committee(s) or Institutional Board(s) exempted this study. All data points were utilised for routine auditing purposes to reflect departmental activity and service provision without altering clinical care pathways. Each centre contributing data to this study registered their interests with local authority and the auditing or clinical governance departments. No informed consent was required as there was no identifiable data. All data were anonymised at the time of collection and submission. Each patient was assigned a unique identification number which was cross-referenced with the patients' individual hospital identification or medical record numbers. This cross-referenced list remained internally within the hospital trust computer server handled by the contributing team from each trust. The data was transferred and stored using the NHS.net email server which has been approved for transfer of patient data. Data protection compliance was abided by at all times. The lead centre was Imperial College Healthcare NHS Trust where this study was first approved as a clinical audit prior to expanding onto a national scale. All centres gave permission for the use of their data. This study was assessed using the UKRI/MRC/NHS Health Research Authority Ethics Decision Tool and was considered an 'audit/not research'; and therefore it was not subject to further ethical review by the NHS Research Ethics Committee (NHS REC).

For peer review only

Competing Interests: None declared

Funding: Nil

Data availability statement: Underlying data, code and supporting documentation may be made available as a redacted version to interested parties, subject to the completion of a protocol and signing of a Data Transfer Agreement.

Acknowledgements: none

For peer review only

References

1. World Health Organization (a). WHO Coronavirus Disease (COVID-19) Dashboard. Available online at https://covid19.who.int. Cited 2021 Sep 12.
2. Wu F, Zhao S, Yu B, Chen YM, Wang W, Song ZG, et al. A new coronavirus associated with human respiratory disease in China. *Nature.* 2020;579:265-9.
3. World Health Organization. Coronavirus disease 2019 (COVID-19): situation report, 66. 26 March 2020. https://www.who.int/docs/default-source/coronaviruse/situation-reports/20200326-sitrep-66-covid-19.pdf?sfvrsn=9e5b8b48_2. Cited 2021 Aug 30.
4. Spiteri G, Fielding J, Diercke M, Campese C, Enouf V, Gaymard A, et al. First cases of coronavirus disease 2019 (COVID-19) in the WHO European Region, 24 January to 21 February 2020. Eurosurveillance 2020 (25): 2000178.
5. UK Government (a) (2020) Oral statement to Parliament, Controlling the spread of COVID-19: Health Secretary's statement to Parliament. 16 Mar 2020. www.gov.uk/government/speeches/controlling-the-spread-of-covid-19-health-secretarys-statement-to-parliament. Cited 2021 Aug 25.
6. UK Government (b) (2020) Guidance: Oral statement to and away from others (social distancing). 23 Mar 2020. https://www.gov.uk/government/publications/full-guidance-on-staying-at-home-and-away-from-others. Cited 2021 Sept 5.
7. NHS England (2020a) Next steps on NHS response to COVID-19: Letter from Sir Simon Stevens and Amanda Pritchard. 17 Mar 2020. www.england.nhs.uk/coronavirus/publication/next-steps-on-nhs-response-to-covid-19-letter-from-simon-stevens-and-amanda-pritchard. Cited 2021 Aug 27.
8. Royal College of Surgeons. Guidance for surgeons working during the COVID-19 pandemic from the Surgical Royal Colleges of the United Kingdom and Ireland. March

For peer review only

For peer review only - http://bmjopen.bmj.com/site/about/guidelines.xhtml

20, 2020. www.rcseng.ac.uk/coronavirus/joint-guidance-for-surgeons-v1. Cited 2021 Aug 28.

9. British Orthopaedic Association. British Orthopaedic Association for Standards of Trauma (BOAST). Management of patients with urgent orthopaedic conditions and trauma during the coronavirus pandemic. March 24, 2020. www.boa.ac.uk/resources/statement-for-boa-members-on-trauma-and-orthopaedic-care-in-the-uk-during-coronavirus-pandemic.html. Cited 2021 Aug 28.

10. Park C, Sugand K, Nathwani D, Bhattacharya R, Sarraf KM. Impact of the COVID-19 pandemic on orthopaedic trauma workload in a London level 1 trauma centre: the "golden month". Acta Orthop. 2020 Jun 23;1-6.

11. Murphy T, Akehurst H, Mutimer J. Impact of the 2020 COVID-19 pandemic on the workload of the orthopaedic service in a busy UK district general hospital. Injury 2020;S0020-1383(20)30560-X.

12. Elhalawany A, Beastall J, Cousins G. The impact of the COVID-19 lockdown on orthopaedic emergency presentations in a remote and rural population, Bone Joint Open. 2020;1(10): 621-627.

13. Karia M, Gupta V, Zahra W, et al. The effect of COVID-19 on the trauma burden, theatre efficiency and training opportunities in a district general hospital: planning for a future outbreak, Bone Joint Open. 2020;1-8:494–499.

14. Office of National Statistics. Deaths registered weekly in England and Wales, provisional: 27 Aug, 2021. Released 7 Sept, 2021. www.ons.gov.uk/peoplepopulationandcommunity/birthsdeathsandmarriages/deaths/bullet

For peer review only - http://bmjopen.bmj.com/site/about/guidelines.xhtml

ins/deathsregisteredweeklyinenglandandwalesprovisional/27august2021. Cited 2021 Sep 12.

15. Atia F, Pocnetz S, Selby A, et al. The effect of the COVID-19 lockdown on hand trauma surgery utilization. *Bone Jt Open.* 2020;1(10):639-643.

16. Esteban PL, Querolt Coll J, Xicola Martínez M, et al. Has COVID-19 affected the number and severity of visits to a traumatology emergency department? *Bone Jt Open.* 2020;1(10):617-620.

17. MacDonald DRW, Neilly DW, Davies PSE, et al; SCORE authors. Effects of the COVID-19 lockdown on orthopaedic trauma: a multicentre study across Scotland. *Bone Jt Open*. 2020;1(9):541-548.

18. Dayananda KSS, Mercer ST, Agarwal R, et al. A comparative review of 1,004 orthopaedic trauma patients before and during the COVID-19 pandemic. Bone Jt Open. 2020;1(8):568-575.

19. Arafa M, Nesar S, Abu-Jabeh H, et al. COVID-19 pandemic and hip fractures: impact and lessons learned, Bone Joint Open. 2020;1-9:530–540.

20. COVIDSurg Collaborative. Mortality and pulmonary complications in patients undergoing surgery with perioperative SARS-CoV-2 infection: an international cohort study. *Lancet*. 2020 May 29.

21. Kader N. Clement ND. Patel VR. Caplan N. Banaszkiewicz P. Kader D. The theoretical mortality risk of an asymptomatic patient with a negative SARS-CoV-2 test developing COVID-19 following elective orthopaedic surgery. *Bone Joint J.* 2020;1-5.

For peer review only

22. Service BC, Collins AP, Crespo A, Couto P, Gupta S, Avilucea F, et al. Medically necessary orthopaedic surgery during the COVID-19 pandemic: safe surgical practices and a classification to guide treatment. *JBJS*. 2020;102(14):e76.

23. Dean BJF; Corona Hands Collaborative. Mortality and pulmonary complications in patients undergoing upper extremity surgery at the peak of the SARS-CoV-2 pandemic in the UK: a national cohort study. BMJ Qual Saf. 2020:bmjqs-2020-012156.

24. Salottolo K, Caiafa R, Mueller J, et al. Multicenter study of US trauma centres examining the effect of the COVID-19 pandemic on injury causes, diagnoses and procedures. *Trauma Surgery & Acute Care Open*. 2021;6:e000655.

25. Kayani B, Onochie E, Patil V, Begum F, Cuthbert R, Ferguson D, et al. The effects of COVID-19 on perioperative morbidity and mortality in patients with hip fractures. *Bone Joint J.* 2020;1-10.

26. Hall AJ, Clement ND, Farrow L, IMPACT-Scot Study Group, MacLullich AMJ, Dall GF, et al. IMPACT-Scot report on COVID-19 and hip fractures. *Bone Joint J.* 2020;1-10.

27. Clement ND, Hall AJ, Makaram NS, et al. IMPACT-Restart: the influence of COVID-19 on postoperative mortality and risk factors associated with SARS-CoV-2 infection after orthopaedic and trauma surgery. *Bone Joint J.* 2020;102-B(12):1774-1781.

For peer review only - http://bmjopen.bmj.com/site/about/guidelines.xhtml

28. Mackay N, Wilding C, Langley C, et al. The impact of COVID-19 on trauma and orthopaedic patients requiring surgery during the peak of the pandemic. *Bone Jt Open.* 2020;1-9:520–529.

29. Narang A, Chan G, Aframian A, et al. Thirty-day mortality following surgical management of hip fractures during the COVID-19 pandemic: findings from a prospective multi-centre UK study. *Int Orthop*. 2021;45(1):23-31.

30. Karayiannis PN, Roberts V, Cassidy R, Mayne AIW, McAuley D, Milligan DJ, Diamond O. 30-day mortality following trauma and orthopaedic surgery during the peak of the COVID-19 pandemic: a multicentre regional analysis of 484 patients. Bone Jt Open. 2020;1(7):392-397.

31. British Orthopaedic Association. British Orthopaedic Association Standards for Trauma. January 2012. BOAST 1 Version 2 - Patients sustaining a Fragility Hip Fracture. www.boa.ac.uk/uploads/assets/6750e0bf-4aa3-4680-b1612265704512db/patients%20sustaining%20a%20fragility%20hip%20fracture.pdf. Cited 2021 Aug 25.

32. Greenhalgh M, Dupley L, Unsworth R, Boden R. Where did all the trauma go? A rapid review of the demands on orthopaedic services at a UK Major Trauma Centre during the COVID-19 pandemic. Int J Clin Pract. 2021;75(3):e13690.

For peer review only - http://bmjopen.bmj.com/site/about/guidelines.xhtml

33. Balakumar B, Nandra RS, Woffenden H, Atkin B, Mahmood A, Cooper G, Cooper J, Hindle P. Mortality risk of surgically managing orthopaedic trauma during the COVID-19 pandemic. Bone Jt Open. 2021;2(5):330-336.

34. Cheruvu MS, Bhachu DS, Mulrain J, et al. Effect of COVID-19 on a rural orthopaedic hip fracture service. Bone Jt Open. 2020;1(8):500-507.

35. Lewis PM, Waddell JP. When is the ideal time to operate on a patient with a fracture of the hip? : a review of the available literature. Bone Joint J. 2016;98-B(12):1573-1581.

36. Adebowale V, Alderson D, Burn W, Dickson J, Godlee F, Goddard A, et al. Covid-19: Call for a rapid forward looking review of the UK's preparedness for a second wave-an open letter to the leaders of all UK political parties [published correction appears in BMJ. 2020 Jul 16;370:m2866. Dixon, Jeanette [corrected to Dickson, Jeanette]]. *BMJ.* 2020;369:m2514. Published 2020 Jun 23.

For peer review only

Contributorship statement according to the ICMJE guidelines

We have read the ICJME guidelines attentively and have outlined the collaborative contributors below who have all satisfied the criteria to be recognised as a collaborative co-author if this study is published. The core committee of the collaborative consists of KS, AA, CP and KMS who all conceptualised the study, led the planning/investigation/methodology/design, supervised the collaborative contributors' roles, as well as the initial and final version of the manuscript. Additionally, KS (primary author) was also leading data curation, project administration and resource allocation with KMS. KS was also leading on validation. KS, CP and AA led the data analysis and the reporting of the results. All other collaborative members, from the seven centres and outside the core committee, were involved in data curation, formal analysis and in resource allocation internally under consultant supervision (i.e. those with FRCS). Individual contributions from the core committee and every collaborative member has been outlined below:

COVERT Collaborative members, their affiliations and extent of contribution

Core Committee

Name	Qualifi-cation	Contribution	E-mail

For peer review only - http://bmjopen.bmj.com/site/about/guidelines.xhtml

1	**Kapil Sugand** Primary & corresponding author *58 Thames Avenue, Greenford, Middlesex, UB6 8JN* *E: ks704@ic.ac.uk* *T: 00447773642813*	MRCS	• Conceptualisation<br>• Data curation<br>• Formal analysis<br>• Investigation<br>• Methodology/Design<br>• Planning<br>• Project administration<br>• Resources<br>• Supervise<br>• Validation<br>• Data analysis<br>• Visualisation<br>• Writing original draft<br>• Writing – reviewing and editing	Ks704@ic.ac.uk<br><br>ORCID: 0000-0003-3185-954X
2	Arash Aframian	MRCS	• Conceptualisation<br>• Formal analysis<br>• Investigation<br>• Methodology/Design<br>• Planning<br>• Supervise<br>• Data analysis<br>• Writing – reviewing and editing	arash.aframian@gmail.com
3	Chang Park	MRCS	• Conceptualisation<br>• Methodology/Design<br>• Planning<br>• Supervise<br>• Data analysis<br>• Writing – reviewing and editing	changpark3@gmail.com
4	Khaled M Sarraf (senior author)	FRCS	• Conceptualisation<br>• Investigation<br>• Methodology/Design<br>• Planning<br>• Project administration<br>• Resources	khaled.sarraf@nhs.net

For peer review only

For peer review only - http://bmjopen.bmj.com/site/about/guidelines.xhtml

- Supervise
- Writing – reviewing and editing

COVERT Collaborators

Affiliation: Department of Trauma and Orthopaedics, Imperial College Healthcare NHS Trust, London, W2 1NY, England

	Name	Qualification	Contribution	E-mail
5	Thomas Ashdown	MRCS	• Data curation<br>• Formal analysis<br>• Resources	thomas.ashdown@nhs.net
6	Ramla Ali	MBBS	• Data curation<br>• Formal analysis<br>• Resources	ramla.ali@nhs.net
7	William Harland	MBBS	• Data curation<br>• Formal analysis<br>• Resources	william.harland@nhs.net
8	Charles Hallett	MBBS	• Data curation<br>• Formal analysis<br>• Resources	Ortho@pansurg.org
9	Chinmay Gupte	FRCS	• Project administration<br>• Resources<br>• Supervise	c.gupte00@ic.ac.uk
10	Dominic Spicer	FRCS	• Project administration<br>• Resources<br>• Supervise	dominic.spicer@nhs.net
11	Robin Strachan	FRCS	• Project administration<br>• Resources<br>• Supervise	robin.strachan@nhs.net

For peer review only

12	Dinesh Nathwani	FRCS	• Project administration<br>• Resources<br>• Supervise	d.nathwani@imperial.ac.uk
13	Rajarshi Bhattacharya	FRCS	• Project administration<br>• Resources<br>• Supervise	rajarshi.bhattacharya@nhs.net

Affiliation: Department of Trauma and Orthopaedics, Chelsea and Westminster Hospital NHS Foundation Trust, London, SW10 9NH, England

	Name	Qualification	Contribution	E-mail
14	Catrin Morgan	MRCS	• Data curation<br>• Formal analysis<br>• Project administration<br>• Supervise	catrin.morgan@nhs.net
15	Katharine Bennett-Brown	MRCS	• Data curation<br>• Formal analysis<br>• Resources	katebennettbrown@gmail.com
16	Henry Simon	MRCS	• Data curation<br>• Formal analysis<br>• Resources	henrysimon@hotmail.co.uk
17	Rupen Dattani	FRCS	• Project administration<br>• Resources<br>• Supervise	rupen.dattani@nhs.net

Affiliation: Department of Trauma and Orthopaedics, Queen Elizabeth Hospital, Gateshead Health NHS Foundation Trust, Gateshead, NE9 6SX, England

	Name	Qualification	Contribution	E-mail
18	Adham Mousa	MRCS	• Data curation<br>• Formal analysis<br>• Project administration	adham.mousa@nhs.net

For peer review only

			• Supervise	
19	Karen Vejsbjerg	MRCS	• Data curation<br>• Formal analysis<br>• Resources	karen.vejsbjerg1@nhs.net
20	Michael Zhang	MBBS	• Data curation<br>• Formal analysis<br>• Resources	michael.zhang1@nhs.net
21	Vikas Kumar	MBBS	• Data curation<br>• Formal analysis<br>• Resources	vikas.kumar7@nhs.net
22	Hannah Emerson	MBBS	• Data curation<br>• Formal analysis<br>• Resources	hannah.emerson2@nhs.net
23	Andrew Oliver	MBBS	• Data curation<br>• Formal analysis<br>• Resources	andrew.oliver2@nhs.net
24	Callum Smith	MBBS	• Data curation<br>• Formal analysis<br>• Resources	callum.smith8@nhs.net
25	Andreas Hinsche	FRCS	• Project administration<br>• Resources<br>• Supervise	a.hinsche@nhs.net

Affiliation: Department of Trauma and Orthopaedics, Darent Valley Hospital, Dartford and Gravesham NHS Trust, Dartford, DA2 8DA, England

	Name	Qualifi-cation	Contribution	E-mail
26	Markus L Sagmeister	MRCS	• Data curation<br>• Formal analysis<br>• Project administration<br>• Supervise	markus.sagmeister@nhs.net
27	Gihan Jayasinghe	MRCS	• Data curation<br>• Formal analysis<br>• Resources	gihan.jayasinghe@nhs.net

For peer review only - http://bmjopen.bmj.com/site/about/guidelines.xhtml

28	Marissa Lewis	MBBS	• Data curation<br>• Formal analysis<br>• Resources	marissa.lewis2@nhs.net
29	Michael Avery	MBBS	• Data curation<br>• Formal analysis<br>• Resources	michael.avery@nhs.net

Affiliation: Department of Trauma and Orthopaedics, Royal Berkshire NHS Foundation Trust, Reading, RG1 5AN, England

	Name	Qualifi-cation	Contribution	E-mail
30	Tariq Aboelmagd	MRCS	• Data curation<br>• Formal analysis<br>• Project administration<br>• Supervise	taboelmagd@doctors.org.uk
31	Shireen Ibish	MRCS	• Data curation<br>• Formal analysis<br>• Resources	shireen.ibish@nhs.net
32	Ruben Thumbadoo	MRCS	• Data curation<br>• Formal analysis<br>• Resources	ruben.thumbadoo@nhs.net
33	Asanka Wijendra	MRCS	• Data curation<br>• Formal analysis<br>• Resources	awijendra@gmail.com
34	Joseph Dixon	MRCS	• Data curation<br>• Formal analysis<br>• Resources	joedixon@doctors.org.uk
35	Vatsal Gupta	MRCS	• Data curation<br>• Formal analysis<br>• Resources	vatsalg001@gmail.com

Affiliation: Department of Trauma and Orthopaedics, James Cook University Hospital, South Tees NHS Foundation Trust, Middlesbrough, TS4 3BW, England

For peer review only - http://bmjopen.bmj.com/site/about/guidelines.xhtml

	Name	Qualification	Contribution	E-mail
36	Laura Clifton	MRCS	• Data curation<br>• Formal analysis<br>• Project administration<br>• Supervise	laura.clifton8@nhs.net
37	Suhib Taher	MRCS	• Data curation<br>• Formal analysis<br>• Resources	Suhib.taher@nhs.net
38	Nicholas Jia Jie Wei	MRCS	• Data curation<br>• Formal analysis<br>• Resources	nicholas.wei1@nhs.net
39	Emma Reay	FRCS	• Formal analysis<br>• Resources<br>• Supervise	emma.reay1@nhs.net

Affiliation: Department of Trauma and Orthopaedics, Aneurin Bevan University Health Board, Royal Gwent Hospital, Newport, NP20 2UB, Wales

	Name	Qualification	Contribution	E-mail
40	Ashish Khurana	FRCS	• Data curation<br>• Formal analysis<br>• Project administration<br>• Supervise	ashish.khurana@wales.nhs.uk
41	Kathryn Rooney	MRCS	• Data curation<br>• Formal analysis<br>• Resources	Kathryn.Rooney@wales.nhs.uk
42	Nakanda Kakaire	MRCS	• Data curation<br>• Formal analysis<br>• Resources	nakandamiriam.kakaire@wales.nhs.uk
43	Oluwawyemisi Adesina	MRCS	• Data curation<br>• Formal analysis<br>• Resources	quentinpz@yahoo.com

For peer review only

Figure 1: Types and mechanisms of injuries for acute referrals

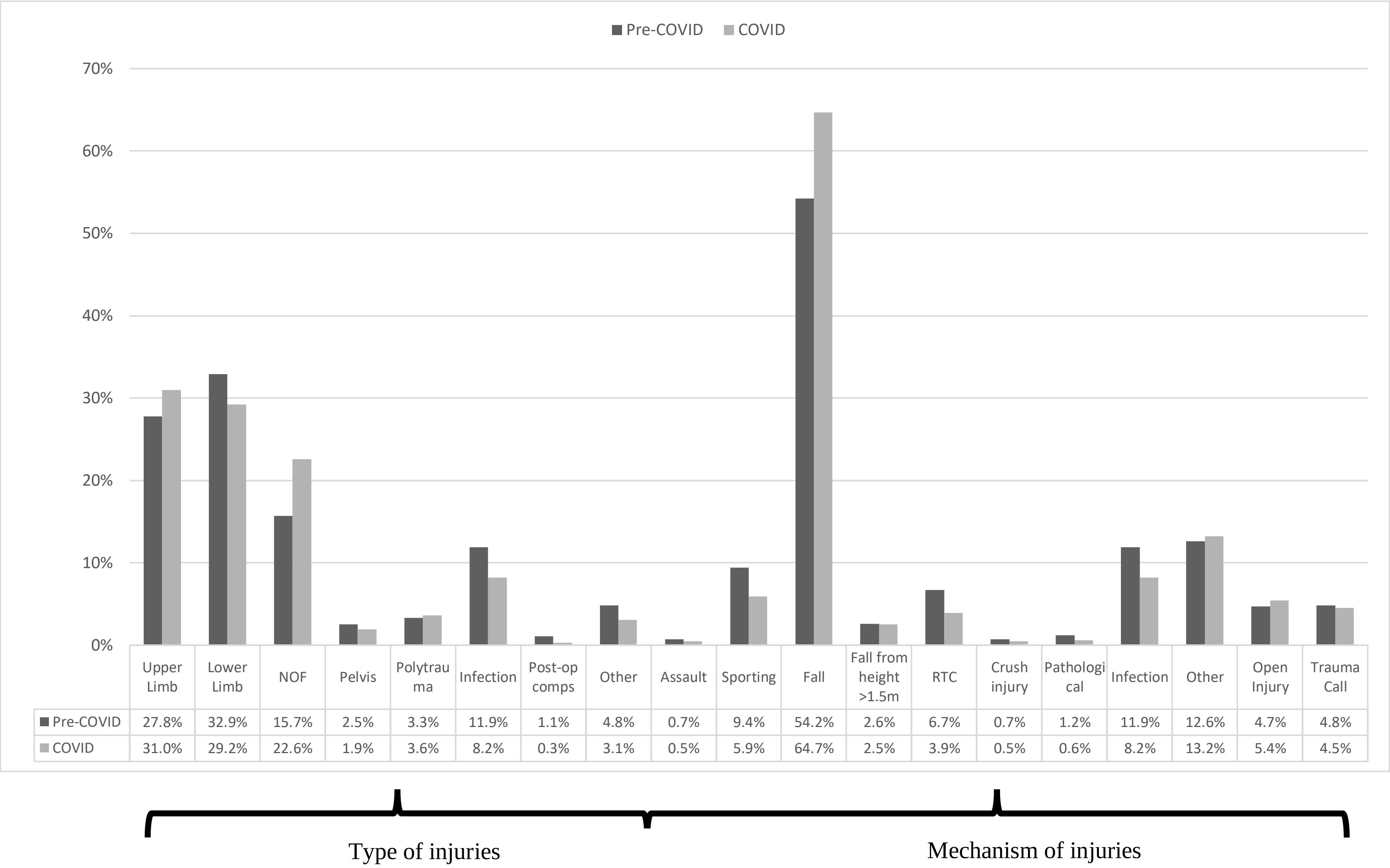

	Upper Limb	Lower Limb	NOF	Pelvis	Polytrauma	Infection	Post-op comps	Other	Assault	Sporting	Fall	Fall from height >1.5m	RTC	Crush injury	Pathological	Infection	Other	Open Injury	Trauma Call
Pre-COVID	27.8%	32.9%	15.7%	2.5%	3.3%	11.9%	1.1%	4.8%	0.7%	9.4%	54.2%	2.6%	6.7%	0.7%	1.2%	11.9%	12.6%	4.7%	4.8%
COVID	31.0%	29.2%	22.6%	1.9%	3.6%	8.2%	0.3%	3.1%	0.5%	5.9%	64.7%	2.5%	3.9%	0.5%	0.6%	8.2%	13.2%	5.4%	4.5%

Figure 2: Types and mechanisms of injuries for operative cases

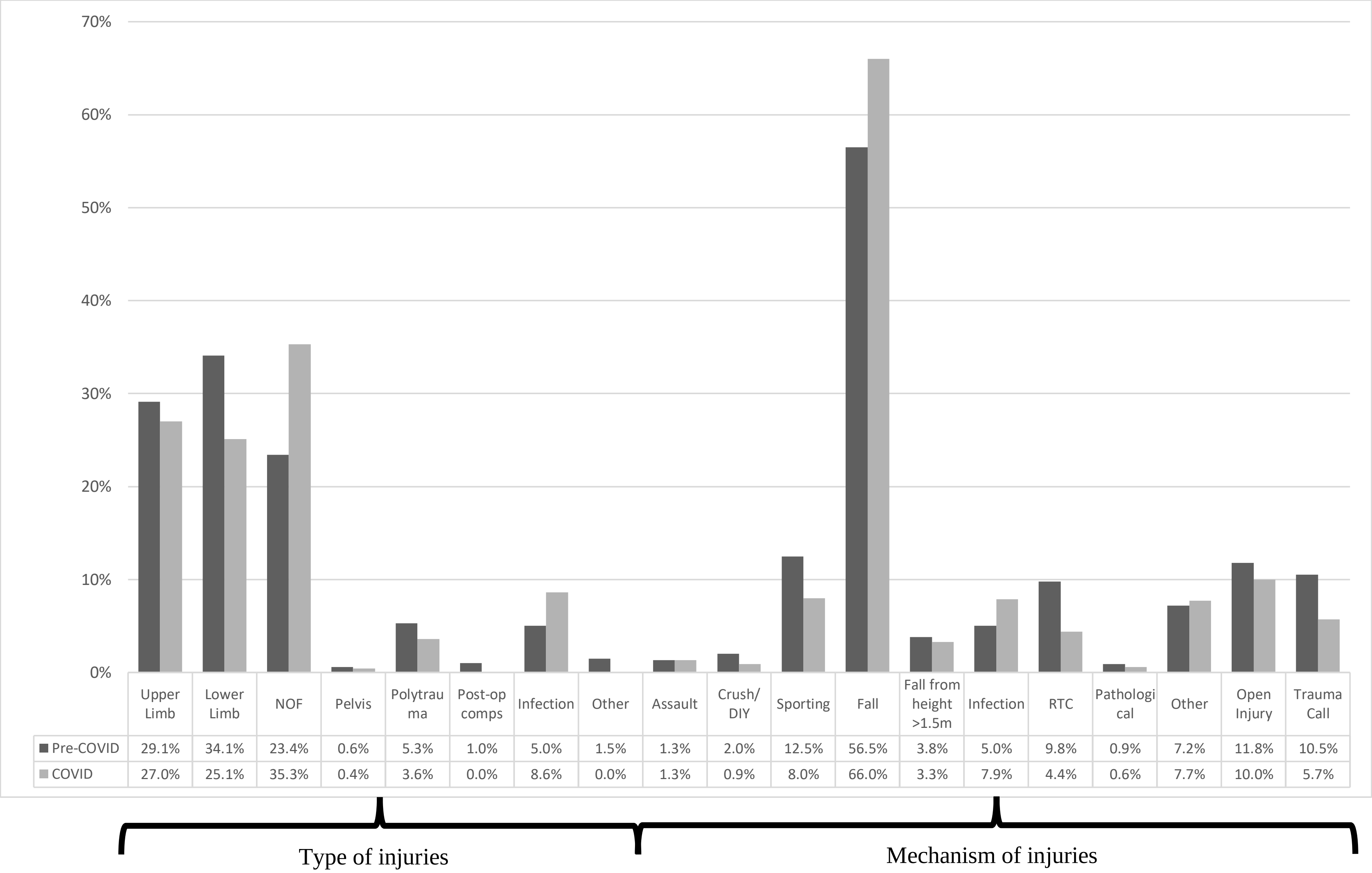

	Upper Limb	Lower Limb	NOF	Pelvis	Polytrauma	Post-op comps	Infection	Other	Assault	Crush/ DIY	Sporting	Fall	Fall from height >1.5m	Infection	RTC	Pathological	Other	Open Injury	Trauma Call
Pre-COVID	29.1%	34.1%	23.4%	0.6%	5.3%	1.0%	5.0%	1.5%	1.3%	2.0%	12.5%	56.5%	3.8%	5.0%	9.8%	0.9%	7.2%	11.8%	10.5%
COVID	27.0%	25.1%	35.3%	0.4%	3.6%	0.0%	8.6%	0.0%	1.3%	0.9%	8.0%	66.0%	3.3%	7.9%	4.4%	0.6%	7.7%	10.0%	5.7%

Figure 3: operative and anaesthetic techniques compared between pre- and post-COVID

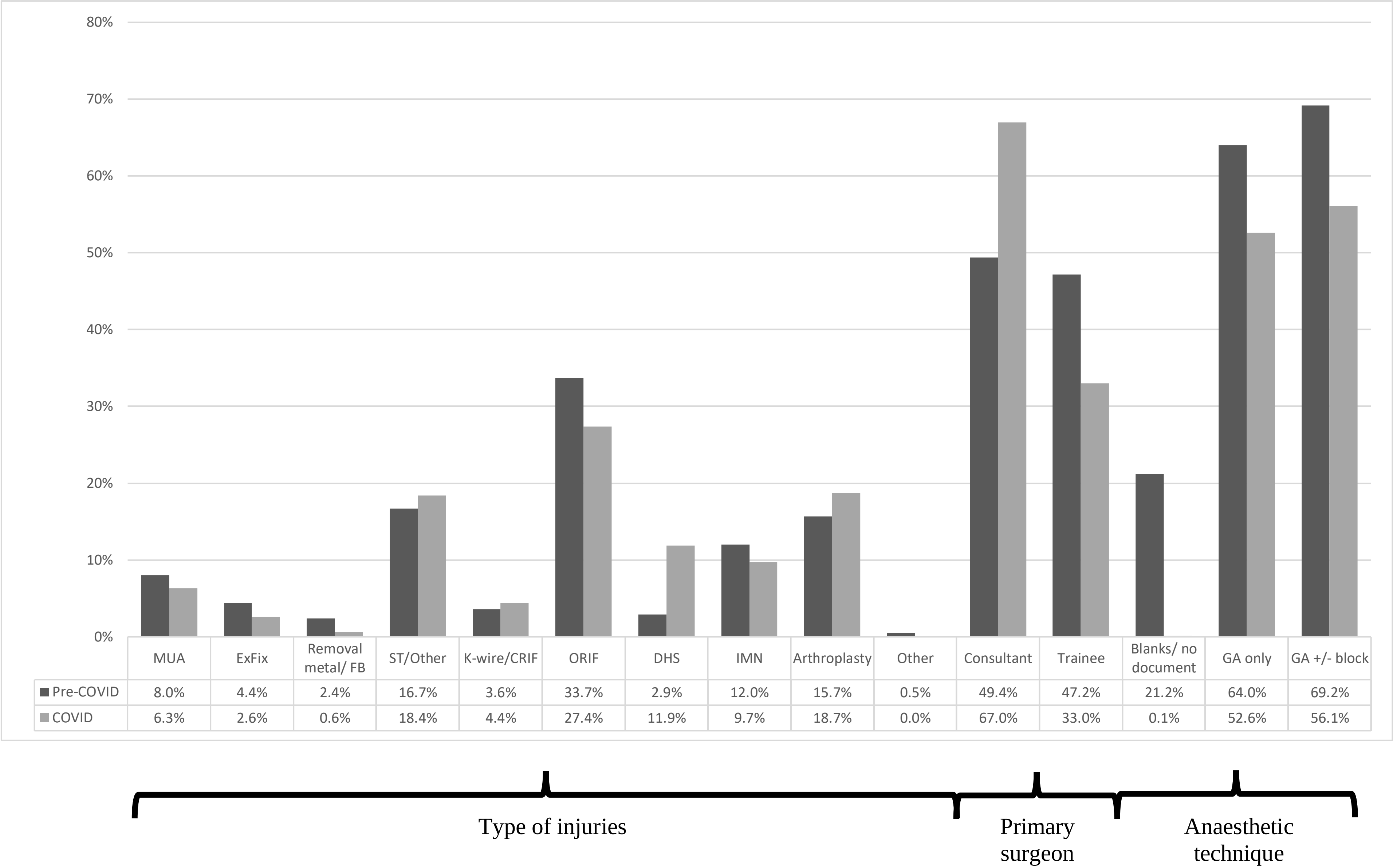

	MUA	ExFix	Removal metal/ FB	ST/Other	K-wire/CRIF	ORIF	DHS	IMN	Arthroplasty	Other	Consultant	Trainee	Blanks/ no document	GA only	GA +/- block
Pre-COVID	8.0%	4.4%	2.4%	16.7%	3.6%	33.7%	2.9%	12.0%	15.7%	0.5%	49.4%	47.2%	21.2%	64.0%	69.2%
COVID	6.3%	2.6%	0.6%	18.4%	4.4%	27.4%	11.9%	9.7%	18.7%	0.0%	67.0%	33.0%	0.1%	52.6%	56.1%

Figure 4: COVID status of both cohorts including positive results in all mortalities (with 5% error bars)

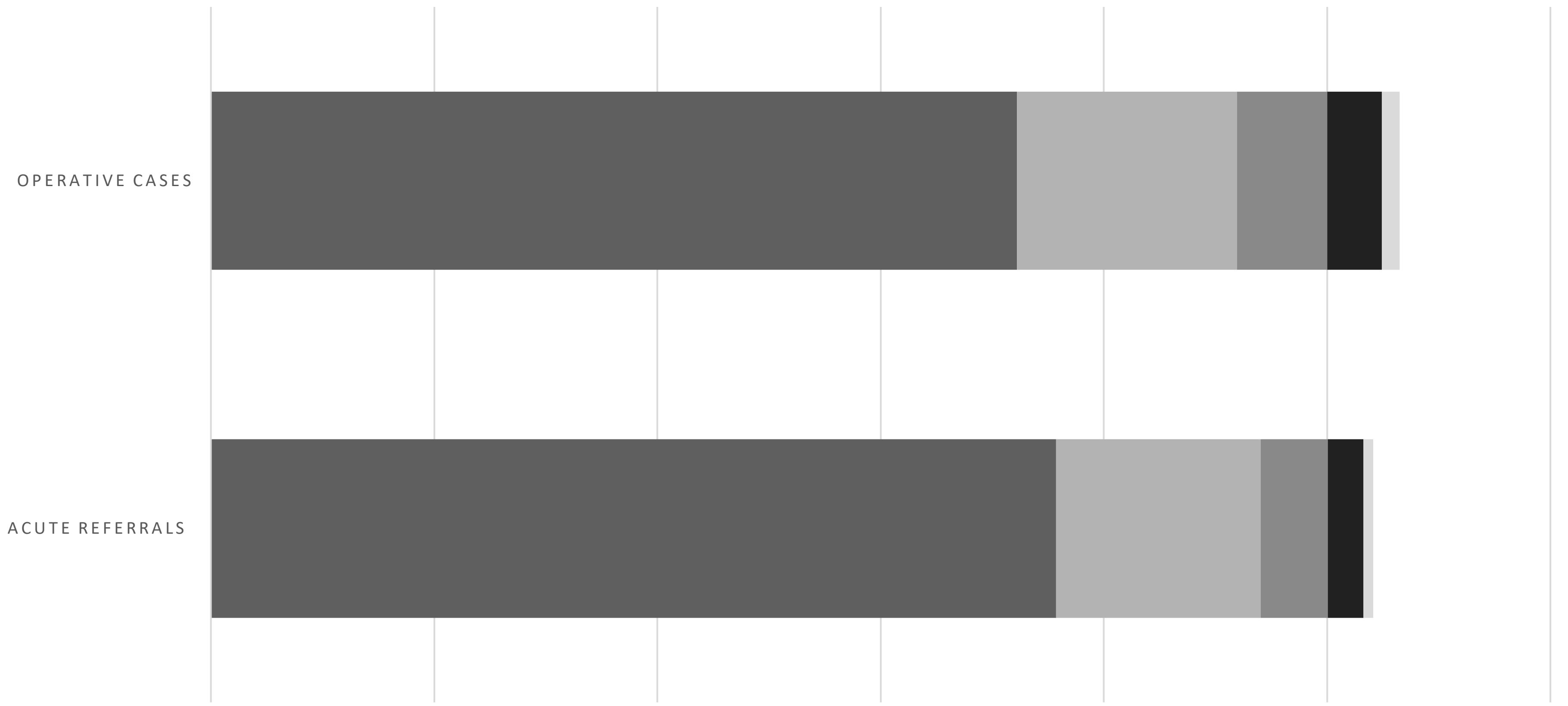

	Acute referrals	Operative cases
Not tested/asymptomatic/unknown	75.7%	72.2%
COVID -	18.3%	19.7%
COVID +	6.0%	8.1%
6-week Mortality	3.2%	4.9%
Mortality with COVID +	0.9%	1.6%

Figure 5: types and mechanism of injury for mortalities in acute referral cohorts

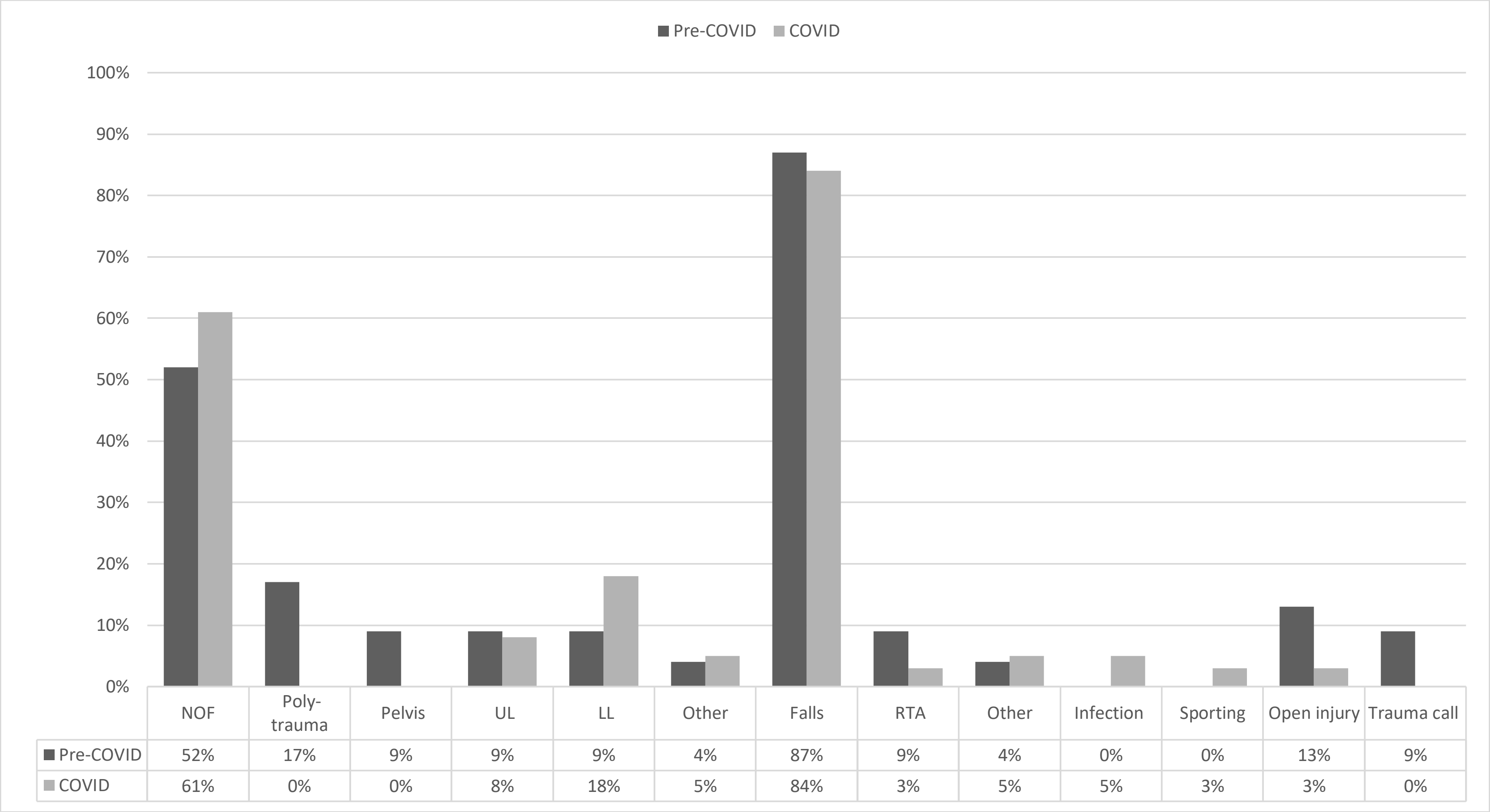

***Key**: UL: upper limb / LL: lower limb / RTA: road traffic accidents*

Figure 6: types and mechanism of injury for mortalities in operative casemix cohorts

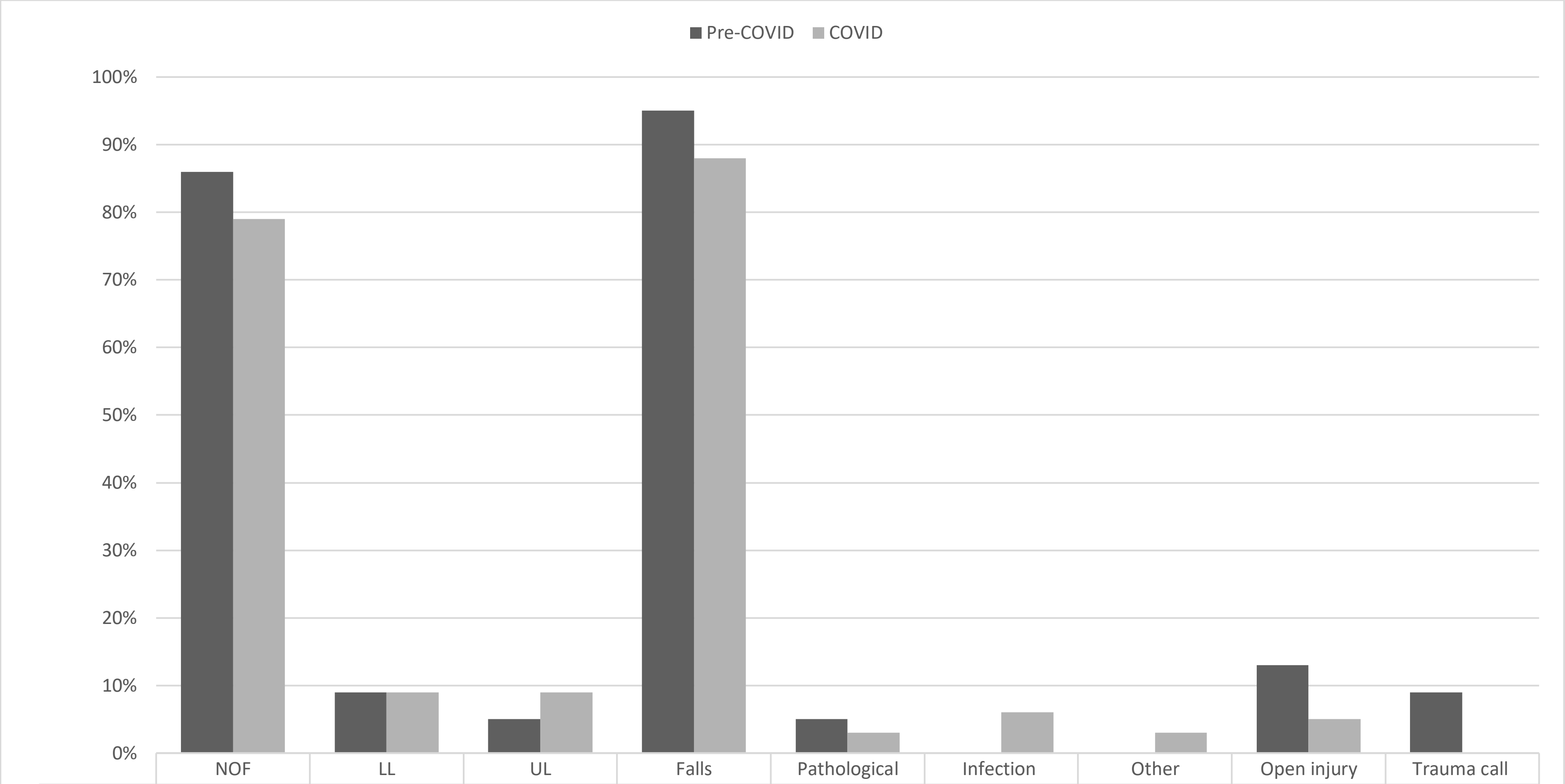

	NOF	LL	UL	Falls	Pathological	Infection	Other	Open injury	Trauma call
Pre-COVID	86%	9%	5%	95%	5%	0%	0%	13%	9%
COVID	79%	9%	9%	88%	3%	6%	3%	5%	0%

***Key**: NOF: neck of femur fracture / UL: upper limb / LL: lower limb*

Figure 7: Surgical and anaesthetic techniques utilised in mortalities as a means of proportions

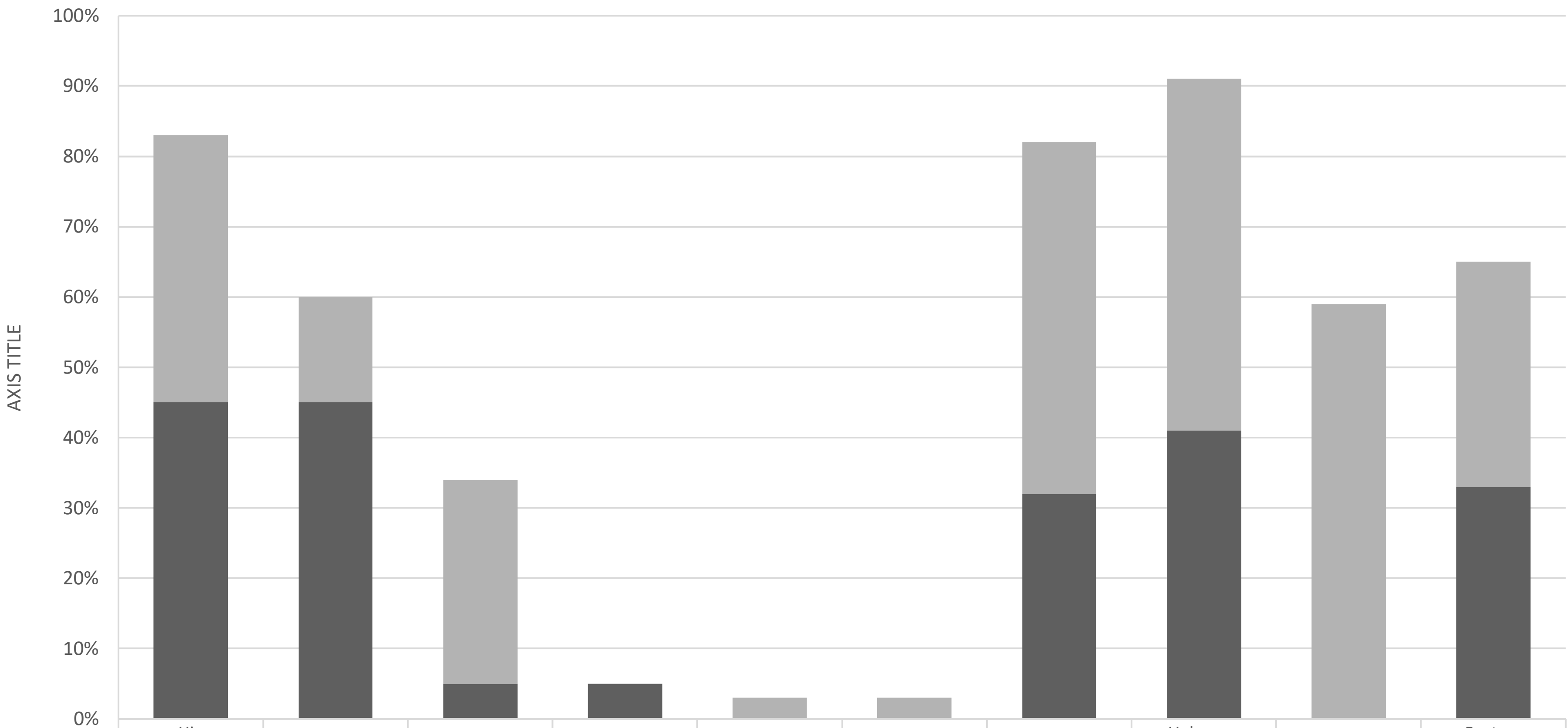

	Hip hemiarthroplasty	IMN	DHS	MUA	Soft tissue	Ex-Fix	AGP	Unknown anesthetic	Consultant-led	Post-op complications
COVID	38%	15%	29%	0%	3%	3%	50%	50%	59%	32%
Pre-COVID	45%	45%	5%	5%	0%	0%	32%	41%	0%	33%

***Key**: IMN: intramedullary nailing / DHS: dynamic hip screw / MUA: manipulation under anaesthesia / Ex-Fix: external fixation / AGP: aerosolizing-generating procedures*

Figure 8: Six-week Kaplan-Meier survival probability analysis for mortalities between pre- and post-COVID for acutely referred from the Emergency Department

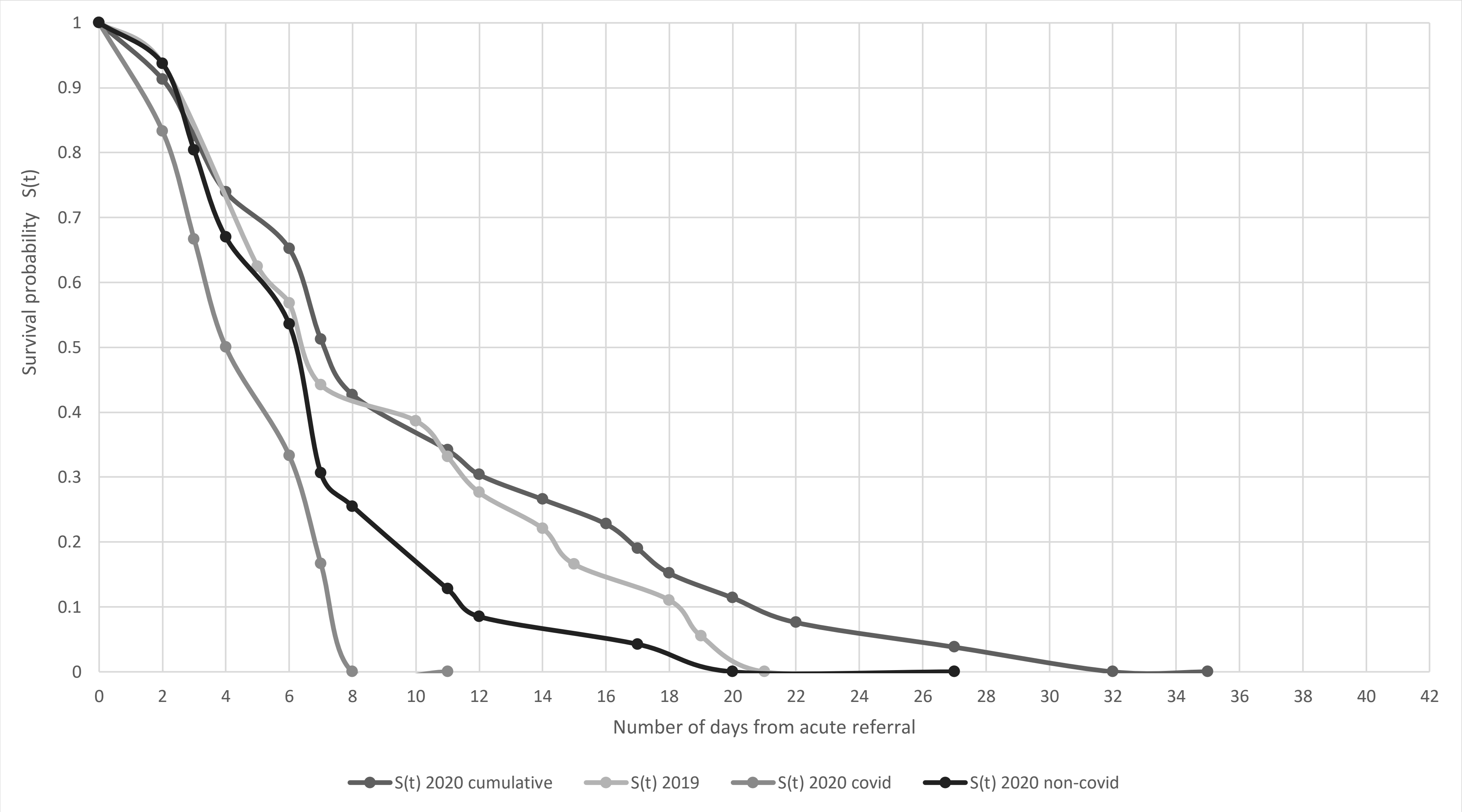

Figure 9: Six-week Kaplan-Meier survival probability analysis for mortalities between pre- and post-COVID for those undergoing surgery

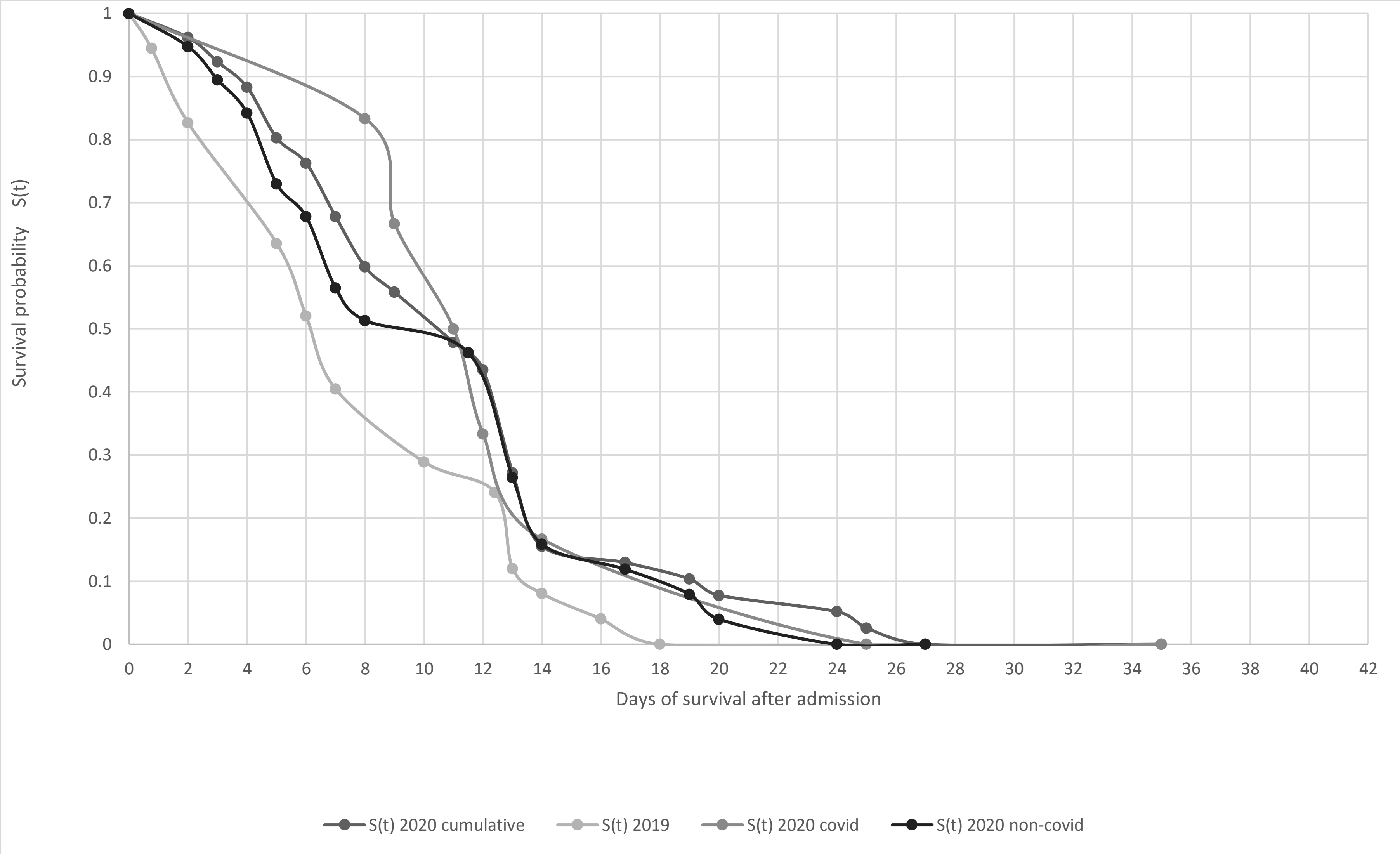

Appendix 1: Post-operative complications for both years

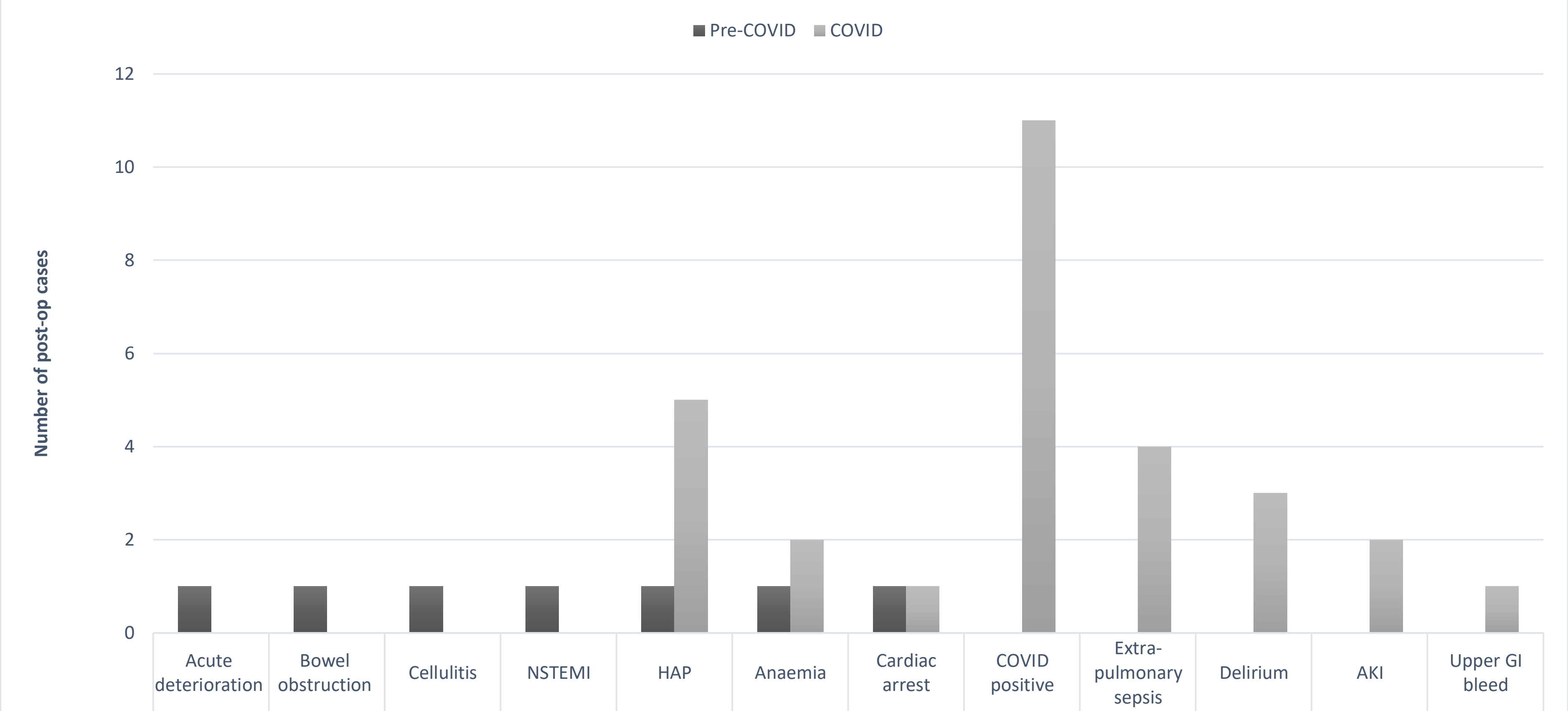

	Acute deterioration	Bowel obstruction	Cellulitis	NSTEMI	HAP	Anaemia	Cardiac arrest	COVID positive	Extra-pulmonary sepsis	Delirium	AKI	Upper GI bleed
Pre-COVID	1	1	1	1	1	1	1	0	0	0	0	0
COVID	0	0	0	0	5	2	1	11	4	3	2	1

***Key**: NSTEMI: non-ST elevated myocardial infarction, HAP: hospital-acquired pneumonia, AKI: acute kidney injury, GI: gastrointestinal*

Appendix 2: Type of comorbidities for all mortalities relative to both years

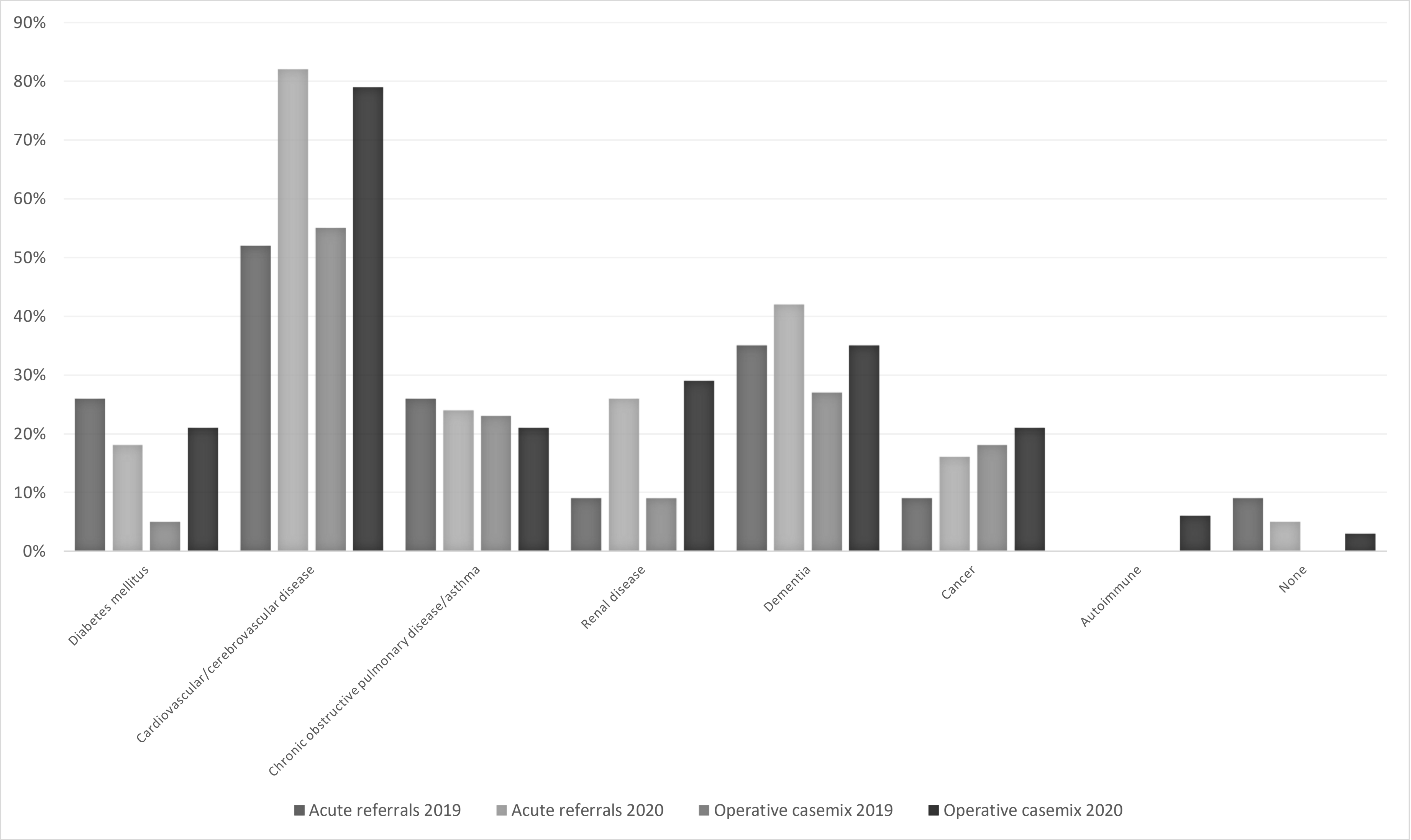

Appendix 3: number of comorbidities for all mortalities relative to both years

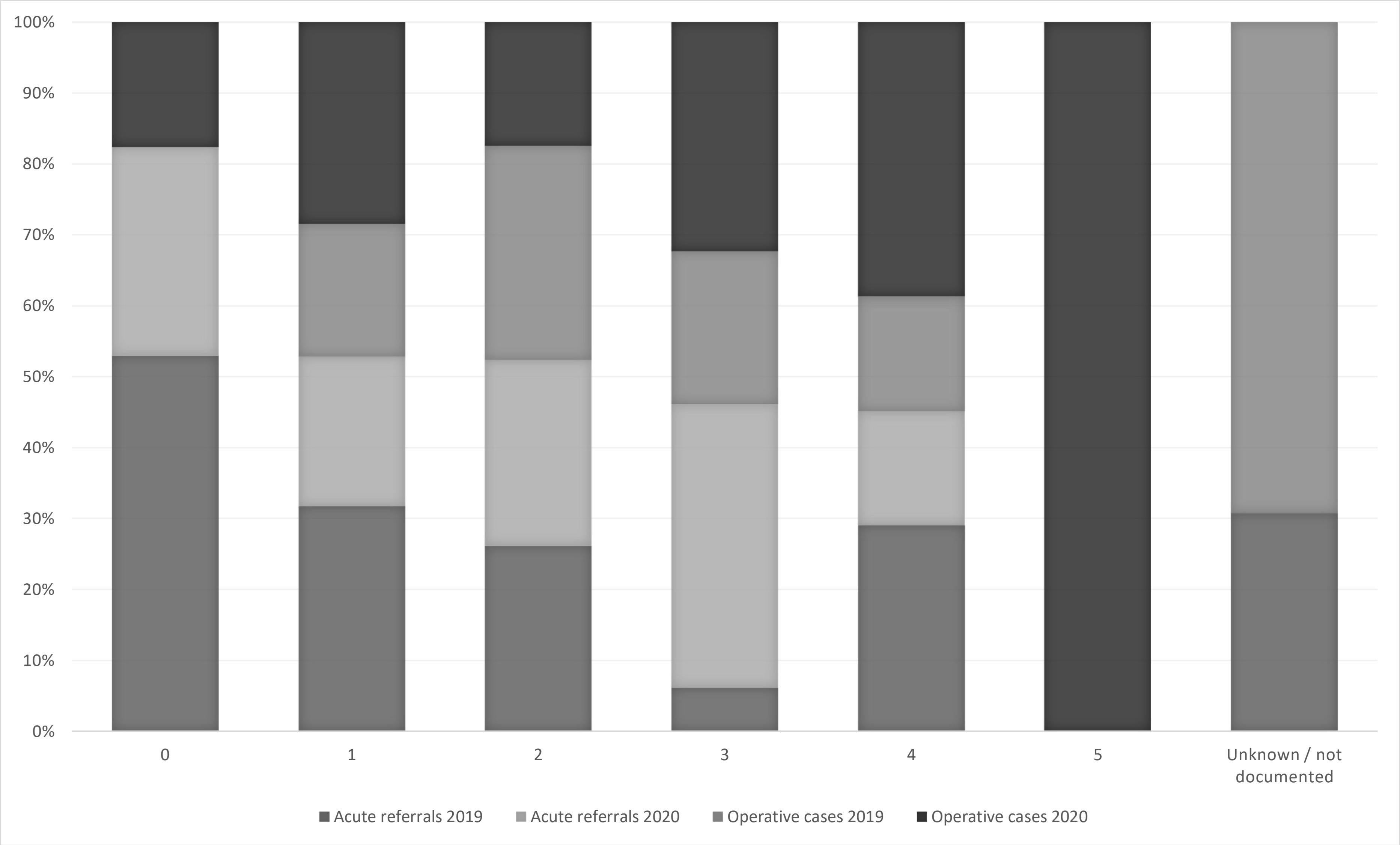

STROBE Statement—Checklist of items that should be included in reports of ***cohort studies***

	Item No	Recommendation	Page No
**Title and abstract**	1	(*a*) Indicate the study's design with a commonly used term in the title or the abstract	1, 7-8
		(*b*) Provide in the abstract an informative and balanced summary of what was done and what was found	8
**Introduction**			
Background/rationale	2	Explain the scientific background and rationale for the investigation being reported	9-10
Objectives	3	State specific objectives, including any prespecified hypotheses	10
**Methods**			
Study design	4	Present key elements of study design early in the paper	11
Setting	5	Describe the setting, locations, and relevant dates, including periods of recruitment, exposure, follow-up, and data collection	11-12
Participants	6	(*a*) Give the eligibility criteria, and the sources and methods of selection of participants. Describe methods of follow-up	12
		(*b*) For matched studies, give matching criteria and number of exposed and unexposed	12
Variables	7	Clearly define all outcomes, exposures, predictors, potential confounders, and effect modifiers. Give diagnostic criteria, if applicable	11-13 Table 1
Data sources/ measurement	8*	For each variable of interest, give sources of data and details of methods of assessment (measurement). Describe comparability of assessment methods if there is more than one group	11,14
Bias	9	Describe any efforts to address potential sources of bias	11-12
Study size	10	Explain how the study size was arrived at	11-12
Quantitative variables	11	Explain how quantitative variables were handled in the analyses. If applicable, describe which groupings were chosen and why	11-12, 14
Statistical methods	12	(*a*) Describe all statistical methods, including those used to control for confounding	14
		(*b*) Describe any methods used to examine subgroups and interactions	14
		(*c*) Explain how missing data were addressed	29
		(*d*) If applicable, explain how loss to follow-up was addressed	n/a
		(*e*) Describe any sensitivity analyses	n/a
**Results**			
Participants	13*	(a) Report numbers of individuals at each stage of study—eg numbers potentially eligible, examined for eligibility, confirmed eligible, included in the study, completing follow-up, and analysed	16 Table 2, Figs 1-3
		(b) Give reasons for non-participation at each stage	16
		(c) Consider use of a flow diagram	n/a
Descriptive data	14*	(a) Give characteristics of study participants (eg demographic, clinical, social) and information on exposures and potential confounders	16 Table 2, Figs 1-3
		(b) Indicate number of participants with missing data for each variable of interest	Figs 1-4
		(c) Summarise follow-up time (eg, average and total amount)	n/a

For peer review only

For peer review only - http://bmjopen.bmj.com/site/about/guidelines.xhtml

Outcome data	15*	Report numbers of outcome events or summary measures over time	Tables 2-4, Figures 1-12
Main results	16	(*a*) Give unadjusted estimates and, if applicable, confounder-adjusted estimates and their precision (eg, 95% confidence interval). Make clear which confounders were adjusted for and why they were included	9-15, Tables 2-4, Figs 1-12
		(*b*) Report category boundaries when continuous variables were categorized	n/a
		(*c*) If relevant, consider translating estimates of relative risk into absolute risk for a meaningful time period	Table 3
Other analyses	17	Report other analyses done—eg analyses of subgroups and interactions, and sensitivity analyses	20-21 Table 4 Figs 8-12
**Discussion**			
Key results	18	Summarise key results with reference to study objectives	22-29
Limitations	19	Discuss limitations of the study, taking into account sources of potential bias or imprecision. Discuss both direction and magnitude of any potential bias	29-30
Interpretation	20	Give a cautious overall interpretation of results considering objectives, limitations, multiplicity of analyses, results from similar studies, and other relevant evidence	22-29
Generalisability	21	Discuss the generalisability (external validity) of the study results	22-29
**Other information**			
Funding	22	Give the source of funding and the role of the funders for the present study and, if applicable, for the original study on which the present article is based	15

*Give information separately for exposed and unexposed groups.

Note: An Explanation and Elaboration article discusses each checklist item and gives methodological background and published examples of transparent reporting. Information on the STROBE Initiative is available at http://www.strobe-statement.org.

For peer review only
